# Evidence-based tailoring of bioinformatics approaches to optimize methods that predict the effects of nonsynonymous amino acid substitutions in glucokinase

**DOI:** 10.1038/s41598-017-09810-0

**Published:** 2017-08-25

**Authors:** Daniela Šimčíková, Lucie Kocková, Kateřina Vackářová, Miroslav Těšínský, Petr Heneberg

**Affiliations:** Charles University, Third Faculty of Medicine, Prague, Czech Republic

## Abstract

Computational methods that allow predicting the effects of nonsynonymous substitutions are an integral part of exome studies. Here, we validated and improved their specificity by performing a comprehensive bioinformatics analysis combined with experimental and clinical data on a model of glucokinase (GCK): 8835 putative variations, including 515 disease-associated variations from 1596 families with diagnoses of monogenic diabetes (*GCK*-MODY) or persistent hyperinsulinemic hypoglycemia of infancy (PHHI), and 126 variations with available or newly reported (19 variations) data on enzyme kinetics. We also proved that high frequency of disease-associated variations found in patients is closely related to their evolutionary conservation. The default set prediction methods predicted correctly the effects of only a part of the *GCK*-MODY-associated variations and completely failed to predict the normoglycemic or PHHI-associated variations. Therefore, we calculated evidence-based thresholds that improved significantly the specificity of predictions (≤75%). The combined prediction analysis even allowed to distinguish activating from inactivating variations and identified a group of putatively highly pathogenic variations (EVmutation score <−7.5 and SNAP2 score >70), which were surprisingly underrepresented among MODY patients and thus under negative selection during molecular evolution. We suggested and validated the first robust evidence-based thresholds, which allow improved, highly specific predictions of disease-associated GCK variations.

## Introduction

Glucokinase (GCK), which is one of the four mammalian isozymes that phosphorylate glucose, serves as a glucose sensor in pancreatic beta-cells, drives glucose conversion to glycogen in the liver and is expressed in the brain and endocrine cells of the gut^1^. GCK is considered a key enzyme in the glycolytic pathway, particularly because of the concentration of substrate at which this enzyme shows half-maximal activity (*S*_0.5_) that is within the physiological range of blood glucose concentrations^2^ and because of its kinetic cooperativity, which is unique among hexokinases. In healthy people, this allows GCK to tune its response following the uptake of glucose-containing food without completely depleting blood glucose levels^3^.

The inactivating GCK nonsynonymous substitutions cause maturity-onset diabetes of the young (*GCK*-MODY) or insulin-deficient hyperglycemia when only one allele is affected, and they cause severe permanent neonatal diabetes mellitus (PNDM) when both alleles are inactivated, whereas the activating nonsynonymous substitutions lead to persistent hyperinsulinemic hypoglycemia of infancy (PHHI). Hundreds of nonsynonymous substitutions of the *GCK* gene have been described in many populations. The heterozygously manifested inactivating nonsynonymous substitutions are usually only associated with mild fasting hyperglycemia. Thus, many patients are not diagnosed because there is an absence of symptoms; a higher perceived prevalence of *GCK*-MODY is known in countries that perform routine blood glucose screens on pregnant women or oral glucose tolerance test (OGTT) on asymptomatic young relatives within families with multiple cases of type 2 diabetes mellitus^4^. Characterization of GCK nonsynonymous substitutions is a laborious and time-consuming task. Thus, it prevents large-scale analyses for clinical purposes, particularly when considering a structural perspective. Recently, computational evolution- and structure-based prediction analyses were suggested to estimate the effects of particular GCK nonsynonymous substitutions^5^. These analyses aimed to identify nonsynonymous substitutions, which are likely or unlikely to have a serious impact on the protein function and stability. However, these analyses have not been paired with robust experimental data. Currently, experimental data are available based on *in vitro* kinetic analyses of over a hundred GCK nonsynonymous substitutions e.g., refs 6–9, and some nonsynonymous substitutions have been newly re-classified as non-pathogenic^10^.

In this study, we aimed to provide the first robust evidence for choosing the best-fit method and the evidence-based threshold to predict the effects of GCK nonsynonymous substitutions. For the first time, we compared the outcomes of prediction methods with the outcomes of *in vitro* measurements reported previously or reported newly in the course of this study, and with clinical information known from patients carrying GCK nonsynonymous substitutions. We calculated the evidence-based thresholds in order to solve the problems with negligible specificity of their previously suggested arbitrary values. By the analysis of total hypothetical GCK nonsynonymous substitutions, we predicted the effects of GCK nonsynonymous substitutions for which the clinical or *in vitro* data are still absent.

## Results

### *In vitro* enzyme kinetics

We analyzed the enzyme kinetics of 16 naturally occurring GCK nonsynonymous substitutions known from MODY patients of Czech origin and the experimental nonsynonymous substitutions R63S, M251C and F260L. Five mutants – that included four naturally occurring *GCK*-MODY-associated mutants (G81D, M251I, G295D and G385W) and one experimental mutant (M251C) – displayed no or negligible activity at ≤150 mM glucose. Regarding the other mutants, the *GCK*-MODY-associated mutants M251V, L314P, F316V, G318R and F419L demonstrated a reduced affinity for glucose that was expressed as an elevated *S*_0.5_ measured at 5 mM ATP (one-way ANOVA p < 0.001, F = 808.0, Tukey’s post-tests p < 0.05, excluding the enclosed means). However, when measured at the suboptimal ATP concentration (500 μM), only a partially overlapping set of *GCK*-MODY-associated mutants (F150L, M251V and A454E) exhibited an elevated *S*_0.5_ (one-way ANOVA p < 0.001, F = 172.3, Tukey’s post-tests p < 0.05, excluding the enclosed means). There was no overlap between those differing significantly from WT-GCK at high and low levels of ATP (Table 1). The Hill coefficient differed among the tested mutants (one-way ANOVA p < 0.001, F = 4.506), but we did not find any significant differences between the mean Hill coefficient of WT-GCK and any of the mutants (all Tukey’s post-tests of WT vs. mutants felt into the category of enclosed means). The ATP *K*_M_ was high particularly for F150L (i.e., four-times the control value), and it was also significantly increased in A454 and V33A (one-way ANOVA p < 0.001, F = 212.3, Tukey’s post-tests p < 0.05, excluding the enclosed means). The tested mutants displayed a wide range of *k*_cat_. Two of the MODY-associated mutants (V33A and F316V) exhibited *k*_cat_ values similar to WT-GCK, and all the others demonstrated decreased *k*_cat_ values compared to WT-GCK. Among the nonsynonymous substitutions tested, we did not find any that had a decreased stability at 30 °C (Table 1). Two nonsynonymous substitutions demonstrated a decreased susceptibility to the competitive inhibitor of the GCK activity, *N*-acetylglucosamine (GlcNAc). The majority of the nonsynonymous substitutions did not demonstrate any change in IC_50_ of GlcNAc compared to WT-GCK, except for F150L (>6-fold higher IC_50_) and A454E (2-fold higher IC_50_) (Table 1).

**Table 1:** The kinetic data for WT-GCK, 16 naturally occurring GCK nonsynonymous substitutions associated with MODY patients and the experimental nonsynonymous substitutions R63S, M251C and F260L.

Variation	*S*_0.5_ (at 5 mM ATP) [mM glucose]	*n*_H_	*S*_0.5_ (at 500 μM ATP) [mM glucose]	ATP *K*_M_ (at *S*_0.5_) [mM ATP]	ATP *K*_M_ (at 50 mM glucose) [mM ATP]	Stability [%]	*k*_cat_ [s^−1^]	GlcNAc IC_50_ [μM]	RAI	GSIR-T [mM glucose]
Wild type	8.82 ± 0.07	1.72 ± 0.04	5.65 ± 0.22	0.36 ± 0.00	0.47 ± 0.01	86 ± 1	43.8 ± 3.4	223 ± 19	1.00	5.0
V33A	12.77 ± 0.26	1.56 ± 0.05	5.75 ± 0.21	0.53 ± 0.03	0.63 ± 0.02	76 ± 3	42.6 ± 7.4	303 ± 14	0.51	5.9
R63S	4.44 ± 0.17	1.64 ± 0.04	2.75 ± 0.08	0.23 ± 0.02	0.35 ± 0.02	83 ± 0	57.7 ± 8.8	487 ± 35	4.17	2.9
G81D			No activity at ≤150 mM glucose						<0.01	≥7.1
F150L	20.05 ± 0.80	1.27 ± 0.04	17.83 ± 0.28	1.63 ± 0.02	2.15 ± 0.08	92 ± 4	13.2 ± 1.0	1470 ± 75	0.13	6.9
T209K	9.32 ± 0.25	1.53 ± 0.04	7.28 ± 0.26	0.25 ± 0.01	0.31 ± 0.01	88 ± 3	25.9 ± 5.7	293 ± 9	0.59	5.7
R250C	7.94 ± 0.25	1.57 ± 0.07	4.84 ± 0.27	0.37 ± 0.02	0.45 ± 0.01	81 ± 4	36.1 ± 6.0	287 ± 7	0.94	5.0
M251C	55.33 ± 1.38	1.38 ± 0.09	Activity close to detection limits				3.30 ± 0.15	N/D	<0.01	≥7.1
M251I	113.43 ± 20.7	1.33 ± 0.09	Activity close to detection limits				10.18 ± 0.95	N/D	<0.01	≥7.1
M251V	46.4 ± 2.11	1.59 ± 0.03	42.36 ± 2.24	0.46 ± 0.02	0.51 ± 0.00	90 ± 5	7.6 ± 1.7	N/D	0.01	7.1
C252R	8.37 ± 0.19	1.61 ± 0.02	6.52 ± 0.37	0.27 ± 0.01	0.34 ± 0.02	78 ± 3	11.8 ± 1.9	290 ± 16	0.27	6.2
F260L	9.07 ± 0.19	1.54 ± 0.02	4.76 ± 0.05	0.36 ± 0.01	0.41 ± 0.01	82 ± 3	41.8 ± 6.5	263 ± 24	0.95	5.1
G295D			No activity at ≤150 mM glucose						<0.01	≥7.1
L314P	13.63 ± 0.84	1.4 ± 0.10	N/A	N/A	0.30 ± 0.00	N/D	5.9 ± 1.0	N/D	0.11	6.9
F316V	11.20 ± 0.50	1.66 ± 0.09	5.92 ± 0.26	0.47 ± 0.02	0.69 ± 0.04	84 ± 2	45.5 ± 4.1	250 ± 17	0.51	5.7
G318R	8.53 ± 0.21	1.63 ± 0.02	4.67 ± 0.15	0.37 ± 0.02	0.53 ± 0.02	84 ± 2	34.0 ± 7.6	293 ± 22	0.67	5.3
G385W			Activity close to detection limits				0.14 ± 0.06	N/D	<0.01	≥7.1
F419L	15.00 ± 0.32	1.67 ± 0.01	9.57 ± 0.21	0.33 ± 0.02	0.43 ± 0.02	84 ± 3	25.4 ± 1.1	277 ± 19	0.19	6.6
C434Y	7.36 ± 0.05	1.66 ± 0.03	4.50 ± 0.09	0.37 ± 0.02	0.45 ± 0.01	95 ± 3	29.0 ± 3.8	217 ± 14	0.71	5.1
A454E	24.49 ± 0.59	1.39 ± 0.04	14.06 ± 0.50	1.49 ± 0.07	1.36 ± 0.06	90 ± 3	6.7 ± 0.7	513 ± 20	0.04	7.0

Data are shown as the means ± SE and are representative of three to five preparations of each nonsynonymous substitution with three technical replicates analyzed for each preparation.

Based on the measured data, we calculated the relative activity index (RAI) and the threshold for glucose-stimulated insulin release (GSIR-T) of each tested protein variation. While many demonstrated RAIs at or below 10% of the activity of WT-GCK, there were also MODY-associated protein variations that did not display any major difference in the RAI compared to WT-GCK. These included R250C (RAI 0.94), C434Y (RAI 0.71) and G318R (RAI 0.67). Near-normal levels of RAI resulted in mild changes of GSIR-T associated with these three nonsynonymous substitutions; the GSIR-T only ranged from 5.0 to 5.3 mM glucose. Changes in the enzyme kinetics of the other MODY-associated mutants led to changes in the RAI within the range from 5.7 to 7.1 mM glucose, which is characteristic for the *GCK*-MODY phenotype.

Within the variations tested, there were also three experimentally designed nonsynonymous substitutions. These included the newly identified activating mutant, R63S, which demonstrated the RAI of 4.17 and the GSIR-T of 2.9 mM and led to a marked decrease in both, *S*_0.5_ and ATP *K*_M_ and an increased IC_50_ of GlcNAc (Table 1). Another experimental mutant was F260L, which demonstrated a neutral phenotype, reaching the RAI of 0.95, the GSIR-T of 5.1 mM, a marginally decreased *S*_0.5_ and ATP *K*_M_ and a similar *k*_cat_ and IC_50_ of GlcNAc compared to WT-GCK. The last experimental mutant, M251C, exhibited a strong deactivating effect with barely detectable activity even at high doses of glucose, which led to an estimated RAI < 0.01 and GSIR-T ≥ 7.1 mM (Table 1).

### Can prediction methods predict enzyme kinetics of GCK and are they in agreement with clinical data?

**Figure 1:**
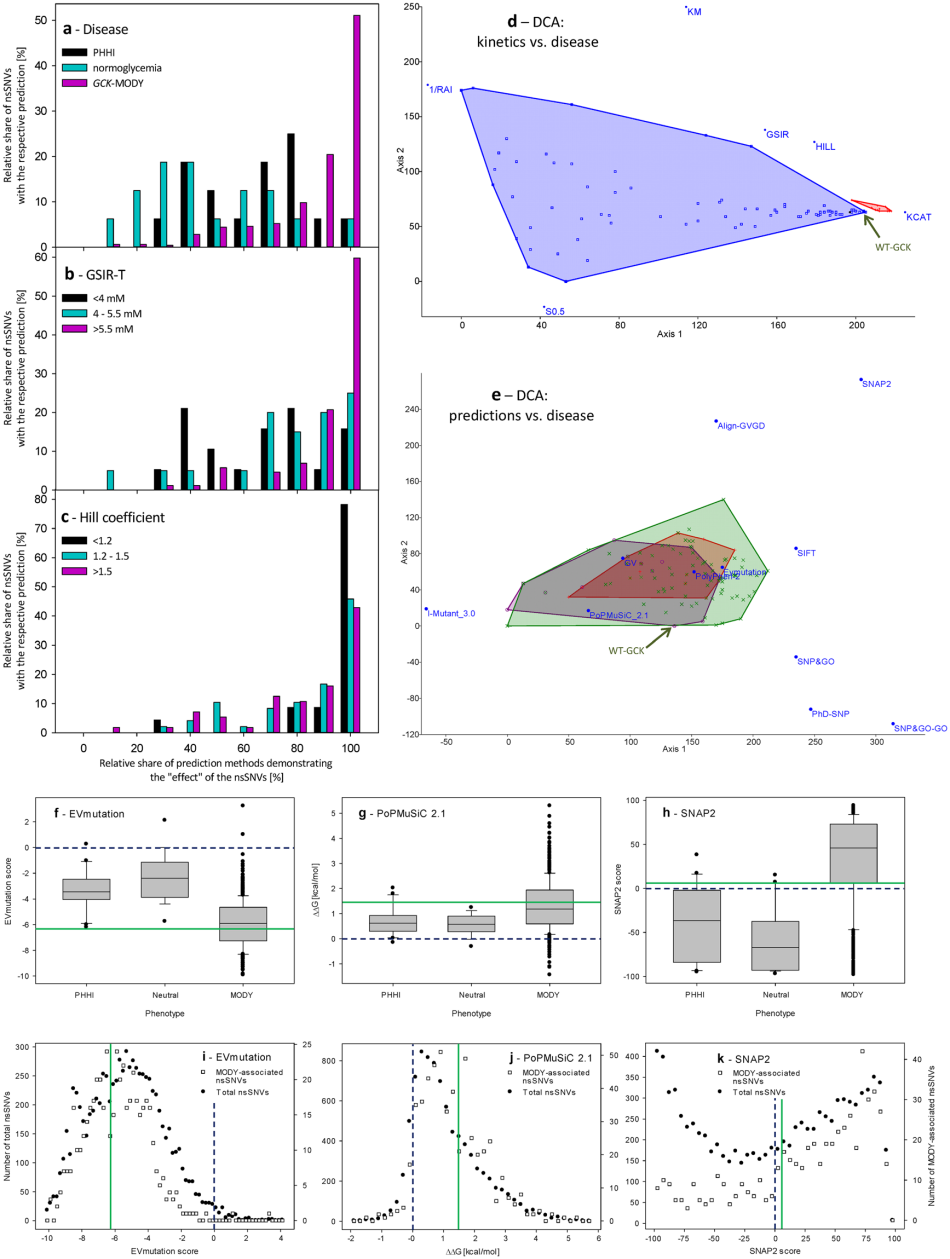
The efficiency of the prediction methods in predicting the effects of nonsynonymous substitutions in GCK on their enzyme kinetics and clinical phenotypes. (**a**–**c**) Number of prediction methods demonstrating the “effect” of the respective nonsynonymous substitution plotted against (**a**) the clinical phenotype of their heterozygous carriers, (**b**) the GSIR-T and (**c**) the Hill coefficient. (**d**,**e**) DCA comparing the nonsynonymous substitutions that were sorted according to their phenotype when plotted against (**d**) the outcomes of *in vitro* enzyme kinetics characterization (*GCK*-MODY = blue; PHHI = red polygon) and (**e**) predictions obtained using the state-of-the-art prediction methods (*GCK*-MODY = green; PHHI = red; normoglycemic = violet). Positions of WT-GCK are labeled with the green arrow. (**f**–**k**) The distribution of numerical scores of EVmutation, PoPMuSiC 2.1 and SNAP2 applied to GCK nonsynonymous substitutions with known clinical phenotype and of all putative GCK nonsynonymous substitutions. (**f**–**h**) The distribution of numerical scores of prediction methods applied to GCK nonsynonymous substitutions with known clinical phenotype. The data are shown for (**f**) EVmutation, (**g**) PoPMuSiC 2.1 and (**h**) SNAP2. The Tukey box plots are shown, the mean values are presented as lines, and the 5^th^ and 95^th^ percentiles are displayed as symbols. (**i**,**k**) The distribution of scores of EVmutation (**i**), PoPMuSiC 2.1 (**j**) and SNAP2 (**k**) as calculated for total putative GCK nonsynonymous substitutions and MODY-associated nonsynonymous substitutions. The pre-set (original) thresholds are highlighted with dashed dark-blue lines and newly defined evidence-based thresholds are highlighted with solid green lines (**f**–**k**).

The prediction methods demonstrated a generally high sensitivity for deleterious MODY-associated nonsynonymous substitutions, and the sensitivity of all but one method reached at least 75%. However, many methods exhibited low sensitivity when attempting to predict the hypoglycemic phenotype, for which only PoPMuSiC 2.1 and PolyPhen-2 had sensitivity over 90%. However, high sensitivity of the latter two methods was associated with detrimental outcomes when predicting the neutral non-diabetic nonsynonymous substitutions because they reached a sensitivity of only 27 and 38%, respectively. The other prediction methods were more sensitive when predicting normoglycemic nonsynonymous substitutions and were associated with up to 94% sensitivity (SNPs&GO with GO terms excluded). However, when there was a high sensitivity for the nonsynonymous substitutions with neutral phenotypes, the false-positive ratio for detection of such nonsynonymous substitutions among those associated with disease phenotypes was increased. SNPs&GO with GO terms excluded had 28% false positive ratio for predicting nonsynonymous substitutions with the neutral phenotype. Given that the number of known normoglycemic nonsynonymous substitutions is lower by one order of magnitude compared with the disease-associated nonsynonymous substitutions of GCK, the actual number of false-positive hits suggested by SNPs&GO exceeded the number of true positive hits regardless of the inclusion of GO terms (Table 2). Previously, it was suggested to use the prediction methods in combination^5^. However, the combination of the state-of-the-art prediction methods did not lead to any improvement in the prediction of the physiologic effects of nonsynonymous substitutions when referring to the healthy vs. disease-associated status of their heterozygous carriers, particularly when one or more predictors were in disagreement (Fig. 1a).

**Table 2:** The summary of prediction scores for nonsynonymous substitutions in the GCK amino acid sequence, for which data were available either on their clinical phenotype, the GSIR-T or the Hill coefficient. For a detailed list of nonsynonymous substitutions analyzed, relevant raw data and references, cf. Table S2.

Measure	Prediction method: Variable	SIFT	PolyPhen2	PhD-SNP	PoPMuSiC 2.1	SNAP2	SNPs&GO (GO terms excluded)	SNPs&GO (GO terms included)	I-Mutant 3	Align GVGD	EVmutation
**Disease**											
PHHI (n = 16)	Sensitivity [%]	63	94	44	94	25	13	56	81	81	75
Non-diabetic (n = 16)	Sensitivity [%]	75	38	75	27	88	94	50	20	50	N/A (53)
Non-diabetic (n = 515)	False positive ratio [%]	13.2	5.2	15.7	5.5	23.7	28.0	11.1	15.5	9.5	3.6
MODY (n = 499)	Sensitivity [%]	87.6	94.8	85.6	94.5	78.0	66.9	87.0	84.6	75.6	97.1
**GSIR-T**											
<4 (n = 19)	Sensitivity [%]	63	95	53	100	32	13	39	84	84	72
4–5.5 (n = 20; normal range)	Sensitivity [%]	25	10	35	15	40	55	30	20	15	5
4–5.5 (n = 106)	False positive ratio [%]	28	3	15	4	27	29	16	14	7	8
>5.5 (n = 87)	Sensitivity [%]	92	98	93	95	82	71	89	86	95	97
**Hill coefficient** ***n***_**H**_											
<1.2 (n = 23)	Sensitivity [%]	96	91	96	100	87	74	96	87	96	100
1.2–1.5 (n = 48)	Sensitivity [%]	83	96	79	94	69	50	77	88	94	94
>1.5 (n = 56; normal range)	Sensitivity [%]	18	4	23	5	36	39	23	14	11	11
>1.5 (n = 71)	False positive ratio [%]	13	4	15	4	25	30	15	13	6	4

PoPMuSiC2.1 and I-Mutant 3 were only calculated for amino acids available in the crystal structure (PDB ID: 1V4S). Calculations for some amino acids were not available for the EVmutation. The threshold value for EVmutation was set to a median value of normoglycemic variations (−2.39) as all but two neutral nonsynonymous substitutions exceeded the originally suggested zero threshold^11^.

The prediction methods demonstrated similar issues when tested against the GSIR-T values, which were calculated based on the experimentally measured enzyme kinetics data. Multiple methods had high sensitivities for both the activating and deactivating nonsynonymous substitutions, which were quantified as those with a GSIR-T lower than 4 mM or exceeding 5.5 mM, respectively. However, they failed to identify those within the normal range (4.0–5.5 mM glucose), which does not lead to PHHI, and is also below the threshold for diabetes. The most sensitive method for the neutral effects was SNPs&GO with GO terms excluded (55% sensitivity). However, the same method was associated with 29% false positive ratio for neutral predictions among nonsynonymous substitutions that cause disease-associated GSIR-T, and it was also associated with only 71% sensitivity for the deactivating nonsynonymous substitutions (Table 1). The combination of predictors was still associated with a poor ability to discriminate between a low and normal GSIR-T and with relatively low number of false-negative predictions among the nonsynonymous substitutions with a high GSIR-T (Fig. 1b).

One of the key features of GCK is its cooperativity. The majority of the tested prediction methods were able to correctly predict extreme changes in the Hill coefficient, which lead to a nearly complete loss of the cooperativity (*n*_H_ < 1.2) (Table 1). The combination of predictors was able to identify correctly those inducing complete or nearly complete loss of the cooperativity. However, the predictions were associated with a high number of false-positive results, and the only useful predictions of the absence of severe effects on the Hill coefficient were when seven or less algorithms agreed on the effect of the respective nonsynonymous substitution (Fig. 1c).

Reflecting the above unsatisfactory outcomes of the prediction methods, we analyzed whether the clinical phenotype of the patients can be predicted at all. First, we analyzed the relationship between the disease and experimentally measured enzyme kinetics of GCK by employing detrended correspondence analysis (DCA; Fig. 1d). The analysis involved six basic parameters for the enzyme kinetics, namely *S*_0.5_, *n*_H_, ATP *K*_M_, *k*_cat_, the RAI and the GSIR-T. The eigenvalues were 0.557 (axis 1), 0.111 (axis 2) and 0.026 (axis 3). At the molecular level, functional GCK variations are associated with two groups of phenotypes, the activating phenotypes (that manifest as PHHI) and the inactivating phenotypes (that manifest as *GCK*-MODY or PNDM). DCA distinguished between the MODY-associated and PHHI-associated nonsynonymous substitutions, and WT-GCK was positioned close to the left boundary of the MODY-associated area. The *k*_cat_ and RAI were responsible for most of the variability; fine resolution was allowed by the inclusion of *S*_0.5_ and ATP *K*_M_, which were associated with axis 2 (Fig. 1d). In contrast to the above comparison was the DCA of the nonsynonymous substitutions known from humans with the prediction methods that were suggested previously to predict the effects of GCK nonsynonymous substitutions^5, 11^ (Fig. 1e). The eigenvalues were 0.053 (axis 1), 0.014 (axis 2) and 0.013 (axis 3). The prediction methods with arbitrarily set thresholds did not correctly identify the effects of nonsynonymous substitutions as suggested by low eigenvalues and the overlap of convex hulls assigned to benign, activating and deactivating nonsynonymous substitutions (Fig. 1e). The number of predictors that correctly predicted the effect of each disease-associated nonsynonymous substitution, did not display any strong association with the clinical parameters measured in the affected patients, namely the levels of plasma glucose, glucose after 120 min OGTT, HbA_1c_, C-peptide and age at diagnosis (Fig. S1).

In order to improve the predictions, we tested, whether there is a space for the evidence-based adjustments of the arbitrary thresholds of the prediction methods. We found that with evidence-based thresholds, three methods were capable of distinguishing between a group of disease-associated nonsynonymous substitutions and those with uncertain phenotypes. Namely, the EVmutation scores of normoglycemic nonsynonymous substitutions reached −2.39 (95% CI −3.30–−1.32; Fig. 1f). When setting the threshold values to a median minus 2-times SD (EVmutation = −6.31), 43% of MODY-associated nonsynonymous substitutions (but no PHHI-associated nonsynonymous substitutions) passed this threshold applied to the outcomes of the EVmutation. Similarly, PoPMuSiC 2.1 ΔΔG for neutral GCK nonsynonymous substitutions reached 0.58 kcal/mol (95% CI 0.35–0.78 kcal/mol; Fig. 1g). When setting the threshold values to a median plus 2-times SD (ΔΔG = 1.42), 42% of MODY-associated nonsynonymous substitutions (but only two PHHI-associated nonsynonymous substitution) passed this threshold applied to the outcomes of PoPMuSiC 2.1. A similarly calculated threshold for SNAP2 was 6.5 (mean −58, 95% CI −77.06–−39.06), which allowed to classify 75% of the tested MODY-associated (and four PHHI-associated) nonsynonymous substitutions as disease-associated (Fig. 1h). In contrast, SIFT, PolyPhen-2, I-Mutant 3 and AlignGVGD [including Grantham variation (GV) or Grantham deviation (GD) alone] did not allow any improvement in the resolution based on increases in threshold values. Other tested methods, including PhD-SNP and SNPs&GO did not allow this adjustment because they generate only binary outcomes.

**Figure 2:**
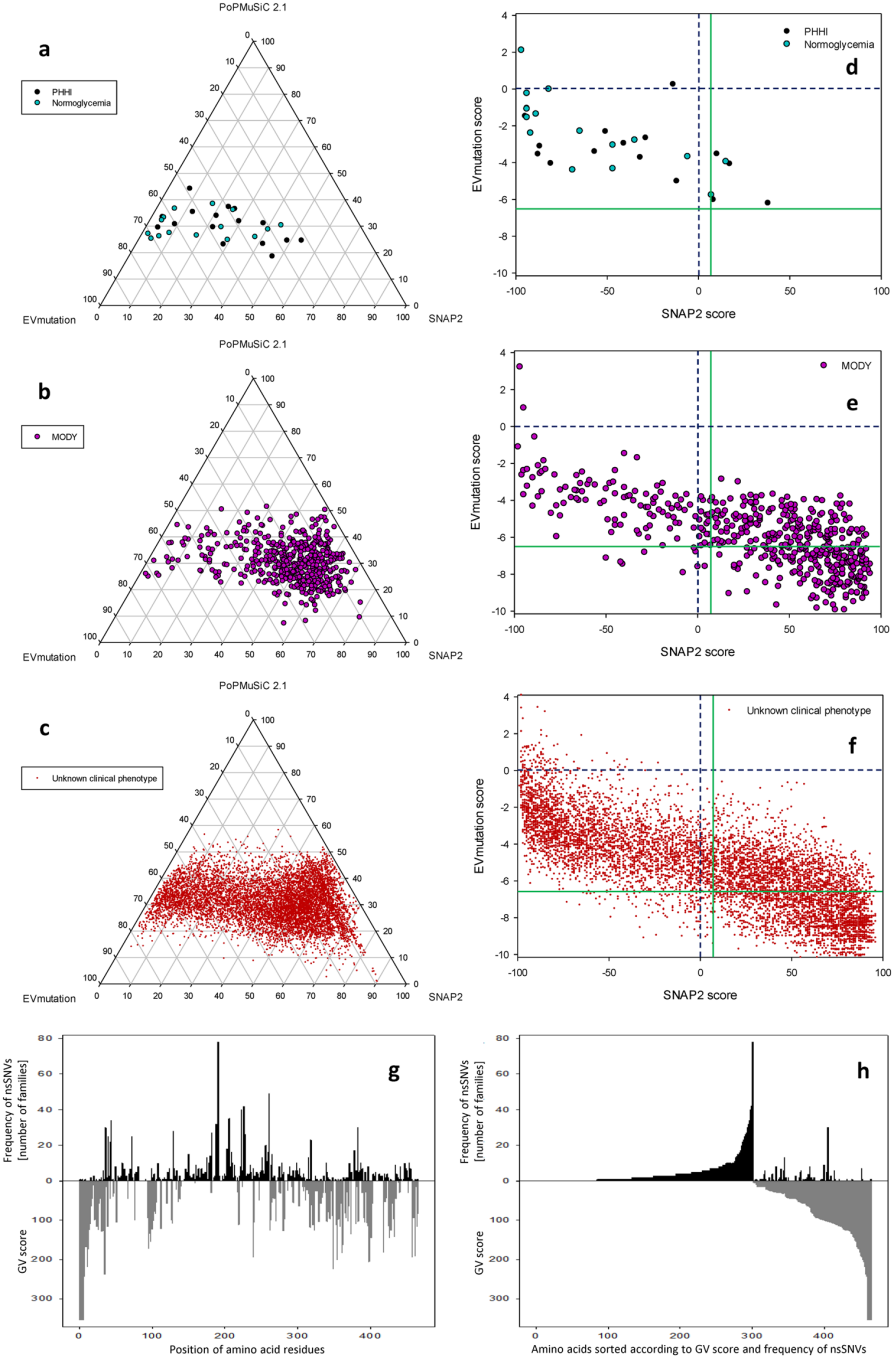
The combined analysis of the numerical scores of EVmutation, PoPMuSiC 2.1 and SNAP2 applied to GCK nonsynonymous substitutions with known clinical phenotype and of all putative GCK nonsynonymous substitutions and the frequency of disease-associated families. (**a**–**c**) Ternary plots of the EVmutation, PoPMuSiC 2.1 and SNAP2 scores. The numerical outcomes of each of the three prediction methods were transformed to the equal relative scale, and then ternary transformed in order to show the contributions of the predictors relative to each other, irrespectively on the absolute values of the predictions. (**d**–**f**) Scatter plots of numerical EVmutation and SNAP2 scores. The data are shown for GCK nonsynonymous substitutions associated with (**a**,**d**) PHHI and normoglycemia, (**b**,**e**) *GCK*-MODY, and (**c**,**f**) with unknown clinical phenotype (so far not observed in humans). (**g**,**h**) The comparison between the GV scores and the frequency of disease-associated families with nonsynonymous substitutions in GCK. The numbers of families affected by particular nonsynonymous substitutions are based on Osbak *et al*.^4^ (data known until 2009) and from a systematic review of the literature published in 2009–2017 and listed in the Web of Science database and/or mentioned in the HGMD database [n = 465 residues, of that 279 residues were disease-associated (1596 disease-associated families) and 164 residues were not evolutionarily conserved; for raw data, cf. Table S1]. Generally, areas with low GV values (GV < 61.3), which suggest high conservation, correspond to areas with frequently mutated residues except for S383. The data were sorted according to (**g**) the position of amino acid residues or (**h**) GV score. The pre-set (original) thresholds are highlighted with dashed dark-blue lines and newly defined evidence-based thresholds are highlighted with solid green lines (**d**–**f**).

We employed the evidence-based thresholds in the estimation of the theoretical frequency of putative MODY-associated variations among total hypothetic GCK nonsynonymous substitutions (Table 3). The distribution of resulting scores of EVmutation (Fig. 1i) and PoPMuSiC 2.1 (Fig. 1j) overlapped for total putative nonsynonymous substitutions and MODY-associated nonsynonymous substitutions, with slightly less MODY-associated nonsynonymous substitutions being associated with low EVmutation scores (Fig. 1i). In contrast, the total and MODY-associated SNAP2 scores did not have the same distribution, and more total nonsynonymous substitutions were associated with high SNAP2 scores (Fig. 1k). The three scores were only incompletely correlated. We found the strongest correlation between the SNAP2 and EVmutation scores (Pearson −0.778, *p* << 0.001; Spearman 0.789, *p* << 0.001, n = 8,189 nonsynonymous substitutions), followed by PoPMuSiC 2.1 and EVmutation (Pearson −0.406, *p* << 0.001; Spearman 0.383, *p* << 0.001, n = 8,493 nonsynonymous substitutions) and SNAP2 and PoPMuSiC 2.1 (Pearson −0.362, *p* << 0.001; Spearman 0.339, *p* << 0.001, n = 8,189 nonsynonymous substitutions). When we combined the three prediction methods, the ternary transformed data allowed to distinguish the nonsynonymous substitutions associated with PHHI or normoglycemia (Fig. 2a) from those associated with MODY (Fig. 2b). The nonsynonymous substitutions with unknown clinical phenotype (so far not observed in humans) followed the same distribution pattern; most of them accumulated in the region of high SNAP2 score and low EVmutation score (Fig. 2c). Raw EVmutation and SNAP2 scores were able to differentiate nonsynonymous substitutions associated with PHHI or normoglycemia (Fig. 2d) from those associated with MODY (Fig. 2f). The distribution pattern of nonsynonymous substitutions, which were so far not observed in humans, suggests the existence of two dominant phenotypes, one considered benign (EVmutation score from −2 to −4 and SNAP2 score <−50) and the other one predicted to be highly pathogenic and surprisingly underrepresented even among MODY patients (EVmutation score <−7.5 and SNAP2 score >70) (Fig. 2f).

**Table 3:** The estimations of the effects of total hypothetic GCK nonsynonymous substitutions.

Method: Variable	SNAP2	PoPMuSiC 2.1	EVmutation
Number of GCK variations analyzed	8,837	8,856	8,191
Mean ± SE	4.54 ± 0.63	1.10 ± 0.01	−5.35 ± 0.03
Min	−99	−1.88	−10.15
Max	96	5.77	4.10
Median	13	0.85	−5.36
25^th^ percentile	−52	0.32	−7.06
75^th^ percentile	58	1.70	−3.79

Three prediction methods, SNAP2, PoPMuSiC 2.1 and EVmutation, allowed differentiating at least in part between the neutral and MODY-associated nonsynonymous substitutions when considering their numerical outcomes. Thus, for these three methods, we computed (SNAP2 and PoPMuSiC 2.1) or retrieved (EVmutation) predictions for all possible amino acid exchanges within the GCK molecule, irrespectively on whether they are already known from humans or not.

### Comparison between the evolutionary conservation and frequency of nonsynonymous substitutions

We found a total of 301 invariant residues (65%) out of the total 465 residues of GCK when comparing GCK protein sequences from 12 vertebrate species (Fig. S2). The large and fully evolutionarily conserved regions particularly included the residue positions: 52–66, 77–93, 140–158, 160–180, 182–217, 225–238, 248–261, 404–417 and 441–451. All known glucose binding (T168, K169, N204, D205, E256 and E290), ATP binding (T228, T332, S336, V412 and L415) and allosteric (R63, Y215, M210, Y214, V452 and V455) sites of GCK^12, 13^ were fully evolutionarily conserved except for T332 (GV = 57.75) and V452 (GV = 29.61), which also displayed a high conservation (defined as GV < 61.3) (Table S1).

When plotted against the frequency of 1596 disease-associated families with nonsynonymous substitutions in GCK, all but one of the sites mutated in 15 or more families with MODY or PHHI (n = 23 amino acids) were considered as highly conserved, with zero GV score (Fig. 2g,h). The only exception was S383, which was repeatedly reported to be mutated to leucine or, less frequently, threonine in multiple European and Canadian families^4^ (Table S1). There is an overall correlation between positions that are mutated at a high frequency and sites that have a low GV (Pearson −0.182, *p* << 0.001; Spearman −0.394, *p* << 0.001; Fig. 2g,h). This correlation thus indicates that the nonsynonymous substitutions at highly conserved positions are strongly contributing to the disease manifestation, whereas the others may escape attention because they may not be associated with any phenotypes. Not all conserved residues were frequently mutated; for example, there were no nonsynonymous substitutions associated with the evolutionarily conserved residues 83–90. However, the extremely low frequency of disease-associated nonsynonymous substitutions was in exon 1 (any of its three forms), which corresponds to high GV values associated with the N-terminus of GCK (Fig. 2g,h).

## Discussion

**Figure 3:**
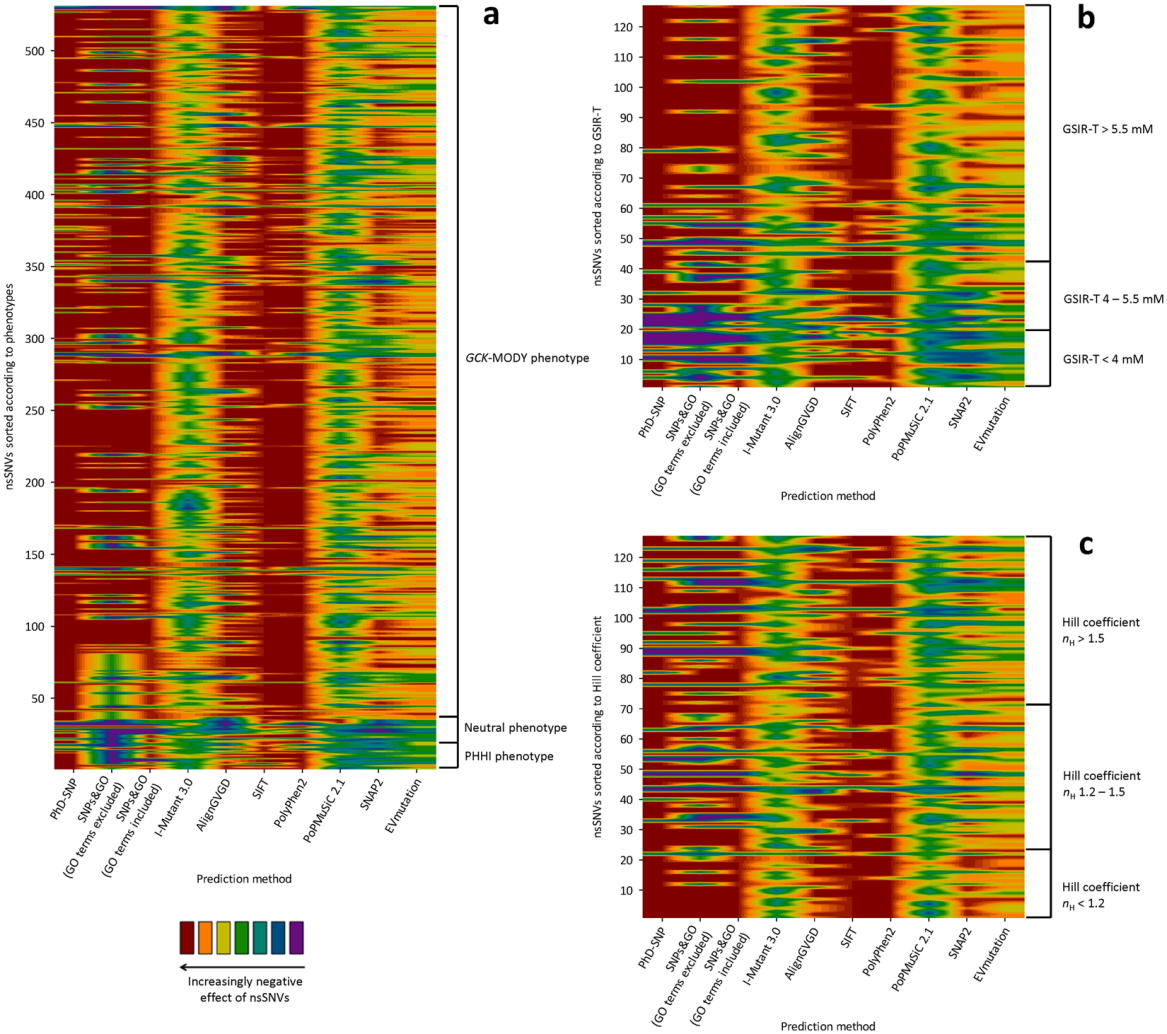
The prediction scores for nonsynonymous substitutions (nsSNVs) in the GCK amino acid sequence as plotted against clinical phenotypes (**a**), the GSIR-T (**b**) or the Hill coefficient (**c**). In the heatmaps, the nonsynonymous substitutions were sorted according to clinical phenotypes (**a**), the GSIR-T (**b**) or the Hill coefficient (**c**) and according to their position in the GCK molecule. The continuous scores were divided into seven categories according to computed effects of nsSNVs; the binary and ternary scores were depicted in colors of the two most extreme categories (red and violet); in case of ternary scores, the middle category was assigned the green color.

The use of prediction algorithms seems to be necessary part of science and diagnostics, especially in time of next-generation sequencing and other omics studies, for which the complete functional analyses of protein variations found are unfeasible and ineffective. Widely used databases list the outcomes of some of these algorithms; for example, the Ensembl genome browser provides the predictions generated by the SIFT and PolyPhen algorithms for each of the nonsynonymous substitutions listed^14^. This is convenient because the use of these algorithms minimizes the amount of nonsynonymous substitutions studied only to those predicted to be deleterious and thus potentially causing the phenotype in the respective study subject^15, 16^. The two predictors implemented in the Ensembl genome browser are also widely used in studies focusing on particular proteins, including those that focus on the activity of GCK. Some of these studies generated data, which are in agreement with the two predictors^10^, but accumulating evidence suggests that they may exhibit surprisingly high false positive rates (e.g., 29% and 43%, respectively, as reported by Romeo *et al*.^17^) and surprisingly low rates of correct predictions (53 and 63%, respectively^17^). Therefore, their outcomes may be over-interpreted when used without matching the data with the measurements of enzyme kinetics and clinical data. The field of prediction methods is developing quickly, with the EVmutation method being the latest important contribution to the field^11^. The EVmutation method reflects the epistasis by explicitly modeling interactions between all the pairs of residues in proteins, and was claimed to outperform dramatically the nowadays broadly used SIFT and PolyPhen methods^11^. However, here we have shown that EVmutation is associated with similar issues of poor sensitivity for activating and neutral nonsynonymous substitutions as are the previously developed models, despite the sensitivity of EVmutation for deactivating nonsynonymous substitutions was similar as in other prediction methods with the best performance (Table 2). The use of evidence-based thresholds is necessary in order to avoid low selectivity (Figs 1–2). The evidence-based adjustment of the thresholds allowed confident identification of up to three quarters of MODY-associated nonsynonymous substitutions, but all the methods failed to identify selectively the nonsynonymous substitutions associated with normoglycemia or hypoglycemia. The latter two groups largely overlapped in the outcomes of all the tested prediction methods and they were also interspersed with a minority of MODY-associated variations (which, however, dominate the datasets, and thus blur any analyses of nonsynonymous substitutions associated with normoglycemia or hypoglycemia; Table 2, Figs 1–3).

When focusing on MODY-associated genes, the most authoritative work was published by Flanagan *et al*.^18^, who tested 66 gain-of-function and 67 loss-of-function nonsynonymous substitutions in GCK, ABCC8 and KCNJ11. They concluded that the sensitivity of SIFT and PolyPhen reached 69% and 68%, but the specificity was only 13% and 16%, respectively, with both predictors predicting more precisely the loss-of-function nonsynonymous substitutions. In another study, Rees *et al*.^19^ found that SIFT and PolyPhen failed to correctly predict three out of 15 nonsynonymous substitutions in GKRP. False predictions of the benign phenotype in GKRP by PolyPhen were also noticed by Johansen *et al*.^20^. When focusing on GCK, most of the previous studies, which focused on MODY-associated nonsynonymous substitutions, noticed overall concordance between disease-associated phenotypes and predictions of deleterious effects based on SIFT and PolyPhen^10, 21–24^. This conclusion is in agreement with the present study, which found that these two methods tend to correctly identify MODY-associated nonsynonymous substitutions but also identify a large part of neutral nonsynonymous substitutions as deleterious and fail to correctly distinguish hypoglycemia-associated nonsynonymous substitutions (Table 2, Fig. 3).

In addition to the prediction analysis, in the present study, we provided basic kinetic characterization of 19 nonsynonymous substitutions in GCK. In agreement with previous studies^25, 26^, the dataset of MODY-associated nonsynonymous substitutions included some of the nonsynonymous substitutions, which paradoxically had near-normal kinetics. These included R250C with a GSIR-T of 5.0 mM and C434Y with a GSIR-T of 5.1 mM (Table 1). The C434Y affects one of the experimentally confirmed nitrosylation sites within the GCK molecule^27^. Although the function of C434 nitrosylation is unknown (in contrast with the modification of C371), the association of this nonsynonymous substitution with four independent Czech families^28^ clearly suggests its role in MODY onset and progression. Additionally, R250C is associated with a strong phenotype with manifestation during childhood and with confirmed family history^29^, and it is known in MODY patients of Serbian and Czech origin^29, 30^. All prediction algorithms suggest its deleterious effect (Table S2). Thus, our data illustrated that the MODY-associated nonsynonymous substitutions employ various mechanisms of action. Of particular interest is the decreased sensitivity of F150L to the regulation via GlcNAc. This glucose derivative is known to inhibit glucokinase competitively in a cooperative manner^31^. It is a natural part of biopolymers on the surface of many pathogens and natural compounds in our food. Another hexokinase isoform was already confirmed to serve as a sensor for the detection of bacterial GlcNAc^32^; thus, glucokinase can also serve as a pattern recognition receptor, and pathogen- and food-derived GlcNAc may differentially affect GCK action in health and disease. Such regulation would be impaired in *GCK*-MODY patients heterozygous for F150L. This speculation requires further verification in the near future.

In conclusion, this study provided the first robust evidence for choosing the best-fit method and the evidence-based threshold to predict the effects of GCK nonsynonymous substitutions for which *in vitro* data are still absent. Even with the newly proposed evidence-based thresholds, the precision of the available methods allowed predicting correctly up to 75% of true MODY-associated variations, leaving the remaining quarter of true MODY-associated nonsynonymous substitutions in the grey zone of uncertain predictions. The combined computational analysis of total hypothetical GCK nonsynonymous substitutions identified a group of putatively highly pathogenic variations (EVmutation score <−7.5 and SNAP2 score >70), which were surprisingly underrepresented among MODY patients. We speculate that a negative selection may play a role in the low frequency of variations predicted to be highly pathogenic despite the heterozygous manifestation of inactivating *GCK* variations is generally associated with only a relatively mild disease phenotype.

We reported here a novel approach how to interpret the outcomes of prediction methods. The tailor-made thresholds based on non-phenotypic variations can be used to improve predictions that reflect genuine effects. On a model of GCK, the combination of EVmutation and SNAP2 with implemented evidence-based thresholds seems to be readily applicable and feasible for the analyses of any newly found GCK variations. Further research should elucidate, whether the same approach can be generalized and applied across the proteome. Evidence-based cross-correlations of new bioinformatics methods with available experimental and clinical data represent the most promising approach that allows analyzing newly found amino acid sequence variations without time-consuming experiments, especially when multiple kinds of measurements would be needed in order to characterize the effect of a particular amino acid sequence variation.

## Methods

We prepared a series of *GCK* constructs (Table S3) that carry previously published^24, 28, 30, 33–35^ nonsynonymous substitutions that are associated with the *GCK*-MODY phenotype in patients of Czech origin. Additionally, we prepared new constructs carrying nonsynonymous substitutions R63S, M251C and F260L for experimental purposes only. The constructs consisted of the wild-type (WT)-GCK isoform 1 in pGEX-5X-2 (kind gift from Dr. Navas^36^), to which we introduced the particular nonsynonymous substitutions via site-directed mutagenesis. We measured the glucokinase activity spectrophotometrically using a coupled reaction with glucose-6-phosphate dehydrogenase, and we identified the increasing concentrations of NADPH at 340 nm as described^37^. We computed the kinetic parameters, including the Hill coefficient *n*_H_, the turnover number *k*_cat_, *S*_0.5_, ATP *K*_M_, the protein stability, RAI and GSIR-T, as described^37–39^. For each construct, we also performed a competitive inhibition assay using GlcNAc and calculated its IC_50_ at 5 mM glucose and 5 mM ATP.

We employed nine prediction methods (for a detailed overview, cf. Suppl. Methods.) for the prediction of phenotypic effects of GCK nonsynonymous substitutions. These included methods that use evolution-based sequence information (SIFT, PhD-SNP), as well as those that take into account the chemical and physical characteristics of amino acids (Align-GVGD) or protein structural attributes combined with multiple sequence alignment-derived information (EVmutation, PolyPhen-2, SNAP2 and SNPs&GO). A single amino acid nonsynonymous substitution can result in notable change in the protein stability, which is represented by a change in its Gibbs free energy (∆∆G) upon folding. Therefore, we also employed two predictors that focused on the stability properties of nonsynonymous substitutions, I-Mutant 3.0 and PoPMuSiC 2.1. We performed these predictions for an up-to-date set of published *GCK*-MODY- and PHHI-associated nonsynonymous substitutions and for those, which are not associated with any monogenically inherited disease effects. In addition to referring to relevant publications, we retrieved data on the GCK nonsynonymous substitutions from the Ensembl, dsSNP, UniProtKB and HGMD databases. We matched the predictions with the previously published and newly generated experimental data on enzyme kinetics and with clinical data available from previously published studies on *GCK*-MODY, PHHI and PNDM. Data are shown as the means ± SE, unless stated otherwise.

### Data availability

A detailed overview of the nonsynonymous substitutions analyzed, including the outcomes of the prediction methods, previously published and newly generated experimental data on enzyme kinetics, available clinical data and relevant references are all listed in Table S2; detailed protocols are provided in Suppl. Methods.

## Electronic supplementary material

Supplementary Information
