# Supplementary material for: Evidence-based tailoring of bioinformatics approaches to optimize methods that predict the effects of nonsynonymous amino acid substitutions in glucokinase
Source: Sci Rep. 2017 Aug 25;7:9499. doi: 10.1038/s41598-017-09810-0 (PMC5573313; doi:10.1038/s41598-017-09810-0)
Supplement: Supplementary file 1 — Supplementary Information [file 41598_2017_9810_MOESM1_ESM.pdf]

SUPPLEMENTARY INFORMATION TO:

Evidence-based tailoring of bioinformatics approaches to optimize methods that predict the effects of nonsynonymous amino acid substitutions in glucokinase

Daniela Šimčíková[1], Lucie Kocková[1], Kateřina Vackářová[1], Miroslav Těšínský[1], Petr Heneberg[1,*]

Legends to supplementary materials

Fig. S1. The outcomes of prediction methods are only weakly correlated with clinical parameters measured in disease-affected patients. (a-d) Number of algorithms demonstrating effect of the nonsynonymous substitution as plotted against (a) fasting plasma glucose measured in PHHI (white circles), MODY (black circles) and PNDM (black triangles) patients, (b) OGTT glycemia at t = 120 min as measured in MODY patients, (c) HbA_{1c} measured in MODY (black circles) and PNDM (white circles) patients, (d) age at diagnosis of PHHI (white circles), MODY (black circles) and PNDM (black triangles). (e-g) EVmutation scores plotted against (e) fasting plasma glucose of MODY and PNDM patients, (f) HbA_{1c} of MODY and PNDM patients and (g) age at diagnosis of diabetes of MODY patients. Linear (a-d, f-g) and Gaussian (e) regression curves are shown.

Fig. S2. The alignment of amino acid sequences of GCK of 13 vertebrate species, which was used to calculate the Grantham variation (GV) scores. The alignment was obtained using the ClustalW algorithm, and includes the following sequences: Homo sapiens NP_000153.1; Mus musculus NP_034422.2; Rattus norvegicus XP_006251241; Bos taurus NP_001095772.1; Danio rerio NP_001038850; Cyprinus carpio ACD37722; Meleagris gallopavo XP_010725006; Aquila chrysaetos XP_011573674; Ficedula albicollis XP_005057963; Xenopus laevis NP_001079298; Nanorana parkeri XP_018422966; Anolis carolinensis XP_003224263; and Lepisosteus oculatus XP_006625388.

Table S1. Raw data used for the formation of Fig. 2. The table contains the numbers of families affected by the GCK nonsynonymous substitutions identified globally until year 2017, and these frequencies are matched with the GV values for each amino acid within the GCK molecule.

Table S2. The detailed overview of the GCK nonsynonymous substitutions known from humans and analyzed in the present study. The data are based on a thorough systematic review of previously published data and includes also data generated newly in course of the present study. The table includes the outcomes of prediction methods, previously published and newly generated experimental data on enzyme kinetics, available clinical data and relevant references.

Table S3. Nonsynonymous substitutions analyzed in vitro in this study and the corresponding primers used in site-directed mutagenesis.

Supplementary methods

Experimental procedures

We expressed glucokinase (GCK) from the expression vector, pGEX-5X-2, with an insert that encoded the wild-type GCK isoform 1; the expression vector was provided as a kind gift from Dr. Navas (Universidad Complutense de Madrid) [1]. We introduced variations into the expression construct via site-directed mutagenesis (QuikChange site-directed mutagenesis kit, Agilent Technologies, Santa Clara, CA); the list of primers used is provided as Suppl. Tab. S3. We verified all the constructs via bidirectional Sanger sequencing. Then, we prepared GCK and its mutant forms as fusion proteins with N-terminal glutathione-S-transferase in the Escherichia coli BL21 Gold(DE3) strain (Agilent Technologies, Santa Clara, CA). We grew the cells at 37°C to OD 0.7, and the expression was induced by adding IPTG to a final concentration of 0.2 mM. We incubated the cultures at 22°C for 16 hours with orbital shaking (240 rpm). Afterwards, we harvested the cells by centrifuging, and resuspended the pellets in a breaking buffer (25-fold smaller volume than the culture volume; PBS, pH 7.4 containing 4 mM $MgCl_2$, 1 mM PMSF, 5 mM DTT, 0.5% Triton X-100, lysozyme and DNase I) followed by 30 min of incubation at room temperature. We lysed the cells via mild sonication on ice. We centrifuged the lysates (4°C; 20,000× g) and immediately incubated the supernatants with Glutathione Sepharose (GE Healthcare Life Sciences, Chicago, IL). Subsequently, we washed the beads twice and eluted GST-GCK with 50 mM Tris, 200 mM KCl, pH 8.0, containing 5 mM DTT and 10 mM glutathione. We performed the entire purification procedure at 4°C. We determined the protein concentration using a Bradford assay (Serva, Heidelberg, DE) with bovine serum albumin used as a standard.

We measured the GCK activity spectrophotometrically using a coupled reaction with glucose-6-phosphate dehydrogenase (Sigma-Aldrich, St. Louis, MO); the increasing concentration of NADPH was determined at 340 nm, as described previously [2-3]. One unit (U) of GCK was defined as the amount of enzyme that phosphorylated 1 μmol of glucose per min at 30°C under assay conditions. In the case of glucose as the variable substrate (0–200 mM), we measured these assays using two concentrations of ATP – 0.5 mM and 5 mM; the GCK activity exhibited a sigmoidal dependency, which satisfied the Hill equation. However, the GCK activity with variable ATP concentrations (0–5 mM) followed hyperbolic Michaelis-Menten kinetics; we performed these assays at two glucose concentrations: at the corresponding $S_{0.5}$ and 50 mM. We performed the competitive inhibition with N-acetylglucosamine (GlcNAc) at 5 mM glucose and 5 mM ATP under identical assay conditions.

We determined the temperature stability at 30°C in the time course of 100 min at 50 mM glucose and 5 mM ATP. Protein concentrations varied over separate preparations (30–300 μg/mL) without having an effect on the protein stability.

We calculated, based on the determined kinetic variables ($S_{0.5}$, n_H, k_{cat} and ATP K_M), the relative activity index (RAI) and the glucose threshold for glucose-stimulated insulin release (GSIR-T). The RAI values serve as a direct comparison of the GCK mutants with the wild-type enzyme. The equation has been previously published [4]. We employed a minimal mathematical model, which reflects the kinetic characteristics of the wild-type GCK and its mutant forms, as well as

the stability coefficient and adaptation through the expression coefficient to predict the β-cell threshold for GSIR. The previously published consensual assumptions were fulfilled [2,5].

Prediction methods

For the prediction analyses mentioned below, we used a protein identifier (GCK NCBI code: NP_000153.1; GCK Swiss-Prot code: P35557), or directly an amino acid sequence in FASTA format.

We retrieved data related to the nonsynonymous single nucleotide variations (nonsynonymous substitutions, abbreviated as nsSNVs) in the expressed region of the GCK gene from the Ensembl [6], dbSNP [7], UniProtKB [8] and HGMD [9] databases and from a systematic review of the literature published in 2009-2017 and listed in the Web of Science database [10]. We obtained the structure of the closed form of GCK (PDB ID: 1V4S [11]) from the Protein Data Bank (PDB) [12].

In this study, we employed methods that use evolution-based sequence information (SIFT, PhD-SNP) and methods that take into account the chemical and physical characteristics of amino acids (Align-GVGD) or protein structural attributes combined with multiple sequence alignment-derived information (PolyPhen-2, SNAP2, SNPs&GO) to predict the phenotypic effect of nonsynonymous substitutions. A single amino acid substitution can result in a notable change in the protein stability, which is represented by a change in its Gibbs free energy (ΔΔG) upon folding. Therefore, we employed two predictors that focus on the stability properties of the nonsynonymous substitutions, I-Mutant 3.0 and PoPMuSiC 2.1. We also used the recently published prediction method, EVmutation, to compare the existing approaches and its new epistatic approach for protein function and the stability prediction.

The Sorting Intolerant From Tolerant (SIFT) method [13] is based on the hypothesis that protein evolution is correlated with protein function. Functionally relevant amino acids should be conserved in the protein family, whereas less important positions should be diverse. The SIFT Human Protein predicts whether nonsynonymous substitutions affect the protein function for all Ensembl transcripts with an assigned ENSP number (GCK ENSP: ENSP00000384247). Based on their scores, the substitutions are considered to be damaging (≤0.05) or tolerated (>0.05), ideally with median sequence information (also referred as the median conservation value) between 2.75 and 3.25. The median sequence information provides an assessment of the confidence, and SIFT computes the conservation value at each position in the alignment. The conservation value ranges from 0, which means that all 20 amino acids are at that position, to 4.32, which means that the position is completely conserved. A sufficient diversity within the aligned sequences is maintained by median sequence information of ~3.0.

The PolyPhen-2 (Polymorphism Phenotyping v2) method [14] estimates the probability of the nonsynonymous substitution to adversely affect protein function based on sequence, phylogenetic and structural features. The nonsynonymous substitution is predicted as probably damaging (0.85–1.00), possibly damaging (0.15–0.84) or benign (<0.15). We identified the nonsynonymous substitution effect according to the HumDiv score. The model was trained on a

dataset that involved known effects of damaging alleles that cause human Mendelian diseases that are annotated in the UniProtKB database.

SNAP2 (Screening for non-acceptable polymorphisms) [15] is a neural network-based classifier that predicts the impact of nonsynonymous substitutions based on evolutionary information, structural features and solvent accessibility. The score ranges from -100 (strong neutral prediction) to +100 (strong effect prediction).

PhD-SNP (Predictor of human Deleterious Single Nucleotide Polymorphisms) [16] is a support vector machine (SVM)-based classifier that distinguishes disease-related nonsynonymous substitutions from neutral ones by reflecting the nature of the substitution and properties of the neighboring sequence environment. The method was optimized using a dataset of neutral and deleterious variations taken from the UniProtKB/Swiss-Prot.

The SNPs&GO method [17] is based on a principle very similar to PhD-SNP. In contrast to PhD-SNP, the SNPs&GO also takes into account protein function information that is defined by Gene Ontology (GO) [18-19] terms. GO terms are directly retrieved only if a Swiss-Prot code is used. If GO terms are not included (only protein sequence input), the accuracy of the method is thought to be lower and comparable with PhD-SNP [17].

Align-GVGD [20-21] classifies the amino acid substitutions and their functional effect according to the "C-score" (from C0 – neutral to C65 – deleterious), which is based on the cross-species protein multiple sequence alignment with a comparison of the physical and chemical characteristics of amino acids. The Align-GVGD combines the GV (Grantham variation) and GD (Grantham deviation) score. We expressed the evolutionary conservation of the amino acid sequence of the pancreatic isoform of GCK in the form of a GV score, which was based on the alignment of the human GCK protein sequence with the GCK sequences of 12 other vertebrate species. Because the GCK sequence is highly conserved, the alignment included not only mammals (three species) but also birds, amphibians, reptiles and fish (Suppl. Fig. S2). To calculate the GV score, we used the multiple sequence alignment, which was formed using ClustalW in MEGA6, and built on the following sequences: Homo sapiens NP_000153.1; Mus musculus NP_034422.2; Rattus norvegicus XP_006251241; Bos taurus NP_001095772.1; Danio rerio NP_001038850; Cyprinus carpio ACD37722; Meleagris gallopavo XP_010725006; Aquila chrysaetos XP_011573674; Ficedula albicollis XP_005057963; Xenopus laevis NP_001079298; Nanorana parkeri XP_018422966; Anolis carolinensis XP_003224263; Lepisosteus oculatus XP_006625388. Positions with zero GV score have the same amino acids across all species and are thus invariant, whereas the GV increases when the alignment demonstrates evidence for variation in the particular residue. The GCK gene does not have any insertions or deletions of amino acids in the studied species, except for the N- and C-terminal parts of the molecule; thus, nearly all the variability was assigned to nonsynonymous substitutions.

I-Mutant 3.0 [22] was designed to estimate the protein stability change caused by nonsynonymous substitutions. The tool was trained on a dataset built on the information from ProTherm [23], which is a comprehensive thermodynamic database of experimental data for wild-type and mutant proteins. Based on the protein structure or the sequence, the difference ($\Delta\Delta G$ value) between the unfolding Gibbs free energies of the mutated and wild-type protein is calculated. In the present study, we based the $\Delta\Delta G$ values on the protein structure of GCK (PDB ID: 1V4S [11]). Nonsynonymous substitutions with $\Delta\Delta G > 0.5$ kcal/mol are considered to be

largely stabilizing, and those with ΔΔG<-0.5 kcal/mol are predicted as largely destabilizing. Other nonsynonymous substitutions with ΔΔG in the range from -0.5 to 0.5 kcal/mol have a weak effect.

Another web server that allows predicting the thermodynamic stability changes upon the nonsynonymous substitution is PoPMuSiC-2.1 [24]. This method reflects the solvent accessibility of the mutated residue. The predictions are derived from the structure of the target protein (GCK PDB ID:1V4S). The ΔΔG values lower than 0 kcal/mol are assigned to stabilizing nonsynonymous substitutions, and those that are higher than 0 kcal/mol are assigned to destabilizing nonsynonymous substitutions.

The recently reported prediction method, EVmutation [25], exploits the epistatic approach. Thus, it takes into account explicitly modelling of interactions between all the pairs of residues in the proteins and bases in RNAs to predict nonsynonymous substitution effects. Within validation, EVmutation predictions were compared with outcomes from 34 high-throughput mutagenesis experiments. The EVmutation scores (ΔE) below 0 are assigned to deleterious nonsynonymous substitutions, values above 0 correspond to beneficial nonsynonymous substitutions, and values equal to 0 correspond to neutral nonsynonymous substitutions.

The developers of all prediction methods suggested interpreting the resulting predictions using arbitrary scores as threshold values. We presented the calculations using these arbitrarily suggested interpretations and thresholds in Table 2. However, arbitrary thresholds were associated with extreme uncertainty and overestimated the effects of neutral nonsynonymous substitutions. Nevertheless, we found that three prediction methods, PolyPhen-2, SNAP2 and EVmutation, allowed differentiating at least in part between the neutral and MODY-associated nonsynonymous substitutions when considering their numerical outcomes. Thus, for these three methods, we computed (PolyPhen-2 and SNAP2) or retrieved (EVmutation) predictions for all possible amino acid exchanges within the GCK molecule, irrespectively on whether they are already known from humans or not. For SNAP2, we retrieved 8,837 predictions with mean value 4.54±0.63 (min -99, max 96, median 13, 25th percentile -52, 75th percentile 58). For PoPMuSiC 2.1, we retrieved 8,856 predictions with mean value 1.10±0.01 (min –1.88, max 5.77, median 0.85, 25th percentile 0.32, 75th percentile 1.70). For EVmutation, we retrieved 8,191 predictions with mean value -5.35±0.03 (min –10.15, max 4.10, median -5.36, 25th percentile -7.06, 75th percentile -3.79). We applied two types of adjusted thresholds in order to be able to predict nonsynonymous substitutions, which are likely to serve as causative MODY nonsynonymous substitutions, and which are likely to be benign or activating. We calculated the thresholds by computing the medians and SDs of scores for nonsynonymous substitutions, which do not cause any monogenically inherited disease. We calculated the threshold for predicting the MODY-associated nonsynonymous substitutions as median of scores for nonsynonymous substitutions, which do not cause any monogenically inherited disease, with the addition of 2 SDs. We calculated the threshold for predicting the benign (or activating) nonsynonymous substitutions as the median value of scores for nonsynonymous substitutions, which do not cause any monogenically inherited disease. We used these evidence-based thresholds for a further validation of these methods.

We analyzed the data by one-way ANOVA with Tukey`s post-tests and computed the $S_{0.5}$, Hill coefficient n_H, k_{cat} and ATP K_M via non-linear regression analyses. We obtained IC_{50} using four parameters logistic curve fitting. Multiparametric analyses included the detrended

correspondence analyses. We calculated Pearson product moment correlation coefficients and Spearman rank order correlation coefficients in order to correlate the numerical outputs of EVmutation, PoPMuSiC 2.1 and SNAP2 prediction methods applied to total hypothetic GCK nonsynonymous substitutions for which the outcomes of all the three prediction methods were available ($n_{PoPMuSiC\ 2.1\ /\ EVmutation}$ = 8,493 nonsynonymous substitutions, $n_{SNAP2\ /\ PoPMuSiC\ 2.1\ and\ SNAP2\ /\ EVmutation}$ = 8,189 nonsynonymous substitutions). We also calculated the two correlation coefficients in order to compare GV with the frequency of families (n = 465 residues, of that 279 residues were disease-associated (1596 disease-associated families) and 164 residues were not evolutionarily conserved). Data were shown as means ± SE, unless stated otherwise. We performed the calculations and plotted the figures in PAST 2.14 and SigmaPlot 12.0.

Supplementary references for Supplementary Methods

1 García-Herrero CM, Galán M, Vincent O, et al. (2007) Functional analysis of human glucokinase gene mutations causing MODY2: exploring the regulatory mechanisms of glucokinase activity. Diabetologia 50:325–333

2 Davis EA, Cuesta-Muñoz A, Raoul M, et al. (1999) Mutants of glucokinase cause hypoglycaemia- and hyperglycaemia syndromes and their analysis illuminates fundamental quantitative concepts of glucose homeostasis. Diabetologia 42:1175–1186

3 Liang Y, Kesavan P, Wang L, et al. (1995) Variable effects of maturity-onset-diabetes-of-youth (MODY)-associated glucokinase mutations on substrate interactions and stability of the enzyme. Biochem J 309:167–173

4 Matschinsky FM (2009) Assessing the potential of glucokinase activators in diabetes therapy. Nat Rev Drug Discov 8:399–416

5 Matschinsky FM, Davis EA, Cuesta-Muñoz A, et al. (2000) The glucokinase system and the regulation of blood sugar. In: Matschinsky DM, Magnuson MA (eds) Molecular pathogenesis of MODYs. Karger, Basel, pp 99–108

6 Ensembl (2017) Ensembl genome browser 88; http://www.ensembl.org/; accessed 11 April 2017

7 NCBI (2017) NCBI dbSNP Build 150; https://www.ncbi.nlm.nih.gov/projects/SNP/index.html; accessed 11 April 2017

8 EMBL-EBI, SIB, PIR (2017) UniProtrelease 2017_04; http://www.uniprot.org; accessed 11 April 2017

9 Stenson PD, Mort M, Ball EV, et al. (2014) The Human Gene Mutation Database: building a comprehensive mutation repository for clinical and molecular genetics, diagnostic testing and personalized genomic medicine. Hum Genet 133:1–9

10 Thomson Reuters (2017) Web of Science. http://apps.webofknowledge.com; accessed 11 April 2017

11 Kamata K, Mitsuya M, Nishimura T, Eiki J, Nagata Y (2004) Structural basis for allosteric regulation of the monomeric allosteric enzyme human glucokinase. Structure 12:429–438

12 RSCB (2017) PDB-101. http://www.rcsb.org/pdb/home/home.do; accessed 11 April 2017

13 Kumar P, Henikoff S, Ng PC (2009) Predicting the effects of coding non-synonymous variants on protein function using the SIFT algorithm. Nat Protoc 4:1073–1081

14 Adzhubei IA, Schmidt S, Peshkin L, et al. (2010) A method and server for predicting damaging missense mutations. Nat Methods 7:248–249

15 Hecht M, Bromberg Y, Rost B (2015) Better prediction of functional effects for sequence variants. BMC Genomics 16(Suppl 8):S1–S12

16 Capriotti E, Calabrese R, Casadio R (2006) Predicting the insurgence of human genetic diseases associated to single point protein mutations with support vector machines and evolutionary information. Bioinformatics 22:2729–2734

17 Calabrese R, Capriotti E, Fariselli P, Martelli PL, Casadio R (2009) Functional annotations improve the predictive score of human disease-related mutations in proteins. Hum Mutat 30:1237–1244

18 Ashburner M, Ball CA, Blake JA, et al. (2000) Gene Ontology: tool for the unification of biology. Nat Genet 25:25–29

19 The Gene Ontology Consortium (2015) Gene Ontology Consortium: going forward. Nucleic Acids Res 43:D1049–D1056

20 Tavtigian SV, Deffenbaugh AM, Yin L, et al. (2005) Comprehensive statistical study of 452 BRCA1 missense substitutions with classification of eight recurrent substitutions as neutral. J Med Genet 43:295–305

21 Mathe E, Olivier M, Kato S, Ishioka C, Hainaut P, Tavtigian SV (2006) Computional approaches for predicting the biological effect of p53 missense mutations: a comparison of three sequence analysis based methods. Nucleic Acids Res 34:1317–1325

22 Capriotti E, Fariselli P, Rossi I, Casadio R (2008) A three-state prediction of single point mutations on protein stability changes. BMC Bioinformatics 9(Suppl 2):S6–S14

23 Kumar MD, Bava KA, Gromiha MM, et al. (2006) ProTherm and ProNIT: thermodynamic databases for proteins and protein-nucleic acid interactions. Nucleic Acids Res 34:D204–D206

24 Dehouck Y, Kwasigroch JM, Gilis D, Rooman M (2011) PoPMuSiC 2.1: a web server for the estimation of protein stability changes upon mutation and sequence optimality. BMC Bioinformatics 12:151–162

25 Hopf TA, Ingraham JB, Poelwijk FJ, et al. (2017) Mutation effects predicted from sequence co-variation. Nat Biotechnol 33:128–135

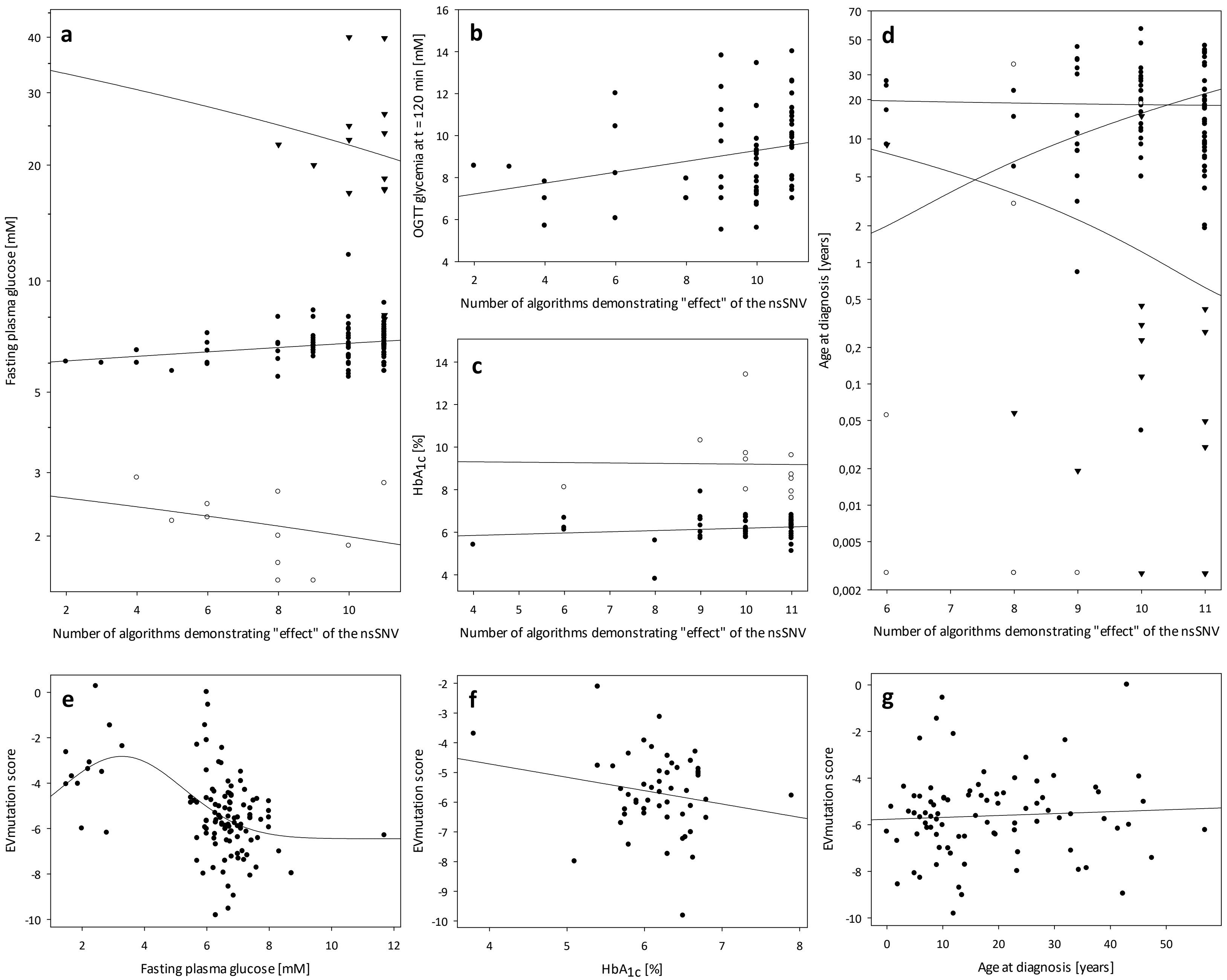

a
b
c
d
e
f
g
Fasting plasma glucose [mM]
OGTT glycemia at t = 120 min [mM]
HbA1c [%]
Age at diagnosis [years]
Number of algorithms demonstrating "effect" of the nsSNV
EVmutation score

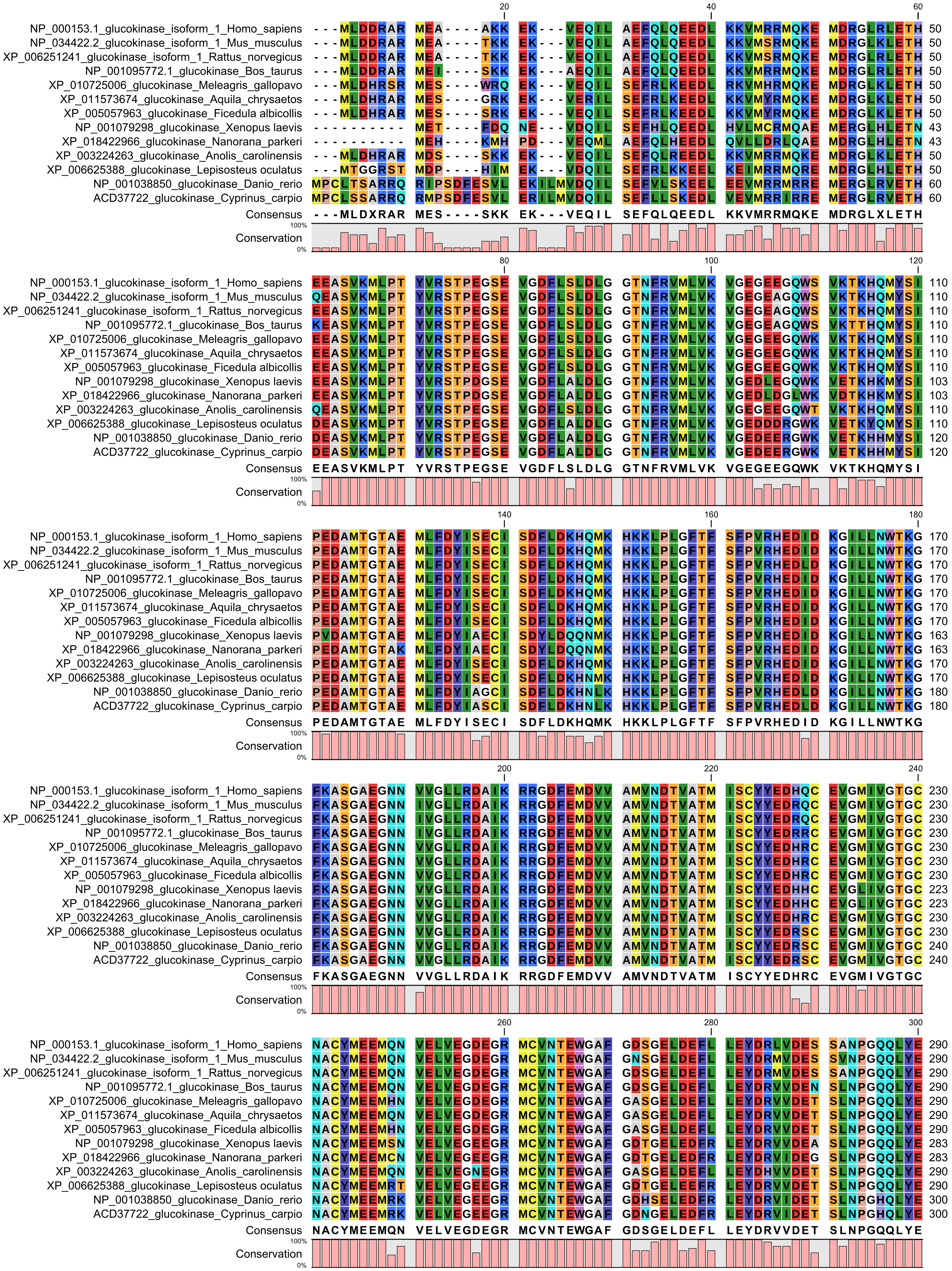

20 40 60
NP_000153.1_glucokinase_isoform_1_Homo_sapiens ---MLDDRAR MEA----AKK EK---VEQIL AEFQLQEEDL KKVMRRMQKE MDRGLRLETH 50
NP_034422.2_glucokinase_isoform_1_Mus_musculus ---MLDDRAR MEA----TKK EK---VEQIL AEFQLQEEDL KKVMSRMQKE MDRGLKLETH 50
XP_006251241_glucokinase_isoform_1_Rattus_norvegicus ---MLDDRAR MEA----TKK EK---VEQIL AEFQLQEEDL KKVMSRMQKE MDRGLKLETH 50
NP_001095772.1_glucokinase_Bos_taurus ---MLDDRAR MEI----SKK EK---AEQIL AEFQLQEEDL KKVMRRMQKE MDRGLRLETH 50
XP_010725006_glucokinase_Meleagris_gallopavo ---MLDHRSR MES----WRQ EK---VEQIL SEFRLKEEDL RKVMHRMQKE MDRGLKLETH 50
XP_011573674_glucokinase_Aquila_chrysaetos ---MLDHRAR MES----GRK EK---VERIL SEFRLKEEDL KKVMYRMQKE MDRGLKLETH 50
XP_005057963_glucokinase_Ficedula albicollis ---MLDHRAR MES----SRK EK---VEQIL SEFRLKEEDL KKVMYRMQKE MDRGLKLETH 50
NP_001079298_glucokinase_Xenopus laevis ---------- MET----FDQ NE---VDQIL SEFHLQEEDL HVLMCRMQAE MERGLHLETN 43
XP_018422966_glucokinase_Nanorana_parkeri ---------- MEH----KMH PD---VEQML AEFQLHEEDL QVLLDRLQAE MDRGLHLETN 43
XP_003224263_glucokinase_Anolis_carolinensis ---MLDHRAR MDS----SKK EK---VEQIL SEFQLREEDL KKVMRRMQKE MDRGLKLETH 50
XP_006625388_glucokinase_Lepisosteus oculatus ---MTGGRST MDP----HIM EK---VDQIL SEFRLKKEEL KEIMRRMQRE MDRGLHLETH 50
NP_001038850_glucokinase_Danio_rerio MPCLTSARRQ RIPSDFESVL EKILMVDQIL SEFVLSKEEL EEVMRRMRRE MERGLRVETH 60
ACD37722_glucokinase_Cyprinus_carpio MPCLSSARRQ RMPSDFESVL ERILMVDQIL SEFLLSKEDL VEVMRRIRRE MERGLRVETH 60
Consensus ---MLDXRAR MES----SKK EK---VEQIL SEFQLQEEDL KKVMRRMQKE MDRGLXLETH
100%
Conservation
0%
80 100 120
NP_000153.1_glucokinase_isoform_1_Homo_sapiens EEASVKMLPT YVRSTPEGSE VGDFLSLDLG GTNFRVMLVK VGEGEEGQWS VKTKHQMYSI 110
NP_034422.2_glucokinase_isoform_1_Mus_musculus QEASVKMLPT YVRSTPEGSE VGDFLSLDLG GTNFRVMLVK VGEGEAGQWS VKTKHQMYSI 110
XP_006251241_glucokinase_isoform_1_Rattus_norvegicus EEASVKMLPT YVRSTPEGSE VGDFLSLDLG GTNFRVMLVK VGEGEAGQWS VKTKHQMYSI 110
NP_001095772.1_glucokinase_Bos_taurus KEASVKMLPT YVRSTPEGSE VGDFLSLDLG GTNFRVMLVK VGEGEAGQWS VKTTHQMYSI 110
XP_010725006_glucokinase_Meleagris_gallopavo EEASVKMLPT YVRSTPEGSE VGDFLSLDLG GTNFRVMLVK VGEGEEGQWK VKTKHQMYSI 110
XP_011573674_glucokinase_Aquila_chrysaetos EEASVKMLPT YVRSTPEGSE VGDFLSLDLG GTNFRVMLVK VGEGEEGQWK VKTKHQMYSI 110
XP_005057963_glucokinase_Ficedula albicollis EEASVKMLPT YVRSTPEGSE VGDFLSLDLG GTNFRVMLVK VGEGEEGQWK VKTKHQMYSI 110
NP_001079298_glucokinase_Xenopus laevis EEASVKMLPT YVRSTPDGSE VGDFLALDLG GTNFRVMLVK VGEDLEGQWK VETKHKMYSI 103
XP_018422966_glucokinase_Nanorana_parkeri EEASVKMLPT YVRSTPDGSE VGDFLALDLG GTNFRVMLVK VGEDLDGLWK VDTKHKMYSI 103
XP_003224263_glucokinase_Anolis_carolinensis QEASVKMLPT YVRSTPEGSE VGDFLSLDLG GTNFRVMLVK VGEGEEGQWT VKTKHQMYSI 110
XP_006625388_glucokinase_Lepisosteus oculatus DEASVKMLPT YVRSTPEGSE VGDFLALDLG GTNFRVMLVK VGEDDDRGWK VETKYQMYSI 110
NP_001038850_glucokinase_Danio_rerio DEASVKMLPT YVRSTPEGSE VGDFLALDLG GTNFRVMLVK VGEDEERGWK VETKHHMYSI 120
ACD37722_glucokinase_Cyprinus_carpio DEASVKMLPT YVRSTPEGSE VGDFLALDLG GTNFRVMLVK VGEDEERGWK VETKHHMYSI 120
Consensus EEASVKMLPT YVRSTPEGSE VGDFLSLDLG GTNFRVMLVK VGEGEEGQWK VKTKHQMYSI
100%
Conservation
0%
140 160 180
NP_000153.1_glucokinase_isoform_1_Homo_sapiens PEDAMTGTAE MLFDYISECI SDFLDKHQMK HKKLPLGFTF SFPVRHEDID KGILLNWTKG 170
NP_034422.2_glucokinase_isoform_1_Mus_musculus PEDAMTGTAE MLFDYISECI SDFLDKHQMK HKKLPLGFTF SFPVRHEDID KGILLNWTKG 170
XP_006251241_glucokinase_isoform_1_Rattus_norvegicus PEDAMTGTAE MLFDYISECI SDFLDKHQMK HKKLPLGFTF SFPVRHEDLD KGILLNWTKG 170
NP_001095772.1_glucokinase_Bos_taurus PEDAMTGTAE MLFDYISECI SDFLDKHQMK HKKLPLGFTF SFPVRHEDID KGILLNWTKG 170
XP_010725006_glucokinase_Meleagris_gallopavo PEDAMTGTAE MLFDYISECI SDFLDKHQMK HKKLPLGFTF SFPVRHEDID KGILLNWTKG 170
XP_011573674_glucokinase_Aquila_chrysaetos PEDAMTGTAE MLFDYISECI SDFLDKHQMK HKKLPLGFTF SFPVRHEDID KGILLNWTKG 170
XP_005057963_glucokinase_Ficedula albicollis PEDAMTGTAE MLFDYISECI SDFLDKHQMK HKKLPLGFTF SFPVRHEDID KGILLNWTKG 170
NP_001079298_glucokinase_Xenopus laevis PVDAMTGTAE MLFDYIAECI SDYLDQQNMK HKKLPLGFTF SFPVRHEDID KGILLNWTKG 163
XP_018422966_glucokinase_Nanorana_parkeri PEDAMTGTAK MLFDYIAECI SDYLDQQNMK HKKLPLGFTF SFPVRHEDID KGILLNWTKG 163
XP_003224263_glucokinase_Anolis_carolinensis PEDAMTGTAE MLFDYISECI SDFLDKHQMK HKKLPLGFTF SFPVRHEDID KGILLNWTKG 170
XP_006625388_glucokinase_Lepisosteus oculatus PEDAMTGTAE MLFDYISECI SDFLDKHNMK HKKLPLGFTF SFPVRHEDID KGILLNWTKG 170
NP_001038850_glucokinase_Danio_rerio PEDAMTGTAE MLFDYIAGCI SDFLDKHNLK HKKLPLGFTF SFPVRHEDLD KGILLNWTKG 180
ACD37722_glucokinase_Cyprinus_carpio PEDAMTGTAE MLFDYIASCI SDFLDKHNLK HKKLPLGFTF SFPVRHEDLD KGILLNWTKG 180
Consensus PEDAMTGTAE MLFDYISECI SDFLDKHQMK HKKLPLGFTF SFPVRHEDID KGILLNWTKG
100%
Conservation
0%
200 220 240
NP_000153.1_glucokinase_isoform_1_Homo_sapiens FKASGAEGNN VVGLLRDAIK RRGDFEMDVV AMVNDTVATM ISCYYEDHQC EVGMIVGTGC 230
NP_034422.2_glucokinase_isoform_1_Mus_musculus FKASGAEGNN IVGLLRDAIK RRGDFEMDVV AMVNDTVATM ISCYYEDRQC EVGMIVGTGC 230
XP_006251241_glucokinase_isoform_1_Rattus_norvegicus FKASGAEGNN IVGLLRDAIK RRGDFEMDVV AMVNDTVATM ISCYYEDRQC EVGMIVGTGC 230
NP_001095772.1_glucokinase_Bos_taurus FKASGAEGNN IVGLLRDAIK RRGDFEMDVV AMVNDTVATM ISCYYEDRRC EVGMIVGTGC 230
XP_010725006_glucokinase_Meleagris_gallopavo FKASGAEGNN VVGLLRDAIK RRGDFEMDVV AMVNDTVATM ISCYYEDHRC EVGMIVGTGC 230
XP_011573674_glucokinase_Aquila_chrysaetos FKASGAEGNN VVGLLRDAIK RRGDFEMDVV AMVNDTVATM ISCYYEDHRC EVGMIVGTGC 230
XP_005057963_glucokinase_Ficedula albicollis FKASGAEGNN VVGLLRDAIK RRGDFEMDVV AMVNDTVATM ISCYYEDHRC EVGMIVGTGC 230
NP_001079298_glucokinase_Xenopus laevis FKASGAEGNN VVGLLRDAIK RRGDFEMDVV AMVNDTVATM ISCYYEDHHC EVGLIVGTGC 223
XP_018422966_glucokinase_Nanorana_parkeri FKASGAEGNN VVGLLRDAIK RRGDFEMDVV AMVNDTVATM ISCYYEDHHC EVGLIVGTGC 223
XP_003224263_glucokinase_Anolis_carolinensis FKASGAEGNN VVGLLRDAIK RRGDFEMDVV AMVNDTVATM ISCYYEDHRC EVGMIVGTGC 230
XP_006625388_glucokinase_Lepisosteus oculatus FKASGAEGNN VVGLLRDAIK RRGDFEMDVV AMVNDTVATM ISCYYEDRSC EVGMIVGTGC 230
NP_001038850_glucokinase_Danio_rerio FKASGAEGNN VVGLLRDAIK RRGDFEMDVV AMVNDTVATM ISCYYEDRSC EVGMIVGTGC 240
ACD37722_glucokinase_Cyprinus_carpio FKASGAEGNN VVGLLRDAIK RRGDFEMDVV AMVNDTVATM ISCYYEDRSC EVGMIVGTGC 240
Consensus FKASGAEGNN VVGLLRDAIK RRGDFEMDVV AMVNDTVATM ISCYYEDHRC EVGMIVGTGC
100%
Conservation
0%
260 280 300
NP_000153.1_glucokinase_isoform_1_Homo_sapiens NACYMEEMQN VELVEGDEGR MCVNTEWGAF GDSGELDEFL LEYDRLVDES SANPGQQLYE 290
NP_034422.2_glucokinase_isoform_1_Mus_musculus NACYMEEMQN VELVEGDEGR MCVNTEWGAF GNSGELDEFL LEYDRMVDES SVNPGQQLYE 290
XP_006251241_glucokinase_isoform_1_Rattus_norvegicus NACYMEEMQN VELVEGDEGR MCVNTEWGAF GDSGELDEFL LEYDRMVDES SANPGQQLYE 290
NP_001095772.1_glucokinase_Bos_taurus NACYMEEMQN VELVEGDEGR MCVNTEWGAF GDSGELDEFL LEYDRVVDEN SLNPGQQLYE 290
XP_010725006_glucokinase_Meleagris_gallopavo NACYMEEMHN VELVEGDEGR MCVNTEWGAF GASGELDEFL LEYDRVVDET SLNPGQQLYE 290
XP_011573674_glucokinase_Aquila_chrysaetos NACYMEEMQN VELVEGDEGR MCVNTEWGAF GASGELDEFL LEYDRVVDET SLNPGQQLYE 290
XP_005057963_glucokinase_Ficedula albicollis NACYMEEMHN VELVEGDEGR MCVNTEWGAF GASGELDEFL LEYDRVVDET SLNPGQQLYE 290
NP_001079298_glucokinase_Xenopus laevis NACYMEEMSN VELVEGEEGR MCVNTEWGAF GDTGELEDFR LEYDRVVDEA SLNPGQQLYE 283
XP_018422966_glucokinase_Nanorana_parkeri NACYMEEMCN VELVEGEEGR MCVNTEWGAF GDTGELEDFR LEYDRVIDEG SLNPGQQLYE 283
XP_003224263_glucokinase_Anolis_carolinensis NACYMEEMQN VELVEGNEGR MCVNTEWGAF GASGELDEFL LEYDHVVDET SLNPGQQLYE 290
XP_006625388_glucokinase_Lepisosteus oculatus NACYMEEMRT VELVEGEEGR MCVNTEWGAF GDTGELEEFR LEYDRVVDET SLNPGQQLYE 290
NP_001038850_glucokinase_Danio_rerio NACYMEEMRK VELVEGEEGR MCVNTEWGAF GDHSELEDFR LEYDRVIDET SLNPGHQLYE 300
ACD37722_glucokinase_Cyprinus_carpio NACYMEEMRK VELVEGEEGR MCVNTEWGAF GDNGELEDFR LEYDRVIDET SLNPGHQLYE 300
Consensus NACYMEEMQN VELVEGDEGR MCVNTEWGAF GDSGELDEFL LEYDRVVDET SLNPGQQLYE
100%
Conservation
0%

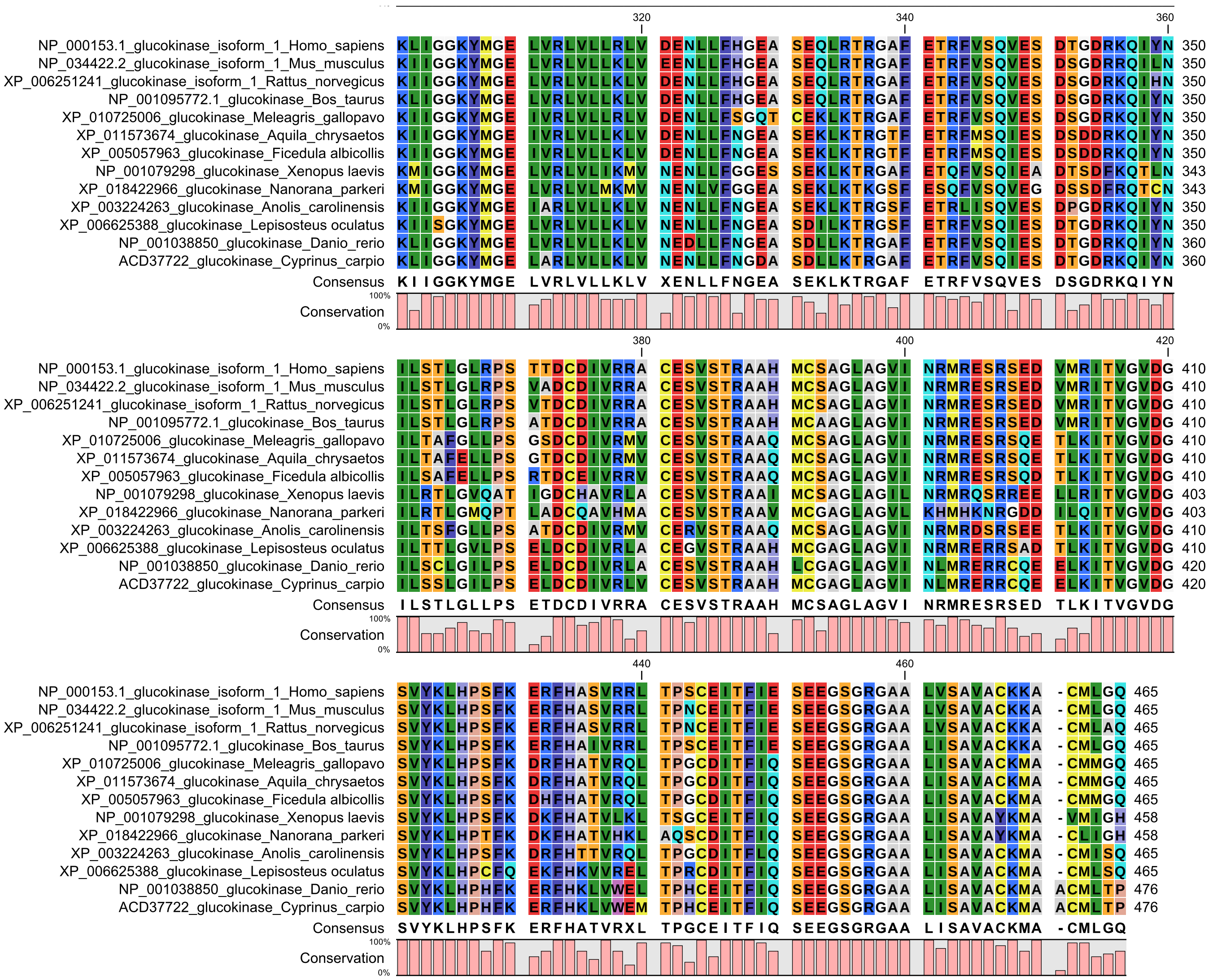
320 340 360
NP_000153.1_glucokinase_isoform_1_Homo_sapiens KLIGGKYMGE LVRLVLLRLV DENLLFHGEA SEQLRTRGAF ETRFVSQVES DTGDRKQIYN 350
NP_034422.2_glucokinase_isoform_1_Mus_musculus KLIGGKYMGE LVRLVLLKLV EENLLFHGEA SEQLRTRGAF ETRFVSQVES DSGDRRQILN 350
XP_006251241_glucokinase_isoform_1_Rattus_norvegicus KLIGGKYMGE LVRLVLLKLV DENLLFHGEA SEQLRTRGAF ETRFVSQVES DSGDRKQIHN 350
NP_001095772.1_glucokinase_Bos_taurus KLIGGKYMGE LVRLVLLKLV DENLLFHGEA SEQLRTRGAF ETRFVSQVES DSGDRKQIYN 350
XP_010725006_glucokinase_Meleagris_gallopavo KIIGGKYMGE IVRLVLLRLV DENLLFSGQT CEKLKTRGAF ETRFVSQVES DSGDRKQIYN 350
XP_011573674_glucokinase_Aquila_chrysaetos KIIGGKYMGE IVRLVLLKLV DENLLFNGEA SEKLKTRGTF ETRFMSQIES DSDDRKQIYN 350
XP_005057963_glucokinase_Ficedula albicollis KIIGGKYMGE IVRLVLLKLV DENLLFNGEA SEKLKTRGTF ETRFMSQIES DSDDRKQIYN 350
NP_001079298_glucokinase_Xenopus laevis KMIGGKYMGE LVRLVLIKMV NENLLFGGES SEKLKTRGAF ETQFVSQIEA DTSDFKQTLN 343
XP_018422966_glucokinase_Nanorana_parkeri KMIGGKYMGE LVRLVLMKMV NENLVFGGEA SEKLKTKGSF ESQFVSQVEG DSSDFRQTCN 343
XP_003224263_glucokinase_Anolis_carolinensis KIIGGKYMGE IARLVLLKLV NENLLFNGEA SEKLKTRGSF ETRLISQVES DPGDRKQIYN 350
XP_006625388_glucokinase_Lepisosteus oculatus KIISGKYMGE LVRLVLLKLV NENLLFNGEA SDILKTRGSF ETRFVSQIES DTGDRKQIYN 350
NP_001038850_glucokinase_Danio_rerio KLIGGKYMGE LVRLVLLKLV NEDLLFNGEA SDLLKTRGAF ETRFVSQIES DTGDRKQIYN 360
ACD37722_glucokinase_Cyprinus_carpio KLIGGKYMGE LARLVLLKLV NENLLFNGDA SDLLKTRGAF ETRFVSQIES DTGDRKQIYN 360
Consensus KIIGGKYMGE LVRLVLLKLV XENLLFNGEA SEKLKTRGAF ETRFVSQVES DSGDRKQIYN
100%
Conservation
0%
380 400 420
NP_000153.1_glucokinase_isoform_1_Homo_sapiens ILSTLGLRPS TTDCDIVRRA CESVSTRAAH MCSAGLAGVI NRMRESRSED VMRITVGVDG 410
NP_034422.2_glucokinase_isoform_1_Mus_musculus ILSTLGLRPS VADCDIVRRA CESVSTRAAH MCSAGLAGVI NRMRESRSED VMRITVGVDG 410
XP_006251241_glucokinase_isoform_1_Rattus_norvegicus ILSTLGLRPS VTDCDIVRRA CESVSTRAAH MCSAGLAGVI NRMRESRSED VMRITVGVDG 410
NP_001095772.1_glucokinase_Bos_taurus ILSTLGLRPS ATDCDIVRRA CESVSTRAAH MCAAGLAGVI NRMRESRSED VMRITVGVDG 410
XP_010725006_glucokinase_Meleagris_gallopavo ILTAFGLLPS GSDCDIVRMV CESVSTRAAQ MCSAGLAGVI NRMRESRSQE TLKITVGVDG 410
XP_011573674_glucokinase_Aquila_chrysaetos ILTAFELLPS GTDCDIVRMV CESVSTRAAQ MCSAGLAGVI NRMRESRSQE TLKITVGVDG 410
XP_005057963_glucokinase_Ficedula albicollis ILSAFELLPS RTDCEIVRRV CESVSTRAAQ MCSAGLAGVI NRMRESRSQD TLKITVGVDG 410
NP_001079298_glucokinase_Xenopus laevis ILRTLGVQAT IGDCHAVRLA CESVSTRAAI MCSAGLAGIL NRMRQSRREE LLRITVGVDG 403
XP_018422966_glucokinase_Nanorana_parkeri ILRTLGMQPT LADCQAVHMA CESVSTRAAV MCGAGLAGVL KHMHKNRGDD ILQITVGVDG 403
XP_003224263_glucokinase_Anolis_carolinensis ILTSFGLLPS ATDCDIVRMV CERVSTRAAQ MCSAGLAGVI NRMRDSRSEE TLKITVGVDG 410
XP_006625388_glucokinase_Lepisosteus oculatus ILTTLGVLPS ELDCDIVRLA CEGVSTRAAH MCGAGLAGVI NRMRERRSAD TLKITVGVDG 410
NP_001038850_glucokinase_Danio_rerio ILSCLGILPS ELDCDIVRLA CESVSTRAAH LCGAGLAGVI NLMRERRCQE ELKITVGVDG 420
ACD37722_glucokinase_Cyprinus_carpio ILSSLGILPS ELDCDIVRLV CESVSTRAAH MCGAGLAGVI NLMRERRCQE ELKITVGVDG 420
Consensus ILSTLGLLPS ETDCDIVRRA CESVSTRAAH MCSAGLAGVI NRMRESRSED TLKITVGVDG
100%
Conservation
0%
440 460
NP_000153.1_glucokinase_isoform_1_Homo_sapiens SVYKLHPSFK ERFHASVRRL TPSCEITFIE SEEGSGRGAA LVSAVACKKA -CMLGQ 465
NP_034422.2_glucokinase_isoform_1_Mus_musculus SVYKLHPSFK ERFHASVRRL TPNCEITFIE SEEGSGRGAA LVSAVACKKA -CMLGQ 465
XP_006251241_glucokinase_isoform_1_Rattus_norvegicus SVYKLHPSFK ERFHASVRRL TPNCEITFIE SEEGSGRGAA LVSAVACKKA -CMLAQ 465
NP_001095772.1_glucokinase_Bos_taurus SVYKLHPSFK ERFHAIVRRL TPSCEITFIE SEEGSGRGAA LISAVACKKA -CMLGQ 465
XP_010725006_glucokinase_Meleagris_gallopavo SVYKLHPSFK DRFHATVRQL TPGCDITFIQ SEEGSGRGAA LISAVACKMA -CMMGQ 465
XP_011573674_glucokinase_Aquila_chrysaetos SVYKLHPSFK DRFHATVRQL TPGCDITFIQ SEEGSGRGAA LISAVACKMA -CMMGQ 465
XP_005057963_glucokinase_Ficedula albicollis SVYKLHPSFK DHFHATVRQL TPGCDITFIQ SEEGSGRGAA LISAVACKMA -CMMGQ 465
NP_001079298_glucokinase_Xenopus laevis SVYKLHPSFK DKFHATVLKL TSGCEITFIQ SEEGSGRGAA LISAVAYKMA -VMIGH 458
XP_018422966_glucokinase_Nanorana_parkeri SVYKLHPTFK DKFHATVHKL AQSCDITFIQ SEEGSGRGAA LISAVAYKMA -CLIGH 458
XP_003224263_glucokinase_Anolis_carolinensis SVYKLHPSFK DRFHTTVRQL TPGCDITFLQ SEEGSGRGAA LISAVACKMA -CMISQ 465
XP_006625388_glucokinase_Lepisosteus oculatus SVYKLHPCFQ EKFHKVVREL TPRCDITFIQ SEEGSGRGAA LISAVACKMA -CMLSQ 465
NP_001038850_glucokinase_Danio_rerio SVYKLHPHFK ERFHKLVWEL TPHCEITFIQ SEEGSGRGAA LISAVACKMA ACMLTP 476
ACD37722_glucokinase_Cyprinus_carpio SVYKLHPHFK ERFHKLVWEM TPHCEITFIQ SEEGSGRGAA LISAVACKMA ACMLTP 476
Consensus SVYKLHPSFK ERFHATVRXL TPGCEITFIQ SEEGSGRGAA LISAVACKMA -CMLGQ
100%
Conservation
0%

Suppl. Table S1. Raw data used for the formation of Fig. 2. The table contains the numbers of families affected by disease-causing GCK nonsynonymous substitutions identified globally until year 2017, and these frequencies are matched with the GV values for each amino acid within the GCK molecule.

Position	Amino acid	GV score	Frequency of mutations [number of families]
1	M	353.86	1
2	L	353.86	0
3	D	353.86	0
4	D	353.86	1
5	R	353.86	0
6	A	353.86	0
7	R	243.26	0
8	M	91.64	1
9	E	168.08	0
10	A	152.19	0
11	A	218.83	0
12	K	173.05	0
13	K	125.10	0
14	E	107.83	0
15	K	108.93	1
16	V	64.43	2
17	E	44.60	0
18	Q	42.81	0
19	I	10.12	1
20	L	0	3
21	A	99.13	0
22	E	0	0
23	F	0	1
24	Q	116.58	0
25	L	0	0
26	Q	124.29	0
27	E	56.87	0
28	E	0	1
29	D	44.60	0
30	L	0	2
31	K	128.24	0
32	K	126.69	0
33	V	31.78	6
34	M	14.30	1
35	R	237.70	0
36	R	0	30
37	M	14.30	1
38	Q	42.81	2
39	K	116.42	0
40	E	0	25
41	M	0	3
42	D	44.60	0
43	R	0	22
44	G	0	34
45	L	0	0
46	R	38.73	1
47	L	31.78	0
48	E	0	0
49	T	0	1
50	H	68.35	8
51	E	105.70	0
52	E	0	0
53	A	0	3
54	S	0	0
55	V	0	2
56	K	0	0
57	M	0	3
58	L	0	1
59	P	0	10

60	T	0	4
61	Y	0	3
62	V	0	7
63	R	0	0
64	S	0	1
65	T	0	3
66	P	0	0
67	E	44.60	0
68	G	0	4
69	S	0	0
70	E	0	7
71	V	0	2
72	G	0	25
73	D	0	2
74	F	0	0
75	L	0	0
76	S	99.13	4
77	L	0	3
78	D	0	5
79	L	0	0
80	G	0	5
81	G	0	8
82	T	0	3
83	N	0	0
84	F	0	0
85	R	0	0
86	V	0	0
87	M	0	0
88	L	0	0
89	V	0	0
90	K	0	0
91	V	0	1
92	G	0	0
93	E	0	0
94	G	93.77	0
95	E	171.76	0
96	E	134.33	0
97	G	125.13	0
98	Q	154.67	0
99	W	0	7
100	S	123.62	0
101	V	0	2
102	K	101.46	0
103	T	0	4
104	K	77.74	0
105	H	83.33	0
106	Q	53.64	1
107	M	0	0
108	Y	0	10
109	S	0	0
110	I	0	2
111	P	0	1
112	E	121.33	0
113	D	0	0
114	A	0	1
115	M	0	1
116	T	0	1
117	G	0	2
118	T	0	1
119	A	0	1
120	E	56.87	1
121	M	0	0
122	L	0	5
123	F	0	2
124	D	0	7
125	Y	0	1
126	I	0	0

127	S	99.13	1
128	E	107.72	0
129	C	0	28
130	I	0	7
131	S	0	3
132	D	0	1
133	F	21.61	1
134	L	0	6
135	D	0	0
136	K	53.23	0
137	H	24.08	2
138	Q	45.75	3
139	M	14.30	0
140	K	0	0
141	H	0	0
142	K	0	0
143	K	0	0
144	L	0	1
145	P	0	0
146	L	0	7
147	G	0	0
148	F	0	1
149	T	0	5
150	F	0	15
151	S	0	4
152	F	0	3
153	P	0	0
154	V	0	2
155	R	0	1
156	H	0	2
157	E	0	4
158	D	0	0
159	I	4.86	1
160	D	0	9
161	K	0	1
162	G	0	3
163	I	0	0
164	L	0	7
165	L	0	2
166	N	0	0
167	W	0	1
168	T	0	7
169	K	0	3
170	G	0	4
171	F	0	4
172	K	0	0
173	A	0	5
174	S	0	2
175	G	0	13
176	A	0	1
177	E	0	1
178	G	0	10
179	N	0	0
180	N	0	14
181	V	29.61	1
182	V	0	27
183	G	0	0
184	L	0	0
185	L	0	2
186	R	0	2
187	D	0	3
188	A	0	32
189	I	0	1
190	K	0	0
191	R	0	78
192	R	0	2
193	G	0	3

194	D	0	0
195	F	0	0
196	E	0	0
197	M	0	3
198	D	0	1
199	V	0	1
200	V	0	4
201	A	0	3
202	M	0	7
203	V	0	18
204	N	0	0
205	D	0	6
206	T	0	35
207	V	0	2
208	A	0	8
209	T	0	16
210	M	0	10
211	I	0	1
212	S	0	2
213	C	0	9
214	Y	0	3
215	Y	0	0
216	E	0	0
217	D	0	2
218	H	28.82	0
219	Q	111.62	0
220	C	0	3
221	E	0	8
222	V	0	0
223	G	0	40
224	M	14.30	4
225	I	0	6
226	V	0	42
227	G	0	4
228	T	0	26
229	G	0	3
230	C	0	1
231	N	0	4
232	A	0	3
233	C	0	4
234	Y	0	1
235	M	0	9
236	E	0	5
237	E	0	1
238	M	0	0
239	Q	193.50	7
240	N	105.58	3
241	V	0	3
242	E	0	0
243	L	0	1
244	V	0	5
245	E	0	0
246	G	0	4
247	D	48.73	0
248	E	0	2
249	G	0	5
250	R	0	6
251	M	0	13
252	C	0	6
253	V	0	3
254	N	0	4
255	T	0	6
256	E	0	20
257	W	0	2
258	G	0	11
259	A	0	16
260	F	0	1

261	G	0	49
262	D	126.14	0
263	S	99.45	9
264	G	55.27	5
265	E	0	15
266	L	0	2
267	D	44.60	0
268	E	44.60	3
269	F	0	1
270	L	101.88	0
271	L	0	0
272	E	0	0
273	Y	0	2
274	D	0	4
275	R	28.82	8
276	L	31.78	1
277	V	29.61	2
278	D	0	10
279	E	0	4
280	S	127.17	0
281	S	0	5
282	A	96.19	0
283	N	0	0
284	P	0	0
285	G	0	0
286	Q	24.08	0
287	Q	0	4
288	L	0	0
289	Y	0	8
290	E	0	1
291	K	0	3
292	L	14.30	1
293	I	0	1
294	G	55.27	6
295	G	0	2
296	K	0	0
297	Y	0	1
298	M	0	6
299	G	0	10
300	E	0	6
301	L	4.86	0
302	V	64.43	4
303	R	0	7
304	L	0	3
305	V	0	0
306	L	0	2
307	L	14.30	1
308	R	26.00	4
309	L	14.30	2
310	V	0	0
311	D	48.73	0
312	E	0	1
313	N	23.01	0
314	L	0	1
315	L	31.78	13
316	F	0	1
317	H	118.22	0
318	G	0	23
319	E	61.28	1
320	A	103.68	0
321	S	111.67	0
322	E	44.60	0
323	Q	125.10	0
324	L	0	3
325	R	26	0
326	T	0	1
327	R	26	1

328	G	0	0
329	A	103.68	0
330	F	0	0
331	E	0	1
332	T	57.75	1
333	R	42.81	0
334	F	21.82	0
335	V	29.61	0
336	S	0	3
337	Q	0	1
338	V	29.61	0
339	E	0	10
340	S	103.39	5
341	D	0	0
342	T	79.02	2
343	G	95.09	0
344	D	0	1
345	R	97.03	0
346	K	26.00	0
347	Q	0	0
348	I	89.28	2
349	Y	224.33	0
350	N	0	0
351	I	0	0
352	L	0	1
353	S	111.49	0
354	T	200.43	0
355	L	21.82	0
356	G	97.85	1
357	L	31.78	0
358	R	116.23	0
359	P	26.87	1
360	S	57.75	0
361	T	182.66	0
362	T	162.25	0
363	D	0	2
364	C	0	0
365	D	81.24	1
366	I	93.66	0
367	V	0	2
368	R	28.82	0
369	R	102.84	2
370	A	64.43	0
371	C	0	10
372	E	0	0
373	S	135.70	1
374	V	0	5
375	S	0	1
376	T	0	0
377	R	0	10
378	A	0	17
379	A	0	5
380	H	108.82	6
381	M	14.30	6
382	C	0	6
383	S	103.39	30
384	A	0	5
385	G	0	5
386	L	0	6
387	A	0	10
388	G	0	1
389	V	29.61	7
390	I	4.86	0
391	N	93.88	0
392	R	104.93	1
393	M	0	5
394	R	28.82	0

395	E	105.70	0
396	S	113.99	0
397	R	0	8
398	S	205.85	0
399	E	135.14	0
400	D	44.60	0
401	V	144.57	0
402	M	14.30	0
403	R	56.64	0
404	I	0	4
405	T	0	2
406	V	0	3
407	G	0	5
408	V	0	0
409	D	0	0
410	G	0	0
411	S	0	3
412	V	0	4
413	Y	0	3
414	K	0	2
415	L	0	1
416	H	0	3
417	P	0	0
418	S	180.83	0
419	F	0	4
420	K	53.23	2
421	E	44.60	0
422	R	38.73	2
423	F	0	4
424	H	0	2
425	A	114.06	0
426	S	144.08	0
427	V	0	0
428	R	125.81	1
429	R	64.93	0
430	L	14.30	0
431	T	58.02	0
432	P	97.59	0
433	S	141.87	0
434	C	0	9
435	E	44.60	0
436	I	0	4
437	T	0	0
438	F	0	2
439	I	4.86	0
440	E	29.27	1
441	S	0	8
442	E	0	1
443	E	0	0
444	G	0	0
445	S	0	1
446	G	0	1
447	R	0	10
448	G	0	0
449	A	0	7
450	A	0	6
451	L	0	0
452	V	29.61	2
453	S	0	10
454	A	0	4
455	V	0	7
456	A	0	3
457	C	193.72	1
458	K	0	0
459	K	94.49	0
460	A	0	0
461	C	191.23	0

462	M	14.30	1
463	L	14.30	0
464	G	115.24	1
465	Q	83.40	1

Supplementary references for Suppl. Table S1:

Agladioglu SY, Aycan Z, Cetinkaya S, et al. (2016) Maturity onset diabetes of youth (MODY) in Turkish children: sequence analysis of 11 causative genes by next generation sequencing. J Pediatr Endocrinol Metab 29:487–496

Ajala ON, Huffman DM, Ghobrial II (2016) Glucokinase mutation – a rare cause of recurrent hypoglycemia in adults: a case report and literature review. J Community Hosp Intern Med Perspect 26:32983

Almeida C, Silva SR, Garcia E, Leite AL, Teles A, Campos RA (2014) A novel genetic mutation in a Portuguese family with GCK-MODY. J Pediatr Endocrinol Metab 27:129–133

Ang SF, Lim SC, Tan CSH, et al. (2016) A preliminary study to evaluate the strategy of combining clinical criteria and next generation sequencing (NGS) for the identification of monogenic diabetes among multi-ethnic Asians. Diabetes Res Clin Pract 119:13–22

Antosik K, Gnys P, De Franco E, et al. (2016) Single patient in GCK-MODY family successfully re-diagnosed into GCK-PNDM through targeted next-generation sequencing technology. Acta Diabetol 53:337–338

Barbetti F, Cobo-Vuilleumier N, Dionisi-Vici C, et al. (2009) Opposite clinical phenotypes of glucokinase disease: description of a novel activating mutation and contiguous inactivating mutations in human glucokinase (GCK) gene. Mol Endocrinol 23:1983–1989

Bazalová Z, Rypáčková B, Brož J, et al. (2010) Three novel mutations in MODY and its phenotype in three different Czech families. Diabetes Res Clin Pract 88:132–138

Beer NL, van de Bunt M, Colclough K, et al. (2011) Discovery of a novel site regulating glucokinase activity following characterization of a new mutation causing hyperinsulinemic hypoglycemia in humans. J Biol Chem 286:19118–19126

Beer NL, Osbak KK, van de Bunt, et al. (2012) Insights into the pathogenicity of rare missense GCK variants from the identification and functional characterization of compound heterozygous and double mutations inherited in cis. Diabetes Care 35:1482–1484

Bennett JT, Vasta V, Zhang M, NarayananJ, Gerrits P, Hahn SH (2015) Molecular genetic testing of patients with monogenic diabetes and hyperinsulinism. Mol Genet Metab 114:451–458

Borowiec M, Antosik K, Fendler W, et al. (2012) Novel glucokinase mutations in patients with monogenic diabetes – clinical outline of GCK-MD and potential for founder effect in Slavic population. Clin Genet 81:278–283

Borowiec M, Fendler W, Antosik K, et al. (2012) Doubling the referral rate of monogenic diabetes through a nationwide information campaign – update on glucokinase gene muations in a Polish cohort. Clin Genet 82:587–590

Braha N, De Franco E, Dawes A, et al. (2016) Permanent neonatal diabetes mellitus due to a novel homozygous GCK mutation in a premature baby with IUGR and its management. Horm Res Paediatr 86(Suppl 1):208

Caetano LA, Jorge AAL, Malaquias AC, et al. (2012) Incidental mild hyperglycemia in children: two MODY 2 families identified in Brazilian subjects. Arq Bras Endocrinol Metab 56:519–524

Calcaterra V, Martinetti M, Salina A, Aloi C, Larizza D (2011) The coexistence of type 1 diabetes, MODY2 and metabolic syndrome in a young girl. Acta Diabetol 49:401–404

Capuano M, Garcia-Herrero CM, Tinto N, et al. (2012) Glucokinase (GCK) mutations and their characterization in MODY2 children of Southern Italy. PLoS ONE 7:e38906

Carmody D, Naylor RN, Bell CD, et al. (2016) GCK-MODY in the US National Monogenic Diabetes Registry: frequently misdiagnosed and unnecessarily treated. Acta Diabetol 53:703–708

Challis BG, Harris J, Sleigh A, et al. (2014) Familial adult onset hyperinsulinism due to an activating glucokinase mutation: implications for pharmacological glucokinase activation. Clin Endocrinol 81:855–861

Constantini S, Malerba G, Contreas G, et al. (2015) Genetic and bioinformatics analysis of four novel GCK missense variants detected in Caucasian families with GCK-MODY phenotype. Clin Genet 87:440–447

Cuesta-Muñoz AL, Tuomi T, Cobo-Vuilleumier N, et al. (2010) Clinical heterogeneity in monogenic diabetes caused by mutations in the glucokinase gene (GCK-MODY). Diabetes Care 33:290–292

d´Annunzio G, Marchi M, Aloi C, Salina A, Lugani F, Lorini R (2013) Hyperglycaemia and β-cell antibodies: is it always pre-type 1 diabetes? Diabetes Res Clin Pract 100:e20–e22

DellaManna T, da Silva MR, Chacra AR, et al. (2012) Clinical follow-up of two Brazilian subjects with glucokinase-MODY (MODY2) with description of a novel mutation. Arq Bras Endocrinol Metab 56:490–495

Delvecchio M, Ludovico O, Bellacchio E, et al. (2013) MODY type 2 P59S GCK mutant: founder effect in South of Italy. Clin Genet 83:83–87

Demirbilek H, Arya VB, Ozbek MN, et al. (2015) Clinical characteristics and molecular genetic analysis of 22 patients with neonatal diabetes from the South-Eastern region of Turkey: predominance of non-K_{ATP} channel mutations. Eur J Endocrinol 172:697–705

Doddabelavangala Mruthyunjaya M, Chapla A, Hesarghatta Shyamasunder A, et al. (2017) Comprehensive maturity onset diabetes of the young (MODY) gene screening in pregnant women with diabetes in India. PLoS ONE 12:e0168656

Emelyanov AO, Sechko E, Koksharova E, et al. (2017) A glucokinase gene mutation in a young boy with diabetes mellitus, hyperinsulinemia, and insulin resistence. Int Med Case Rep J 10:77–80

Esquiaveto-Aun AM, De Mello MP, Paulino MF, Minicucci WJ, Guerra-Júnior, De Lemos-Marini (2015) A new compound heterozygosis for inactivating mutations in the glucokinase gene as cause of permanent neonatal diabetes mellitus (PNDM) in double-first cousins. Diabetol Metab Syndr 7:101

Fahr K, Böckmann A, Wildhardt G, Gessler P (2012) MODY 2 diabetes in a mature newborn due to a new mutation in the GCK-gene. Klin Padiatr 224:316–317

García-Herrero CM, Rubio-Cabezas O, Azriel S, et al. (2012) Functional characterization of MODY2 mutations highlights the importance of the fine-tuning of glucokinase and its role in glucose sensing. PLoS ONE 7:e30518

Giuffrida FMA, Calliari LE, DellaManna T, et al. (2013) A novel glucokinase deletion (p.Lys32del) and five previously described mutations co-segregate with the phenotype of mild familial hyperglycaemia (MODY2) in Brazilian families. Diabetes Res Clin Pract 100:e42–e45

Giuffrida FMA, Moises RS, Weinert LS, et al. (2017) Maturity-onset diabetes of the young (MODY) in Brazil: establishment of a national registry and appraisal of available genetic and clinical data. Diabetes Res Clin Pract 123:134–142

Gozlan Y, Tenenbaum A, Shalitin S, et al. (2012) The glucokinase mutation p.T206P is common among MODY patients of Jewish Ashkenazi descent. Pediatr Diabetes 13:e14–e21

Haliloglu B, Hysenaj G, Atay Z, et al. (2016) GCK gene mutations are a common cause of childhood-onset MODY (maturity-onset diabetes of the young) in Turkey. Clin Endocrinol 85:393–399

Heggie AJ, Walker M (2016) A patient with a glucokinase mutation: a cautionary tale. Diabet Med 33(Suppl 1):102

Henquin JC, Sempoux C, Marchandise J, et al. (2013) Congenital hyperinsulinism caused by hexokinase I expression or glucokinase-activating mutation in a subset of β-cells. Diabetes 62:1689–1696

Jha S, Siddiqui S, Waghdhare S, et al. (2016) Identification of a novel glucokinase mutation in an Indian woman with GCK-MODY. Lancet Diabetes Endocrinol 4:302

Juszczak A, Bartlett K, Mackillop L, Owen K (2016) Pregnancy outcome in hypoglycaemia caused by an activating glucokinase mutation. Diabet Med 33(Suppl 1):100

Kanthimathi S, Jahnavi S, Balamurugan K, et al. (2014) Glucokinase gene mutations (MODY 2) in Asian Indians. Diabetes Technol Ther 16:180–185

Kassem S, Bhandari S, Rodríguez-Bada P, et al. (2010) Large islets, beta-cells proliferation, and a glucokinase mutation. N Engl J Med 362:1348–1350

Kawakita R, Hosokawa Y, Fujimaru R, et al. (2014) Molecular and clinical characterization of glucokinase maturity-onset diabetes of the young (GCK-MODY) in Japanese patients. Diabet Med 31:1357–1362

Kuraeva TL, Sechko EA, Zilberman LI, et al. (2015) Molecular genetics and clinical variants MODY2 and MODY3 in children in Russia. Probl Endocrinol 61:14–25

Li Q, Cao X, Qiu HY, Lu J, et al. (2016) A three-step programmed method for the identification of causative gene mutations of maturity onset diabetes of the young (MODY). Gene 588:141–148

Lopez AP, de Dios A, Chiesa I, Perez MS, Frechtel GD (2016) Analysis of mutations in the glucokinase gene in people clinically characterized as MODY2 without a family history of diabetes. Diabetes Res Clin Pract 118:38–43

Marks SD, Couch RM (2010) Identification of two new mutations in the glucokinase gene that result in maturity-onset diabetes of the young. Diabetes Care 33:e94

Martínez R, Gutierrez-Nogués A, Fernández-Ramos C, et al. (2017) Heterogeneity in phenotype of hyperinsulinism caused by activating glucokinase mutations: a novel mutation and its characterization. Clin Endocrinol, doi: 10.1111/cen.13318

Morishita K, Kyo C, Yonemoto T, Kosugi R, Ogawa T, Inoue T (2017) Asymptomatic congenital hyperinsulinism due to a glucokinase-activating mutation, treated as adrenal insufficiency for twelve years. Case Rep Endocrinol 2017:4709262

Negahdar M, Aukrust I, Molnes J, et al. (2014) GCK-MODY diabetes as a protein misfolding disease: the mutation R275C promotes protein misfolding, self-association and cellular degradation. Mol Cell Endocrinol 382:55–65

Osbak KK, Colclough K, Saint-Martin C, et al. (2009) Update on mutations in glucokinase (GCK), which cause maturity-onset diabetes of the young, permanent neonatal diabetes, and hyperinsulinemic hypoglycemia. Hum Mutat 30:1512–1526

Papadimitriou DT, Willems PJ, Bothou C, Karpathios T, Papadimitriou A (2015) A novel heterozygous mutation in the glucokinase gene is responsible for an early-onset mild form of maturity-onset diabetes of the young, type 2. Diabetes Metab 41:342–345

Pihoker C, Gilliam LK, Ellard S, et al. (2013) Prevalence, characterictics and clinical diagnosis of maturity-onset diabetes of the young due to mutations in HNF1A, HNF4A, and glucokinase: results from the SEARCH for diabetes in youth. J Clin Endocrinol Metab 98:4055–4062

Ping Xiao Y, Hua Xu X, Lan Fang Y, et al. (2016) GCK mutations in Chinese MODY2 patients: a family pedigree report and review of Chinese literature. J Pediatr Endocrinol Metab 29:959–964

Pruhova S, Dusatkova P, Sumnik Z, et al. (2010) Glucokinase diabetes in 103 families from a country-based study in the Czech Republic: geographically restricted distribution of two prevalent GCK mutations. Pediatr Diabetes 11:529–535

Pulst K, Arbo T, Kahre T, Peet A, Tillmann V (2012) MODY2 caused by a novel mutation of GCK gene. J Pediatr Endocrinol Metab 25:801–803

Raimondo A, Chakera AJ, Thomsen SK, et al. (2014) Phenotypic severity of homozygous GCK mutations causing neonatal or childhood-onset diabetes is primarily mediated through effects on protein stability. Hum Mol Genet 23:6432–6440

Shammas C, Neocleous V, Phelan MM, Lian LY, Skordis N, Phylactou LA (2013) A report of 2 new cases of MODY2 and review of the literature: implications in the search for type 2 diabetes drugs. Metabolism 62:1535–1542

Shen Y, Cai M, Liang H, Wang H, Weng J (2011) Insight into the biochemical characteristics of a novel glucokinase gene mutation. Hum Genet 129:231–238

Shoemaker AH, Zienkiewicz J, Moore DJ (2012) Clinical assessment of HNF1A and GCK variants and identification of a novel mutation causing MODY2. Diabetes Res Clin Pract 96:e36–e39

Stanik J, Dusatkova P, Cinek O, et al. (2014) De novo mutations of GCK, HNF1A and HNF4A may be more frequent in MODY than previously assumed. Diabetologia 57:480–484

Steele AM, Tribble ND, Caswell R, et al. (2011) The previsouly reported T342P GCK missense variant is not a pathogenic mutation causing MODY. Diabetologia 54:2202–2205

Szopa M, Ludwig-Galezowska A, Radkowski P, et al. (2015) Genetic testing for monogenic diabetes using targeted next-generation sequencing in patients with maturity-onset diabetes of the young. Pol Arch Med Wewn 125:845–851

Valentínová L, Beer NL, Staník J, et al. (2012) Identification and functional characterisation of novel glucokinase mutations causing maturity-onset diabetes of the young in Slovakia. PLoS ONE 7:e34541

Wang Z, Ping F, Zhang Q, et al. (2017) Preliminary screening of mutations in the glucokinase gene of Chinese patients with gestational diabetes. J Diabetes Investig, doi: 10.1111/jdi.12664

Weinert LS, Silveiro SP, Giuffrida FMA, et al. (2014) Three unreported glucokinase (GCK) missense mutations detected in the screening of thirty-two Brazilian kindreds for GCK and HNF1A-MODY. Diabetes Res Clin Pract 106:e44–e48

Yellapu NK, Valasani KR, Pasupuleti SK, Gopal S, Potukuchi Venkata Gurunadha Krishna S, Matcha B (2014) Identification and analysis of novel R308K mutation in glucokinase of type 2 diabetic patient and its kinetic correlation. Biotechnol Appl Biochem 61:572–581

Yilmaz AA, Elmaogullari S, Demirel F, et al. (2016) A novel glucokinase gen mutation: MODY type-2 case. Horm Res Paediatr 86(Suppl 1):247

Yokota I, Moritani M, Nishisho K, Miyoshi Y, Kotani Y, Kagami S (2011) Detection of glucokinase gene defects in non-obese Japanese children diagnosed with diabetes by school medical examinations. Endocr J 58:741–746

Yorifuji T, Fujimaru R, Hosokawa Y, et al. (2012) Comprehensive molecular analysis of Japanese patients with pediatric-onset MODY-type diabetes mellitus. Pediatr Diabetes 13:26–32

Ziemssen F, Bellanné-Chantelot C, Osterhoff M, Schatz H, Pfeiffer AFH (2002) To: Lindner T, Cockburn BN, Bell GI (1999). Molecular genetics of MODY in Germany. Diabetologia 42:121-123. Diabetologia 45:286–287

Exon	Variation	Enzyme kinetics						Clinical phenotype	MODY					PNDM				PHHI					Prediction methods												References	
		$S_{0.5}$ [mM]	Hill coefficient n_H	ATP K_M [mM]	k_{cat} [s^{-1}]	Relative activity index	GSIR-T [mM of glucose]		Age at diagnosis [years]	PG or FPG [mM]	PG / 120 min [mM]	C-peptide [pmol/L]	HbA_{1c} [%]	Age at diagnosis [days]	PG or FPG [mM]	C-peptide [pmol/L]	HbA_{1c} [%]	Age at diagnosis [days]	PG or FPG [mM]	PG / 120 min [mM]	C-peptide [pmol/L]	HbA_{1c} [%]	SIFT	PolyPhen-2	PhD-SNP	PoPMuSiC 2.1	SNAP2	SNPs&GO (GO terms excluded)	SNPs&GO (GO terms included)	I-Mutant 3	Align GVGD	GV score	GD score	EV mutation	Enzyme kinetics	Clinical phenotype
**Exon 1**																																				
	D4N							*GCK*-MODY															0,07	0	N		-70	N	N/D		C0	353,86	0			[1]
	M8I							*GCK*-MODY															0,04	0,525	N		-47	N	N/D		C0	91,64	8,09			[2]
	A11T							Benign															0,56	0,002	N		-40	N	N/D		C0	218,83	0			[2]
	K15M							*GCK*-MODY															0,27	0,928	N		-50	N	N/D		C15	108,93	80,92	-3,37		[3]
**Exon 2**																																				
	V16E							*GCK*-MODY															0	0,991	D	1,69	48	N/D	D	-2,35	C15	64,43	92,8	-6,42		[4]
	V16Q							*GCK*-MODY															0	0,998	D	1,57	39	N/D	D	-2,32	C15	64,43	72,42	-7,67		[2]
	E17G							*GCK*-MODY															0,01	0,176	N	0,82	-69	N	N/D	-0,83	C15	44,6	75,33	-4,39		
	I19N							*GCK*-MODY															0,3	1	D	2,16	-42	N/D	D	-1,97	C65	10,12	141,15	-7,34		[2]
	L20P							*GCK*-MODY															0	1	D	3,68	44	N/D	D	-2,46	C65	0	97,78	-8,65		[2]
	F23V							*GCK*-MODY															0,01	0,972	D	2,9	60	N/D	D	-1,37	C45	0	49,94	-6,96		[2]
	E28K							*GCK*-MODY															0,09	0,022	D	0,59	22	N/D	D	-0,67	C55	0	56,87	-2,99		[2]
	L30P							*GCK*-MODY															0	1	D	3,4	75	D	D	-2,02	C65	0	97,78	-7,84		[2]
	V33A	12,77	1,56	0,63	42,6	0,51	5,9	*GCK*-MODY	0.83	IGT													0	0,888	D	1,73	11	N	N/D	-1,7	C25	31,78	64,43	-5,24	This study	[5]
	M34R							*GCK*-MODY															0,54	1	N	1,37	-56	N	N/D	-1,7	C45	14,3	90,56	-3,16		[6]
	R36P							*GCK*-MODY															0,01	1	D	1,95	72	N/D	D	-0,71	C65	0	102,71	-7,53		[7]
	R36Q							*GCK*-MODY															0,21	1	N	0,33	-37	N	N/D	-0,83	C35	0	42,81	-3,75		[2]
	R63S	4,44	1,64	0,35	57,7	4,17	2,9																0,02	0,969	N	0,47	-12	N	N/D	-1,5	C65	0	109,21	-4,57	This study	
	R36W							*GCK*-MODY	11	6.4			6.6										0	1	D	0,42	75	N/D	D	-0,46	C65	0	101,29	-7,02		[8]
										10,4																										[9]
										8,2																										
										8,3																										
	M37R							*GCK*-MODY															0	1	D	1,37	56	N/D	D	-1,5	C45	14,3	90,56	-7,98		[2]
	Q38P							*GCK*-MODY	0.04	11.7													0,03	0,995	D	2,23	4	N/D	D	-1,1	C15	42,81	69,18	-6,31		[10]
	E40D							*GCK*-MODY															0,09	0,999	D	1,35	-66	N/D	D	-0,88	C35	0	44,6	-3,91		[11]
	E40K							*GCK*-MODY	7	IGT													0,02	1	D	0,93	16	N/D	D	-1,09	C55	0	56,87	-5,52		[5]
								PNDM						112	25	< 33.1	13,4																			[12]
	M41T							*GCK*-MODY															0	1	D	2,57	60	N/D	D	-1,87	C65	0	81,04	-8,04		[2]
	D42G																						0,07	0,872	N	1,06	-47	N	N/D	-0,97	C15	44,6	75,33	-4,32		
	R43C							PNDM						161	23.1		9.4						0,01	1	D	1,79	12	N/D	D	-0,96	C65	0	179,53	-6,96		[12]
	R43G							*GCK*-MODY															0,03	1	D	2,07	55	N/D	D	-1,41	C65	0	125,13	-6,18		[2]
	R43H							*GCK*-MODY		6,0													0,04	1	N	0,77	24	N	N/D	-1,18	C25	0	28,82	-5,54		[9]
										6,9																										
	R43P							*GCK*-MODY															0,02	1	D	1,78	60	N/D	D	-0,87	C65	0	102,71	-6,39		[2]
	R43S							*GCK*-MODY															0,03	1	D	1,12	29	N	N/D	-1,11	C65	0	109,21	-5		[2]
	G44C							*GCK*-MODY															0	1	D	0,16	2	N/D	D	-1,15	C65	0	158,23	-6,45		[2]
	G44D							*GCK*-MODY															0	1	D	1,18	58	D	D	-1,33	C65	0	93,77	-8,29		[2]
	G44S	32,2	1,59	0,23	37,2	0,08	6,9	*GCK*-MODY															0	1	D	0,77	24	N/D	D	-1,43	C55	0	55,27	-5,78	[13]	[2]
	R46S							*GCK*-MODY															0,87	0,151	N	0,33	-98	N	N/D	-0,85	C25	38,73	85,9	-1,1		[14]
	E48K																						0,2	0,955	N	0,23	-35	N	D	-0,52	C55	0	56,87	-2,77		
	T49N							*GCK*-MODY															0,01	1	D	0,77	6	N/D	D	-0,83	C55	0	64,77			[15]
	H50D							PNDM						16	31	165.5	8.7						0,03	0,947	D	1,69	17	N/D	D	-0,83	C0	68,35	23,01			[12]
	H50L							*GCK*-MODY															0,01	0,99	D	0,05	11	N	N/D	0,19	C15	68,35	98,69			[2]
	H50R							*GCK*-MODY															0,07	0,979	D	0,79	9	N	N/D	-0,5	C0	68,35	28,37			[2]
	H50Y							*GCK*-MODY		6,2	11,1												0,01	0,998	N	0,03	35	N	N/D	-0,18	C15	68,35	83,33			[16]
	A53S	8,1	1,8	0,22	45,2	0,84		*GCK*-MODY															0,33	0,999	N	0,46	-78	N	N/D	-0,9	C65	0	99,13	-2,62	[17]	[2]
		6,96	1,8	0,33	50,9	0,88	5																												[13]	
		6,8	1,61	0,37	89,87	0,995																													[18]	
	A53V							*GCK*-MODY															0,01	1	D	0,89	3	N/D	D	-0,1	C55	0	64,43	-5,62		[2]
	V55A							*GCK*-MODY															0	1	D	2,6	42	N	N/D	-1,86	C55	0	64,43	-6,14		[6]
	V55G							*GCK*-MODY															0	1	D	2,96	68	N/D	D	-2,59	C65	0	108,79	-6,72		[6]
	M57I							*GCK*-MODY															0,01	0,994	D	1,55	56	N/D	D	-1,24	C0	0	10,12	-7,33		[19]
	M57R							*GCK*-MODY															0	1	D	1,56	81	N/D	D	-1,62	C65	0	91,64	-9,27		[2]
	M57T							*GCK*-MODY															0	0,999	D	2,13	73	N/D	D	-1,71	C65	0	81,04	-8,35		[20]
	L58P							*GCK*-MODY															0	1	D	2,47	83	N/D	D	-2,21	C65	0	97,78	-6,64		[21]
	P59L							*GCK*-MODY															0,01	1	D	1,36	46	N/D	D	-0,98	C65	0	97,78	-4,01		[2]
	P59S							*GCK*-MODY															0,03	1	D	1,4	63	N/D	D	-2,02	C65	0	73,35	-4,64		[22]
	T60I							*GCK*-MODY															0	1	D	0,82	81	N/D	D	-1,3	C65	0	89,28	-9,14		[2]
	Y61C							*GCK*-MODY															0	1	D	1,85	26	N/D	D	-1,77	C65	0	193,72	-4,05		[22]
	Y61S	39,2	1,5	0,21	9.23*	0.013*	7.6*	*GCK*-MODY	4	7,5	11,4		5,7										0	1	D	2,14	71	N/D	D	-1,98	C65	0	143,11	-6,43	[23]	[23]
									35	7.8			5.8																							
	V62A	24,6	1,5	0,2	49,6	0,23		*GCK*-MODY	47	7,2	11,8		6,1										0	1	D	1,54	56	N/D	D	-2,33	C55	0	64,43	-5,33	[24]	[25]
		30,2	1,51	0,2	47,9				56	6,6	4,4		6,8																						[24]	
		27,4	1,51	0,2	48,8	0,15	6,8		18	8,6	11,7		6,4																						[13,26]	
									2	6,1	7,8		5,9																							
									2	2,9	8,7		5,8																							
	V62E	19,5	1,15	0,07	4,92	0,11	6.8*																0	1	D	1,9	83	N/D	D	-2,14	C65	0	121,33	-9,34	[26]	
	V62F	8,93	1,43	0,71	33,7	0,75	5.8*																0	1	D	0,84	52	N/D	D	-1,76	C45	0	49,94	-6,77	[26]	
	V62K	10	1,18	0,16	6,42	0,26	6.6*																0	1	D	1,64	83	N/D	D	-2,08	C65	0	97	-8,40	[26]	
	V62L	3,26	1,39	0,61	59,9	6,39	2.9*																0,02	0,998	D	0,69	45	N/D	D	-1,27	C25	0	31,78	-4,98	[26]	
	V62M	4,88	1,49	0,46	54,9	2,59	4	*GCK*-MODY	6	5,8			5,6										0	1	D	1,01	53	N/D	D	-1,44	C15	0	21,52	-5,96	[13,26]	[26]
									6	5.7			5.5																							
										6,5			6,3																							
										5.5	9.2		5.9																							
										5,4			5,7																							
									57	7,4			6,9																							
										5.4			5.7																							
										5,9			6,8																							
	V62Q	12,7	1,14	0,11	14,9	0,52	6.4*																0	1	D	1,64	77	N/D	D	-2,1	C65	0	96,33	-7,60	[26]	
	V62T	26,3	1,36	0,22	25,7	0,18	6.8*																0,01	0,999	D	1,43	60	N/D	D	-1,99	C65	0	69,5	-6,50	[26]	
	R63S					4,17	2,9																0,02	0,969	N	0,47	-12	N	N/D	-1,5	C65	0	109,21	-4,57	This study	
	S64Y	1,49	1,33	1,07	54	13,4	1,75	PHHI										1	2,0				0,01	1	N	0,17	8	N/D	D	-0,26	C65	0	143,11	-6,01	[27,28]	[28]
	T65I	1,69	1,3	0,57	22,6	5,6	3,1	PHHI										Infancy	2.3				1	0,999	N	0,02	-87	N	N/D	-0,73	C65	0	89,28	-3,10	[27]	[29]
		1,84	1,3	0,64	16,3	3,11	3,1											20	2.2																[13]	
	G68D							*GCK*-MODY															0	1	D	1,04	73	D	D	-1,15	C65	0	93,77	-5,84		[2]
	G68V	1,94	1,25	0,28	68	16,1	1,45	PHHI										Infancy	1.6				0	1	D	0,51	38	D	D	-0,63	C65	0	108,79	-6,20	[27.30]	[30.31]
																			3,0		49900															
																		Infancy	2.5		33300															
																		Infancy	2,9		40000															
																			3.3		46200															
																		Infancy	3,2		16700															
																		Infancy	3,1		36600															
	E70D							*GCK*-MODY	10	5.6 - 6.9	7.8 - 11.0		5.9										0	0,965	D	0,25	71	N/D	D	-0,64	C35	0	44,6	-6,03		[32]
	E70K	11,5	1,78	0,36	43,2	0,31		*GCK*-MODY															0	0,997	D	0,17	71	D	D	-0,83	C55	0	56,87	-5,69	[17]	[2]
		14,1	1,48	0,4	65,3	0,65	6,1																												[13]	
**Exon 3**																																				
	V71I							*GCK*-MODY															0,05	0,929	N	0,31	-69	N	N	-0,31	C25	0	29,61	-3,85		[2]
	G72R	5,5	1,5	0,77	25,5	1,01	5,3	*GCK*-MODY	13														0	1	D	1,78	91	D	D	-0,71	C65	0	125,13	-9,03	[13]	[33]
		5,32	1,45	0,76	29,9	1,71			14																										[24]	
								PNDM						1	7,9	43																				[12]
	D73G							*GCK*-MODY															0	1	D	1,12	52	N	D	-1,34	C65	0	93,77	-7,06		[2]
	S76C							*GCK*-MODY															0	0,999	N	-1,44	2	D	D	-0,97	C15	99,13	103,23	-5,52		[34]
	S76F							*GCK*-MODY															0	1	D	-0,73	40	D	D	-0,41	C15	99,13	111,66	-6,65		[2]
	S76Y							*GCK*-MODY															0	0,999	D	-0,95	38	D	D	-0,57	C15	99,13	109,77	-6,65		[2]
	L77P							*GCK*-MODY															0	1	D	5,29	80	D	D	-2,11	C65	0	97,78	-9,92		[2]
	L77R							*GCK*-MODY															0	1	D	3,47	75	D	D	-1,95	C65	0	101,88	-9,92		[35]
	D78E							*GCK*-MODY	5	5,6	8,6												0	0,999	D	1,09	67	D	D	-0,47	C35	0	44,6	-4,79		[36]
	D78G							*GCK*-MODY															0	1	D	1,06	84	D	D	-1,32	C65	0	93,77	-6,89		[2]
	D78H							*GCK*-MODY	27	6,6	6,8												0	1	D	-0,28	67	D	D	-0,52	C65	0	81,24	-5,89		[36]
	G80A	965	0,64	7,71	0,06	0,0002		*GCK*-MODY															0	1	D	1,78	71	D	D	-0,53	C55	0	60	-7,46	[17]	[2]
		13,87	0,88	2,92	0,05	0,0006																													[18]	
	G80D							*GCK*-MODY															0	1	D	2,14	89	D	D	-0,79	C65	0	93,77	-8,66		[2]
	G80S							*GCK*-MODY		6.6	11.3												0	1	D	1,56	70	D	D	-0,7	C55	0	55,27	-6,03		[16]
	G81C							*GCK*-MODY	9	6,2	11,1		6,3										0	1	D	0,74	85	D	D	-0,58	C65	0	158,23	-7,75		[37]
	G81D					< 0.01	≥ 7.1	*GCK*-MODY															0	1	D	0,69	92	D	D	-0,65	C65	0	93,77	-5,86	This study	[38]
	G81S							*GCK*-MODY															0	1	D	0,56	87	D	D	-0,6	C55	0	55,27	-5,6		[2]
	T82A	1,35	0,98	2,21	2,99	0.33*	6.9*	*GCK*-MODY	14	7,0			7,2										0,02	0,941	D	0,3	74	D	D	-0,69	C55	0	58,02	-4,89	[39]	[40]
									12	7,5			6,7																							
									5	5,8			6,3																							
	T82I							*GCK*-MODY															0	0,999	D	0,19	83	D	D	-0,44	C65	0	89,28	-7,33		[2]
	T82P							*GCK*-MODY															0	0,994	D	0	89	D	D	-0,47	C35	0	37,56	-6,93		[6]
	V91L	1,66	1,42	0,48	60,6	14,9	1,55	PHHI															0,74	0,995	N	1,35	-88	N	N	-0,88	C25	0	31,78	-3,53	[27]	[27]
	W99C	2,37	1,66	0,37	72,3	9,57	2	PHHI															0,02	0,999	D	0,72	2	N	N	-0,92	C65	0	214,36	-2,69	[27]	[41]
	W99F	6,2	1,75		46,8																		0,03	0,027	N	0,46	-13	N	N	-0,69	C35	0	39,66	-4,09	[42]	
	W99L	2,86	1,63	0,42	85,6	7,97	2,2	PHHI															0,09	0,918	D	0,68	-51	N	N	-0,37	C55	0	60,98	-2,30	[27]	[2]
	W99R	2,25	1,55	0,81	43,1	5,56	2,8	PHHI										1	1.8				1	0,998	N	0,77	-14	N	D	-0,67	C65	0	101,29	0,26	[27]	[29]
		4,49	1,39	1,35	97,3	4,14	2,8												3,1	2,1															[13]	
	V101M							*GCK*-MODY															0	0,975	D	0,81	10	N	D	-0,56	C15	0	21,52	-4,98		[2]
	T103C	11,2	1,5	0,5	52,3	0,3	5,7																0	1	D	0,57	5	N	D	-0,8	C65	0	148,93	-5,19	[43]	
	T103I	24,9	1,7	0,6	68,1	<0.1	6,7	*GCK*-MODY															0,18	1	D	0,19	-33	N	N	-0,5	C65	0	89,28	-1,69	[43]	[2]
	T103K	7,5	1,3	0,4	25,6	0,9	5,6																0	1	D	0,5	38	N	N	-1,04	C65	0	77,74	-4,30	[43]	
	T103N	5,8	1,6	0,5	51,5	1,2	4,5	*GCK*-MODY		5,5	7,0												0	0,999	N	0,72	39	N	N	-0,73	C55	0	64,77	-4,65	[43]	[16]
	T103P	6	1,9	0,6	51,9	1,2	4,7																0	0,999	D	-0,08	62	N	D	-0,74	C35	0	37,56	-3,67	[43]	
	T103S	3,3	1,6	0,4	59,2	8,4	2,9	PHHI										8030	2,8				0	0,988	N	0,46	10	N	N	-0,64	C55	0	57,75	-3,52	[43]	[16,43]
																		9855	2,5																	
																		20440	3.1 - 4.0																	
	T103Y	9,1	1,3	0,4	39	0,9	5,5																0	1	D	0,76	36	N	N	-0,39	C65	0	92,25	-5,57	[43]	
	Q106P							*GCK*-MODY															0	0,805	D	1,48	31	D	D	-0,92	C15	53,64	65,86	-4,48		[2]
	Y108C	13,6	1,52	0,28	44	0,37	6,3	*GCK*-MODY															0,02	1	D	2,43	31	D	D	-1,67	C65	0	193,72	-4,12	[13]	[2]
	Y108F							*GCK*-MODY															0,25	0,996	D	0,57	13	N	N	-1,07	C15	0	21,61	-2,85		[2]
	Y108H							*GCK*-MODY	12	6,8	10,6												0	1	D	2,05	73	D	D	-2,02	C65	0	83,33	-5,18		[36]
									5	6,7	8,7																									
	I110N	14,87	1,37	0,03	3,07	0,02	7*	*GCK*-MODY		7,1													0	1	D	2,08	72	D	D	-1,21	C65	0	148,91	-6,59	[9]	[9]
										6.4																										
	I110T							*GCK*-MODY	38	6.6		280	6.6										0,08	0,999	D	1,74	2	N	D	-1,26	C65	0	89,28	-4,62		[44]
	P111L							*GCK*-MODY	33	7,0	6,7												0	0,975	D	2,29	35	D	D	-0,17	C65	0	97,78	-7,13		[36]
	E112K																						0,27	0,432	D	0,11	-65	N	D	-0,48	C0	121,33	35,46	-2,28		
	A114P							*GCK*-MODY															0	1	D	1,96	21	D	D	-0,58	C25	0	26,87	-4,76		[6]
	M115I							*GCK*-MODY															0,09	0,954	D	0,32	-28	N	D	0,12	C0	0	10,12	-4,38		[14]
	T116P							*GCK*-MODY															0,11	0,994	D	0,22	-29	N	D	-0,27	C35	0	37,56	-6,81		[2]
	G117R							*GCK*-MODY															0	1	D	0,92	58	D	D	-0,3	C65	0	125,13	-7,18		[45]
	G117S							*GCK*-MODY															0	1	D	1,17	5	N	D	-0,85	C55	0	55,27	-4,8		[2]
	T118I							*GCK*-MODY															0	0,999	D	1	20	D	D	-0,11	C65	0	89,28	-5,92		[2]
	A119D							*GCK*-MODY	29	7.4		1600	6										0,02	1	D	1,27	55	N	D	-1,14	C65	0	125,75	-5,42		[44]
	E120K							*GCK*-MODY															0,1	0,791	D	0,27	-51	N	D	-0,84	C0	56,87	0	-4,05		[7]
**Exon 4**																																				
	L122F							*GCK*-MODY															0	1	D	0,4	-1	D	D	-1,03	C15	0	21,82	-4,88		[2]
	L122P							*GCK*-MODY															0	1	D	2,85	72	D	D	-1,62	C65	0	97,78	-8,99		[2]
	F123L							*GCK*-MODY	6	5.6 - 6.9	< 7.8												0	0,999	D	1,74	62	D	D	-0,55	C15	0	21,82	-5,69		[32]
	F123S							*GCK*-MODY															0	1	D	3,03	71	D	D	-1,68	C65	0	154,81	-8,96		[21]
	D124H							*GCK*-MODY															0	1	D	0,36	36	D	D	-0,76	C65	0	81,24	-6,45		[2]
	D124N							*GCK*-MODY															0,03	1	D	0,34	7	D	D	-1,1	C15	0	23,01	-4,04		[2]
	Y125C							*GCK*-MODY															0	1	D	2,41	20	D	D	-1,45	C65	0	193,72	-7,62		[2]
	S127P							*GCK*-MODY															0	0,832	D	1,92	29	D	D	-1,41	C0	99,13	1,7	-4,11		[7]
	C129F							*GCK*-MODY															0	1	D	0,82	46	D	D	-0,71	C65	0	204,39	-6,83		[2]
	C129Y							*GCK*-MODY	26	5,0	10,5												0	1	D	0,72	41	D	D	-0,92	C65	0	193,72	-7,19		[46]
									7	9,9			6,4																							[8]
									34	7,6			5,5																							
									2				7																							
									28	6,7			6,4																							
									17	6,6			6,5																							
									60	7,1			6,9																							
									11	7,4			6,5																							
									21	7,7			6,8																							
									29	7,7			6,8																							
	I130N							*GCK*-MODY															0	1	D	3,41	42	D	D	-1,96	C65	0	148,91	-6,73		[2]
	I130T	8,59	1,01	0,13	2,88	0,17	7.1*	*GCK*-MODY	3,25	6,1	5,8		5,8										0	0,992	D	2,88	-27	N	D	-2,01	C65	0	89,28	-4,38	[47]	[47]
									3	6.4	9.2																									[36]
	S131P							*GCK*-MODY															0,01	1	D	1,39	-41	D	D	-1,15	C65	0	73,35	-7,45		[2]
	D132N							*GCK*-MODY	12	5.6 - 6.9	< 7.8		5.4										0,29	0,024	N	0,32	-87	N	N	-1,31	C15	0	23,01	-2,12		[32]
	F133L							*GCK*-MODY															0	0,675	D	1,76	34	D	D	-1,33	C0	21,61	21,82	-4,88		[45]
	L134P							*GCK*-MODY	43,4	7,1	14,0		6,3										0	1	D	2,99	49	D	D	-2,04	C65	0	97,78	-6,02		[48]
	H137D							*GCK*-MODY	6	≥ 7.0	< 7.8		5.6										0,09	0,367	D	1,92	-40	N	D	-0,78	C25	24,08	61,28	-4,81		[32]
	H137R	7,46	1,8	0,24	44,7	0,92		*GCK*-MODY															0,87	0,278	D	1,21	-85	N	N	-0,69	C0	24,08	28,37	-2,44	[17]	
		10,4	1,62	0,38	60,2	0,69	5,75																												[13]	
	Q138P							*GCK*-MODY		6,5	5,7												0,01	0,062	N	0,18	-63	N	N	-0,45	C15	45,75	58,16	-2,46		[16]
	M139L																						1	0	N	0,55	-94	N	N	-1,06	C0	13,4	0	-1,54		
	L144P							*GCK*-MODY	14	6,8	7,7	380	6,3										0	1	D	2,43	49	D	D	-2,03	C65	0	97,78	-6,53		[49]
									14	6,5	7,4	360	6,3																							
	P145S																						0,01	1	N	1,24	15	N	D	-1,48	C65	0	73,35	-3,94		
	L146P							PNDM						1	24	36,4	8,5						0	1	D	4,58	74	D	D	-2,39	C65	0	97,78	-8,85		[12]
	L146R	352	1,29	0,24	0,77	0,01		*GCK*-MODY															0	1	D	2,44	69	D	D	-2,24	C65	0	101,88	-8,85	[50]	[2]
		123	0,95	0,1	0,23	0,002	7																												[24]	
	F150L	20,05	1,27	2,15	13,2	0,13	6,9	*GCK*-MODY															0	1	D	1,27	52	D	D	-0,64	C15	0	21,82	-5,54	This study	
	F150S							*GCK*-MODY		6,7	9,5												0	1	D	4	70	D	D	-1,54	C65	0	154,81	-9,54		[51,52]
	F150Y							*GCK*-MODY															0	1	D	1,2	45	D	D	-0,9	C15	0	21,61	-7,48		[2]
	S151C							*GCK*-MODY															0	1	N	0,6	45	D	D	-0,6	C65	0	111,67	-5,8		[21]
	S151P							*GCK*-MODY															0	1	D	0,91	83	D	D	-0,58	C65	0	73,35	-4,2		[2]
	S151T							PNDM						42			8						0	0,988	D	0,78	44	D	D	-0,4	C55	0	57,75	-7,32		[12]
	F152I							*GCK*-MODY															0	1	D	1,1	56	D	D	-0,61	C15	0	21,28	-8,52		[2]

**F152L**							*GCK*-MODY													0	1	D	1,23	71	D	D	-0,64	C15	0	21,82	-7,26		[2]
**F152S**							*GCK*-MODY													0	1	D	3,59	77	D	D	-1,54	C65	0	154,81	-7,13		[34]
**V154L**							*GCK*-MODY													0,29	0,998	N	0,99	-75	N	N	-0,95	C25	0	31,78	-3,43		[11]
**R155T**							*GCK*-MODY	6	5,7											0,14	1	N	0,3	-83	N	N	-1,21	C65	0	70,97	-2,32		[53]
**H156D**							*GCK*-MODY													0	1	D	1,81	56	N	D	-1,47	C65	0	81,24	-6,01		[2]
**H156Y**							*GCK*-MODY	Infancy	7,0											0	1	D	0,42	39	N	D	-1,04	C65	0	83,33	-5,91		[36]
**E157K**							*GCK*-MODY													0,77	1	D	0,09	-84	N	D	-0,91	C55	0	56,87	-1,85		[2]
**D158A**	3,67	1,7	0,33	52,2	4,92															0,08	0,934	N	0,7	-52	N	N	-0,96	C65	0	125,75	-2,16	[17]	
	3,29	1,72	0,33	58,5	4,62	2,75																										[13]	
**I159N**							*GCK*-MODY													0	0,981	D	2,16	52	D	D	-1,68	C65	4,86	148,91	-7,14		[34]
**D160E**							*GCK*-MODY													0,06	0,996	N	0,83	-12	N	D	-1,42	C35	0	44,6	-5,23		[2]
**D160N**							*GCK*-MODY													0,04	1	N	-0,1	-67	N	N	-1,52	C15	0	23,01	-3,45		[2]
							PNDM		6,0				3287			8,1																	[12]
**K161N**							*GCK*-MODY													0,08	1	N	0,41	-87	N	D	-0,78	C65	0	93,88	-3,81		[2]
**G162D**							*GCK*-MODY	7	≥ 7.0	7.8 - 11.0		5,9								0	1	D	1,46	60	D	D	-1,41	C65	0	93,77	-5,96		[32]
**G162S**							*GCK*-MODY													0,15	1	D	0,59	-59	N	D	-1,34	C55	0	55,27	-2,92		[2]
**Exon 5**																																	
**L164F**							*GCK*-MODY													0	1	D	0,33	53	D	D	-1,05	C15	0	21,82	-7,87		[2]
**L164I**							*GCK*-MODY													0	0,998	D	0,62	-10	D	D	-1,1	C0	0	4,86	-5,25		[54]
**L164P**							*GCK*-MODY	35	7,4	16,1	343									0	1	D	0,96	84	D	D	-1,87	C65	0	97,78	-8,97		[55]
								61	6,7	12,1	379																						
								31	6,5	9,6	343																						
**L165F**	92,5	1,21	1,52	18	0,034	7.2 *	*GCK*-MODY	20	6.6	11.4	3500	6.7								0,01	0,994	D	0,81	26	N	D	-0,51	C15	0	21,82	-5,12	[56]	[56]
**L165V**							*GCK*-MODY													0,73	0,244	N	0,77	-95	N	N	-0,54	C25	0	31,78	-2,38		[14]
**W167F**	55,2	1,12		17,4																0	0,998	D	1,45	82	D	D	-0,96	C35	0	39,66	-7,17	[42]	
**W167R**							*GCK*-MODY													0,01	0,682	D	2,3	94	D	D	-0,92	C65	0	101,29	-7,45		[34]
**T168A**							*GCK*-MODY	8	5.6 - 6.9	< 7.8		6.2								0	0,999	D	1,88	71	D	D	-0,77	C55	0	58,02	-5,67		[32]
							PNDM						151	17,3		9,6																	[12]
**T168G**	26,7	1,01		5,7																0	1	D	2,07	70	D	D	-1,4	C55	0	59,16	-6,19	[42]	
**T168I**							*GCK*-MODY													0	1	D	0,49	85	D	D	0,22	C65	0	89,28	-8,16		[2]
**T168P**	160	0,92	9,33	0,35	0,0018		*GCK*-MODY													0	1	D	1,97	90	D	D	-0,38	C35	0	37,56	-9,30	[17]	[2]
	186	1	16,3	0,98	0	6,9																										[13]	
	27,16	1,03	1,58	0,93	0,058																											[18]	
**K169N**	28,2	0,98		0,02			*GCK*-MODY													0	1	D	0,04	80	D	D	-0,62	C65	0	93,88	-7,02	[42]	[2]
**K169R**							PNDM						11	39,8	96	7,9				0	1	D	0,35	72	D	D	-0,55	C25	0	26	-6,27		[12]
**G170D**							*GCK*-MODY	13												0	1	D	1,44	78	D	D	-0,56	C65	0	93,77	-3,76		[57]
								22																									
**G170S**							*GCK*-MODY													0	1	D	1,78	51	D	D	-0,78	C55	0	55,27	-4,22		[2]
**F171C**							*GCK*-MODY													0	1	D	2,33	37	D	D	-0,96	C65	0	204,39	-7,02		[2]
**F171L**							*GCK*-MODY													0	0,999	D	1,86	65	D	D	-0,3	C15	0	21,82	-5,14		[2]
**A173S**							*GCK*-MODY		6,3	7,3										0,09	0,997	D	0,99	14	N	D	-0,26	C65	0	99,13	-4,76		[16]
									6,5	7,5																							
									6,4	6,6																							
									6,1	11,2																							
								12	6,1	8,4																							[58]
								20	5,7	7,7																							
								16	5,7	8,0																							
								11	6,5	6,8																							
**A173V**																				0,04	1	D	0,58	-47	N	D	0,52	C55	0	64,43	-3,04		
**S174L**							*GCK*-MODY													0,01	1	D	0,86	13	D	D	0,94	C65	0	144,08	-6,23		[2]
**G175A**							*GCK*-MODY	57	6,0	9,5		6								0	0,999	D	1,65	29	D	D	0,23	C55	0	60	-6,24		[48]
**G175E**							*GCK*-MODY		6,2	7,8										0	1	D	1,91	49	D	D	-0,06	C65	0	97,85	-4,30		[16]
**G175R**	19,6	1,74	0,45	17,1	0,1		*GCK*-MODY													0	1	D	1,74	59	D	D	0,15	C65	0	125,13	-6,51	[17]	[2]
	38,6	1,37	0,5	34,3	0,13	6,9																										[13]	
**G175V**							*GCK*-MODY													0	1	D	2,26	17	D	D	0,42	C65	0	108,79	-9,04		[2]
**A176T**							*GCK*-MODY													0,04	1	N	0,79	-85	N	N	-0,67	C55	0	58,02	-3,53		[2]
**E177D**							*GCK*-MODY													0,1	0,965	D	-0,06	20	D	D	0,06	C35	0	44,6	-6,26		[59]
**G178A**							*GCK*-MODY													0	0,999	D	1,8	60	D	D	-0,5	C55	0	60	-5,82		[60]
**G178E**							*GCK*-MODY													0,01	1	D	1,99	73	D	D	-0,58	C65	0	97,85	-4,67		[2]
**G178R**							*GCK*-MODY													0	1	D	1,77	79	D	D	-0,36	C65	0	125,13	-4,2		[2]
**G178V**							*GCK*-MODY													0	1	D	2,32	76	D	D	-0,13	C65	0	108,79	-6,65		[2]
**G178W**							*GCK*-MODY													0	1	D	2,1	85	D	D	-0,36	C65	0	183,79	-7,6		[34]
**N180H**							*GCK*-MODY													0	1	D	0,5	44	N	D	-1,24	C65	0	68,35	-5,93		[61]
**N180K**							*GCK*-MODY		6.7	9.5										0	1	D	0,88	61	N	D	-1,12	C65	0	93,88	-5,49		[51.52]
**N180S**							*GCK*-MODY													0	0,996	D	0,41	19	N	D	-0,8	C45	0	46,24	-5,39		[2]
**V181A**							*GCK*-MODY		6,7	11,2										0	0,142	D	2,1	25	D	D	-2,07	C25	29,61	64,43	-3,83		[16]
**V182G**							*GCK*-MODY													0	1	D	2,07	10	D	D	-3,11	C65	0	108,79	-5,46		[2]
**V182L**	28,8	1,61	0,16	21.41 *	0.034 *	7.5 *	*GCK*-MODY	3	7,5			5,6								0	0,995	D	0,88	32	D	D	-1,24	C25	0	31,78	-6,15	[23]	[23]
								8	7,3																								
								10	7,1	9,5		6,8																					
								8	7,2			6,2																					
**V182M**	34,9	1,7	0,11	12,2	0,02		*GCK*-MODY													0	1	D	0,98	29	D	D	-1,38	C15	0	21,52	-6,84	[17]	[2]
	83,9	1,32	0,1	23,5	0,04	6,9																										[13]	
**L185P**							*GCK*-MODY													0	1	D	4,87	85	D	D	-2,79	C65	0	97,78	-9,48		[15]
**L185V**							*GCK*-MODY													0	0,997	D	1,43	37	D	D	-1,81	C25	0	31,78	-6,52		[6]
**R186L**							*GCK*-MODY													0	1	D	0,18	54	D	D	-0,6	C65	0	101,88	-5,7		[6]
**R186Q**							*GCK*-MODY													0	1	N	0,29	-20	N	N	-1,49	C35	0	42,81	-3,55		[2]
**D187Y**							*GCK*-MODY													0	1	D	0,16	71	D	D	-0,29	C65	0	159,94	-6,94		[2]
**A188E**	305	1	0,23	12,8	0,01	6,9	*GCK*-MODY													0	1	D	1,95	22	D	D	-1,53	C65	0	106,71	-5,96	[13]	[2]
**A188P**							*GCK*-MODY													0	1	D	2,46	56	D	D	-1,6	C25	0	26,87	-7,66		[2]
**A188T**							*GCK*-MODY	62	6,6	7,9										0,01	1	D	1,46	9	D	D	-1,45	C55	0	58,02	-6,18		[62]
								44	6,1	10,1																							
								55	8,3	11,5																							
								33	6,5	12,8																							
								13	6,3	12,2																							
**A188V**							*GCK*-MODY	5	6,1			6,1								0	1	D	0,94	7	D	D	-0,67	C55	0	64,43	-6,43		[8]
								6	5.3			6.9																					
**I189T**							*GCK*-MODY													0,01	1	D	2,51	37	D	D	-2,32	C65	0	89,28	-5,93		[2]
**R191L**							*GCK*-MODY													0	0,998	D	0,19	41	D	D	-0,16	C65	0	101,88	-6,91		[2]
**R191Q**							*GCK*-MODY		6.7	11.5										0	1	D	0,47	17	N	D	-1,03	C35	0	42,81	-6,25		[16]
								2,9	5,6	7,7		5,75																					[63]
								43	6,5	10,3																							
**R191W**							*GCK*-MODY	12	6,3			6,5								0	1	D	0,56	65	D	D	-0,46	C65	0	101,29	-9,83		[8]
**R192S**							*GCK*-MODY													0	1	D	0,58	22	N	D	-1,32	C65	0	109,21	-4,87		[20]
**G193R**							*GCK*-MODY		6,4	8,0										0,07	1	D	0,98	-64	D	D	-0,68	C65	0	125,13	-3,09		[16]
**G193W**							*GCK*-MODY													0	1	D	1,36	20	D	D	-0,79	C65	0	183,79	-7,52		[61]
**Exon 6**																																	
**M197I**	2,49	1,8	1,39	58,1	4,35	2,7	PHHI													0,81	0,972	N	0,26	-94	N	N	-1,31	C0	0	10,12	-1,03	[27]	[2]
**M197K**							*GCK*-MODY													0	0,988	D	2,72	64	D	D	-2,18	C65	0	94,49	-5,31		[2]
**M197T**							PHHI													0,03	0,993	D	1,79	-41	N	D	-1,66	C65	0	81,04	-2,94		[64]
**D198V**							*GCK*-MODY													0	1	D	-0,64	23	D	D	-0,14	C65	0	152,01	-4,9		[14]
**V199L**							*GCK*-MODY													0,04	0,995	D	0,45	14	N	D	-1,11	C25	0	31,78	-4,15		[2]
**V200A**	78,3	1,28	0,69	56,85	0,01	7 *	*GCK*-MODY		6,7											0,01	0,999	D	2,76	-7	N	N	-1,46	C55	0	64,43	-4,54	[9]	[9]
									6,9																								
									6,8																								
**V200L**							*GCK*-MODY													0,11	0,987	D	1,58	-40	N	D	-0,81	C25	0	31,78	-3,85		[2]
**A201P**							*GCK*-MODY													0	1	D	2,94	87	D	D	-1,61	C25	0	26,87	-8,42		[45]
**A201S**							*GCK*-MODY													0	0,999	D	1,13	41	D	D	-1,6	C65	0	99,13	-4,45		[2]
**M202R**							*GCK*-MODY	39	6.9			5.8								0	1	D	1,8	75	D	D	-1,88	C65	0	91,64	-5,77		[8]
**M202T**							*GCK*-MODY													0	0,999	D	2,3	55	D	D	-1,91	C65	0	81,04	-5,22		[34]
**V203A**	58,4	1,53	0,88	12,3	0,03		*GCK*-MODY	11	6.3	10.0	460	6.2								0	0,757	D	2,96	34	D	D	-2,16	C55	0	64,43	-4,97	[17]	[49]
	54,2	1,72	0,56	7,28	0,02	6,9																										[13]	
**N204A**	976	1,31		5,4																0	1	D	1,64	82	D	D	-1,13	C65	0	110,42	-8,30	[42]	
**N204D**	NA	NA	NA	NA			*GCK*-MODY													0	0,999	D	0,98	83	D	D	-1,17	C15	0	23,01	-5,79	[9]	
**D205H**	NA	NA	NA	NA			*GCK*-MODY	11,5	6,8	9,3		6,5								0	1	D	0,8	90	D	D	-0,46	C65	0	81,24	-7,25	[47]	[47]
**D205N**							*GCK*-MODY													0	1	D	0,77	83	D	D	-0,68	C15	0	23,01	-4,97		[2]
**D205Y**							*GCK*-MODY													0	1	D	0,87	91	D	D	-0,26	C65	0	159,94	-8,24		[2]
**V206A**							*GCK*-MODY													0,01	1	D	1,26	76	D	D	-1,43	C55	0	58,02	-4,2		[65]
**V206K**							*GCK*-MODY													0	1	D	1,86	91	D	D	-1,23	C65	0	77,74	-8,71		[2]
**T206M**	85	0,9	0,45	0,18	0,002	7.2 *	*GCK*-MODY	23	7,1	6,4	1500	6,3								0	1	D	0,27	87	D	D	-0,93	C65	0	81,04	-6,39	[56]	[16,56]
								20	6,8	12,2	2100	6,2																					
									6,4	11,1																							
								15	7,7	7,9	1700	5,5																					[49]
**T206P**							*GCK*-MODY													0	1	D	1,82	92	D	D	-1,24	C35	0	37,56	-7,39		[2]
**T206R**							*GCK*-MODY	8												0	1	D	1,09	91	D	D	-1,2	C65	0	70,97	-8,71		[33]
								34																									
								6																									
								4																									
**V207E**							*GCK*-MODY													0	1	D	2,96	88	D	D	-1,59	C65	0	121,33	-8,28		[2]
**V207L**							*GCK*-MODY													0	0,998	D	1,04	81	D	D	-0,85	C25	0	31,78	-5,09		[15]
**A208G**	5,66	1,38	0,43	21.79 *	2.17 *	4.4 *														1	1	N	1,26	-92	N	N	-1,88	C55	0	60	-0,33	[66]	
**A208P**							*GCK*-MODY													0	1	D	2,31	81	D	D	-1,34	C25	0	26,87	-7,08		[2]
**A208T**	10,1	1,23	2,61	2,86	0,12	6.9 *	PNDM						18	26,7		8,7				0	1	D	1,14	48	D	D	-1,31	C55	0	58,02	-4,54	[24]	[12]
**A208V**							*GCK*-MODY													0	1	D	0,17	64	D	D	-0,97	C55	0	64,43	-7,08		[2]
**T209K**	9,32	1,53	0,31	25,9	0,59	5,7	*GCK*-MODY													0	1	D	1,84	89	D	D	-1,24	C65	0	77,74	-6,76	This study	[38]
**T209M**							*GCK*-MODY													0	1	D	-0,02	79	D	D	-0,87	C65	0	81,04	-7,97		[2]
**T209R**							*GCK*-MODY													0	1	D	0,49	89	D	D	-1,14	C65	0	70,97	-6,85		[2]
**M210I**							*GCK*-MODY													0,02	0,994	D	0,97	16	D	D	-1,38	C0	0	10,12	-5,14		[2]
**M210K**	52,8	1,64	1,58	23,3	0,05		PNDM						1	8.1						0	1	D	2,31	76	D	D	-1,65	C65	0	94,49	-8,56	[24]	[67]
	31,8	1,37	0,42	13,4			*GCK*-MODY																									[24]	
	42,3	1,51	1	18,4	0,04	6,9																										[13]	
	38,7	1,63	1,38	16,7																												[67]	
**M210T**	234	1,36	5,68	6,22	0,004		*GCK*-MODY													0	0,999	D	2,61	56	D	D	-1,65	C65	0	81,04	-6,68	[17]	[2]
	127	1,24	0,41	32,9	0,04	6,9																										[13]	
**M210V**							*GCK*-MODY													0	0,997	D	1,53	27	D	D	-1,45	C15	0	21,52	-7,02		[2]
**I211F**							PHHI													0	1	D	0,89	-12	N	D	-1,36	C15	0	21,28	-5		[68]
**S212F**							*GCK*-MODY													0	1	N	0,26	49	D	D	-0,3	C65	0	154,81	-5,38		[2]
**S212P**							*GCK*-MODY													0	1	D	1,73	70	D	D	-0,63	C65	0	73,35	-6,91		[14]
**C213R**	58,4	1,76	0,85	14,1	0,03		*GCK*-MODY													0	1	D	0,69	-16	D	D	-0,79	C65	0	179,53	-4,20	[17]	[2]
	27,1	1,5	0,79	18,3	0,08	7																										[13]	
**C213Y**							*GCK*-MODY													0	1	D	0,13	50	D	D	-0,53	C65	0	193,72	-6,58		[2]
**Y214C**	1,87	1,32	0,29	53.33 *	26.64 *		PHHI										1	1,5	1	0,01	1	D	2,02	-29	N	D	-0,98	C65	0	193,72	-2,65	[66]	[69]
	1,35	1,55	1,08	65,3	17,3	1,4																										[27]	
	1,39	1,41	1,25	54,9	17,98	1,4																										[13]	
**Y214D**							*GCK*-MODY													0,01	0,999	D	2,21	53	N	D	-1,27	C65	0	159,94	-4,26		[14]
**Y215A**	2,2	1,33	0,65	26.15 *	9.26 *	2.05 *														0	1	D	2,9	80	D	D	-1,67	C65	0	111,59	-5,68	[66]	
**D217E**							*GCK*-MODY													0,32	0,999	N	1,53	-40	N	D	-0,75	C35	0	44,6	-3,3		[2]
**D217N**							*GCK*-MODY													0,29	1	N	0,35	-66	N	N	-1,06	C15	0	23,01	-3,48		[70]
**C220R**							*GCK*-MODY													0,01	0,073	D	2,55	64	D	D	-1,23	C65	0	179,35	-7,29		[7]
**C220Y**							*GCK*-MODY													0	0,998	D	1,39	67	D	D	-1,07	C65	0	193,72	-8,48		[71]
**E221K**							*GCK*-MODY, PNDM													0,13	1	N	0,47	-4	N	N	-1,16	C55	0	56,87	-4,25		[2]
**G223R**							*GCK*-MODY													0	1	D	0,43	92	D	D	-1,19	C65	0	125,13	-9,02		[2]
**G223S**	44	1,13	1,6	12,9	0,064	7.3 *	*GCK*-MODY	13	7,2	10,2		6,3								0	1	D	0,5	75	D	D	-1,53	C55	0	55,27	-4,86	[47]	[47]
	16,5	1,46	0,47	74,45	0,25	6.3 *			7,5																							[9]	[9]
									7,2																								
									6,8																								
								10	5,8			6,1																					[8]
								8	6,0	13,8		6,9																					[37]
**G223V**							*GCK*-MODY													0	1	D	-0,54	87	D	D	-1,01	C65	0	108,79	-9,02		[2]
**M224R**							*GCK*-MODY													0	0,999	D	1,94	62	N	D	-1,27	C45	14,3	90,56	-7,88		[2]
**M224T**							*GCK*-MODY													0	0,998	N	1,99	34	N	D	-1,3	C45	14,3	81,04	-5,36		[2]
**I225F**							*GCK*-MODY													0	1	D	1,12	74	D	D	-0,96	C15	0	21,28	-7,53		[2]
**I225M**							*GCK*-MODY													0	1	D	1,02	43	D	D	-0,87	C0	0	10,12	-7,15		[2]
**V226E**							*GCK*-MODY													0	1	D	4,19	62	D	D	-1,25	C65	0	121,33	-9,32		[38]
**V226L**							*GCK*-MODY													0,07	0,998	N	0,88	-75	N	N	-0,44	C25	0	31,78	-4,84		[20]
**V226M**	17,9	1,41	5,74	42,7	0,27		*GCK*-MODY	5	7.4			6.1								0	1	D	1,25	-28	D	D	-0,55	C15	0	21,52	-5,53	[17]	[8]
	32	1,1	2,23	26,6	0,4	6,7	PNDM						5479																			[13]	[12]
**G227C**							*GCK*-MODY													0	1	D	0,63	86	D	D	-1,02	C65	0	158,23	-7,1		[2]
**G227R**							*GCK*-MODY													0	1	D	1,35	93	D	D	-0,77	C65	0	125,13	-7,76		[2]
**G227S**							*GCK*-MODY													0	0,999	D	1,31	80	D	D	-1,12	C55	0	55,27	-4,77		[2]
**Exon 7**																																	
**T228A**	1,91	1,01	2,9	0,14	0.01 *	7.2 *	*GCK*-MODY													0	0,548	D	0,61	64	D	D	-0,69	C55	0	58,02	-5,46	[39]	[2]
**T228K**							*GCK*-MODY													0	0,991	D	0,72	85	D	D	-0,5	C65	0	77,74	-6,38		[2]
**T228M**				0,007	0,0001		*GCK*-MODY	13	7.4	13.4		6.8								0	1	D	0,64	69	D	D	-0,12	C65	0	81,04	-6,54	[50]	[37]
				< 0.02	0,0001		PNDM						1	40																		[17]	[67]
	5,5	0,71	0,62	0,004																												[67]	
**T228R**							*GCK*-MODY													0	1	D	0,57	84	D	D	-0,4	C65	0	70,97	-7,29		[2]
**G229D**							*GCK*-MODY													0,02	1	D	1,06	90	D	D	-0,71	C65	0	93,77	-8,7		[2]
**G229V**							*GCK*-MODY													0	1	D	-0,04	79	D	D	-0,19	C65	0	108,79	-6,06		[2]
**C230Y**							*GCK*-MODY													0	0,999	D	0,35	82	D	D	-0,09	C65	0	193,72	-6,56		[15]
**N231H**							*GCK*-MODY	6												0	1	D	0,21	76	D	D	-1,09	C65	0	68,35	-8,29		[8]
**N231S**							*GCK*-MODY													0	1	D	1,01	71	D	D	-1,1	C45	0	46,24	-5,98		[2]
**A232D**							*GCK*-MODY	60				6,9								0	1	D	2,62	89	D	D	-1,2	C65	0	125,75	-7,88		[72]
								59				6,5																					
								59				7																					
								30				6,9																					
								21				6,5																					
								33				6,1																					
								35				6,6																					
								35				6,4																					
								28				6,6																					
								29																									
								5				6,8																					
**C233R**	NA	NA	NA	NA			*GCK*-MODY	11	5,6	9,3										0	1	D	2,82	79	D	D	-1,03	C65	0	179,53	-8,00	[23]	[23]
								13	6,2	10,9																							
								24				5,1																					
								45																									

C233S							*GCK*-MODY									0	0,993	D	2,57	46	D	D	-1,28	C65	0	111,67	-4,33		[2]
Y234H							*GCK*-MODY									0	0,997	D	2,41	63	D	D	-1,28	C65	0	83,33	-9,17		[2]
M235R							*GCK*-MODY									0	0,053	D	1,27	74	D	D	-1,72	C65	0	91,64	-7,65		[2]
M235T	9,82	1,59	0,26	14,5	0,21	6,4	*GCK*-MODY									0	0,754	D	1,61	56	D	D	-1,78	C65	0	81,04	-6,44	[13]	[2]
M235V	7,8	1,38	0,46	22,9	0,45	6.2 *	*GCK*-MODY	27	6.8	8.2	699	6.1				0,21	0,809	N	1,08	-73	N	N	-1,55	C15	0	21,52	-4,16	[73]	[56]
E236K							*GCK*-MODY									0	0,919	D	0,91	75	D	D	-1,05	C55	0	56,87	-6,98		[2]
E237K							*GCK*-MODY									0,36	0,973	N	0,63	-63	N	N	-0,93	C55	0	56,87	-3,53		[2]
Q239R							*GCK*-MODY									1	0	N	0,16	-95	N	N	-0,19	C0	193,5	0	1,01		[2]
N240D							*GCK*-MODY									0,01	0,277	N	0,68	3	N	N	-0,96	C0	105,58	23,01	-4		[74]
N240I							*GCK*-MODY									0	0,938	D	1,29	14	D	D	-0,35	C0	105,58	59,51	-6,3		[2]
N240T							*GCK*-MODY									0,03	0,002	N	0,59	-24	N	D	-0,69	C0	105,58	0	-4,15		[2]
V241A							*GCK*-MODY									0,23	0,936	D	2,06	-23	N	D	-1,97	C55	0	64,43	-3,85		[2]
L243P							*GCK*-MODY									0,03	1	D	1,39	-77	D	D	-1,51	C65	0	97,78	-5,96		[20]
V244E							*GCK*-MODY									0	0,829	D	1,62	16	D	D	-2	C65	0	121,33	-5,71		[2]
V244G	12,77	1,52	0,4	75,71	0,43	5.9 *	*GCK*-MODY		7,2							0	0,953	D	1,74	5	D	D	-2,6	C65	0	108,79	-5,84	[9]	[9]
									6,7																				
									7,6																				
									6,6																				
									7,6																				
									7,1																				
									8,4																				
									6,0																				
									6,8																				
G246A							*GCK*-MODY									0,02	0,398	N	1,8	-2	N	D	-0,32	C55	0	60	-5,5		[21]
G246E							*GCK*-MODY									0,02	0,985	D	2,29	-11	N	D	-0,49	C65	0	97,85	-4,28		[2]
G246R							*GCK*-MODY									0,01	0,054	D	2,47	-1	N	D	-0,22	C65	0	125,13	-6,78		[2]
G246V							*GCK*-MODY									0	0,985	D	2,13	-2	D	D	-0,14	C65	0	108,79	-6,58		[14]
E248K							*GCK*-MODY									0,39	0,738	N	-0,09	-61	N	N	-0,23	C55	0	56,87	-3,99		[2]
G249A							*GCK*-MODY									0,02	0,943	N	1	-11	N	D	-0,37	C55	0	60	-6,09		[2]
G249C							*GCK*-MODY									0	1	D	0,99	4	D	D	-0,65	C65	0	158,23	-7,57		[7]
G249R							*GCK*-MODY									0,01	1	D	1,78	25	N	D	-0,27	C65	0	125,13	-4,31		[14]
G249S							*GCK*-MODY									0,02	0,992	D	1,26	-30	N	D	-0,71	C55	0	55,27	-4,3		[2]
R250C	7,94	1,57	0,45	36,1	0,94	5	*GCK*-MODY	5	7.4							0	1	D	1,31	32	D	D	-1,19	C65	0	179,53	-8,09	This study	[75]
R250H																0,36	0,999	N	0,58	-6	N	N	-1,35	C25	0	28,82	-3,67		
R250P							*GCK*-MODY									0,01	0,998	D	2	29	D	D	-1,13	C65	0	102,71	-5,28		[2]
M251C	55,33	1,38		3,3	< 0.01	≥ 7.1										0	1	D	1,4	49	D	D	-1,54	C65	0	195,55	-6,76	This study	
M251I	113,43	1,33		10,18	< 0.01	≥ 7.1	*GCK*-MODY		7,7							0	0,995	D	-0,05	40	D	D	-1,19	C0	0	10,12	-4,48	This study	[9]
									5,9																				
									6,6																				
									6,8																				
M251K							*GCK*-MODY									0	1	D	2,55	85	D	D	-1,5	C65	0	94,49	-6,56		[2]
M251R							*GCK*-MODY									0	1	D	1,72	84	D	D	-1,4	C65	0	91,64	-7,65		[15]
M251T							*GCK*-MODY									0	1	D	1,64	45	D	D	-1,54	C65	0	81,04	-5,13		[2]
M251V	46,4	1,59	0,51	7,6	0,01	7,1	*GCK*-MODY									0,22	0,995	D	0,42	-22	N	D	-1,11	C15	0	21,52	-3,22	This study	[2]
C252G							*GCK*-MODY									0	0,986	D	3,02	71	D	D	-1,97	C65	0	158,23	-6,12		[2]
C252R	8,37	1,61	0,34	11,8	0,27	6,2	*GCK*-MODY									0	1	D	1,75	77	D	D	-1,3	C65	0	179,53	-7,52	This study	[2]
C252W							*GCK*-MODY									0	1	D	0,4	65	D	D	-1,17	C65	0	214,36	-8,62		[15]
C252Y	24,5	1,85	0,56	13,2	0,02	6,9	*GCK*-MODY									0	1	D	0,63	75	D	D	-1,18	C65	0	193,72	-6,81	[13]	[2]
V253A							*GCK*-MODY									0	0,077	D	2,93	37	D	D	-1,81	C55	0	64,43	-8,45		[2]
V253F							*GCK*-MODY									0	1	D	0,97	50	D	D	-1,36	C45	0	49,94	-7,07		[74]
V253L							*GCK*-MODY									0	0,912	D	1,36	32	D	D	-1,06	C25	0	31,78	-4,78		[4]
N254H							*GCK*-MODY									0	1	D	0,25	79	D	D	-1,59	C65	0	68,35	-7,39		[2]
N254S							*GCK*-MODY									0	0,974	D	0,82	76	D	D	-1,57	C45	0	46,24	-5,1		[14]
T255A							*GCK*-MODY									0,03	0,948	D	1,18	26	D	D	-1,64	C55	0	58,02	-5,47		[2]
T255I							*GCK*-MODY									0,04	1	D	0,15	13	N	D	-0,64	C65	0	89,28	-4,84		[2]
T255N							*GCK*-MODY									0,01	0,999	D	1,63	79	D	D	-1,26	C55	0	64,77	-7,74		[14]
T255S							*GCK*-MODY									0,05	0,22	N	1,67	32	D	D	-1,13	C55	0	57,75	-5,27		[2]
E256A	1625	1,46		1,6												0	0,997	D	1,02	88	D	D	-1,25	C65	0	106,71	-7,65	[42]	
E256D							*GCK*-MODY									0	0,986	D	0,5	88	D	D	-1,03	C35	0	44,6	-5,71		[2]
E256K							*GCK*-MODY									0	1	D	0,9	92	D	D	-1,03	C55	0	56,87	-6,63		[2]
W257F	11,4	1,66		42,1												0	1	D	1,2	80	D	D	-1,1	C35	0	39,66	-6,46	[42]	
W257R							*GCK*-MODY	61	7,2							0	1	D	3,29	94	D	D	-1,23	C65	0	101,29	-7,95		[76]
								38	6,2																				
								74	6,7																				
								39	5,7																				
								48	7,5																				
								32	6,6																				
								16	5,5																				
								14	6,6																				
								6	6,7																				
								16	6,7																				
G258C							*GCK*-MODY									0	1	D	-0,37	61	D	D	-0,44	C65	0	158,23	-6,06		**[2]**
G258D							*GCK*-MODY	12	5,8	12,5	99,9					0	1	D	1,4	91	D	D	-0,83	C65	0	93,77	-5,93		[63]
								6,4	7,7	7,2	99,9	6.8																	
								36	6,8	10,0	166,5																		
G258R	NA	NA	NA	NA			*GCK*-MODY		7,3							0	1	D	0,66	87	D	D	-0,22	C65	0	125,13	-7,43	[9]	[9]
									6,3																				
									6,9																				
									6,6																				
									5,9																				
G258S							*GCK*-MODY									0	1	D	0,62	73	D	D	-0,78	C55	0	55,27	-5,58		[2]
A259T							*GCK*-MODY		6,5	7,5						0	0,998	D	0,36	6	N	D	-0,34	C55	0	58,02	-5,85		[51,52]
A259V							*GCK*-MODY									0	0,976	D	0,8	29	D	D	0,37	C55	0	64,43	-6,85		[2]
F260L	9,07	1,54	0,41	41,8	0,95	5,1										0,06	0,999	D	2,32	4	D	D	-1,53	C15	0	21,82	-4,03	This study	
F260S							*GCK*-MODY									0	1	D	4,01	77	D	D	-1,53	C65	0	154,81	-6,84		This study
G261E							*GCK*-MODY									0	1	D	3,31	73	D	D	-0,69	C65	0	97,85	-9,17		[2]
G261R	118; 130	2.2; 1.9	3,83	1.04; 1.51	0,00041		*GCK*-MODY		6,7	6,0						0	1	D	2,04	48	N	D	-0,49	C65	0	125,13	-5,52	[17]	[16]
	65,3	1,51	0,62	19,6	0,02	6,9		10	6.1	11.5																		[13]	[77]
									6,3	13,9																			
								0.02	8.9																				[10]
							PNDM						7	20	10.3														[12]
S263P	12,2	1,59	0,51	66,5	0,76		*GCK*-MODY	34								0	0,186	D	1,92	-35	D	D	0,07	C0	99,45	16,26	-4,02	[24]	20
	12,3	1,51	0,63	55,7				12																				[24]	
	12,3	1,55	0,57	61,1	0,64	6																						[13]	
G264S	9,76	1,57	0,48	63,5	0.94 *	5,6	*GCK*-MODY, PNDM									0,01	0,838	N	1,75	-45	D	D	-0,96	C0	55,27	0	-5,35	[78]	[2]
	9,76	1,57	0,48	60,7	0,95	5,4																						[13]	
E265K	15,5	1,43	0,63	36,8	0,4	6.4 *	*GCK*-MODY	14	8.0		1200	6.5				0,67	0,99	N	0,89	-90	N	N	-1,07	C55	0	56,87	-4,31	[56]	[56]
	11,5	1,37	0,4	48.59 *	0.55 *			8	7,2			7,2																[23]	[23]
								15	6,6																				
								19	7,8			6,5																	
								31	7,8			6,6																	
								12	5.6 - 6.9	≥ 11.1		6,5																	[32]
E265V							*GCK*-MODY									0,27	0,977	N	0,02	-93	N	N	-0,03	C65	0	121,33	-3,23		[2]
L266V							*GCK*-MODY									0	0,882	N	1,35	-47	N	N	-1,57	C25	0	31,78	-5,74		[2]
L266Q							*GCK*-MODY									0	1	D	2,55	0	D	D	-2,21	C65	0	112,44	-7,59		[2]
E268K							*GCK*-MODY									0,2	0,345	N	0,08	-19	N	N	-0,92	C15	44,6	56,87	-3,08		[2]
F269L							*GCK*-MODY									0,37	0,926	N	1,28	-66	N	N	-0,82	C15	0	21,82			[2]
Y273D							*GCK*-MODY									0	1	D	3,47	84	D	D	-2,03	C65	0	159,94	-8,04		[6]
Y273N							*GCK*-MODY									0	1	D	3,32	84	D	D	-1,89	C65	0	142,23	-8,04		[6]
D274E							*GCK*-MODY									0	0,803	D	0,57	83	D	D	-1,07	C35	0	44,6	-5,72		[2]
D274G							*GCK*-MODY									0	0,984	D	1,3	89	D	D	-1,59	C65	0	93,77	-8,12		[2]
D274H							*GCK*-MODY									0	0,46	D	0,52	88	D	D	-1,25	C65	0	81,24	-8,12		[34]
D274N							*GCK*-MODY									0	0,975	D	0,77	84	D	D	-1,27	C15	0	23,01	-8,12		[6]
R275C	7,81	1,66	0,46	77,2	1,1	5	*GCK*-MODY	12	6,6	8,1						0	0,999	D	1,1	-19	D	D	-0,9	C45	28,82	169,74	-6,45	[13]	[58]
								6	5,9	6,7																			
R275H							*GCK*-MODY									0,03	0,011	N	0,64	-80	N	N	-1,14	C0	28,82	0	-4,7		[79]
R275L							*GCK*-MODY									0,03	0,616	D	0,14	-65	D	D	-0,42	C35	28,82	97,52	-4		[15]
L276P							*GCK*-MODY									0,18	0,243	D	1,79	-72	N	D	-1,82	C25	31,78	67,79	-3,29		[2]
L276V																0,35	0	N	0,6	-97	N	N	-1,33	C0	31,78	0	2,11		
V277R							*GCK*-MODY									0	0,99	D	1,33	27	D	D	-1,91	C35	29,61	87,8	-8,95		[38]
D278E							*GCK*-MODY									0	0,981	D	0,23	46	D	D	-0,51	C35	0	44,6	-6,49		[2]
E279G							*GCK*-MODY									0,01	0,94	D	0,94	-45	N	N	-1	C65	0	97,85	-5,06		[2]
E279Q							*GCK*-MODY									0,22	0,941	N	-0,08	32	N	N	-0,47	C25	0	29,27	-3,83		[2]
S281F							*GCK*-MODY									0	1	D	0,76	52	D	D	0,13	C65	0	154,81	-7,93		[2]
S281P							*GCK*-MODY									0	0,084	D	1,49	65	D	D	-0,28	C65	0	73,35	-9,29		[15]
Q287K							*GCK*-MODY									0,01	0,997	D	1,18	63	D	D	-0,51	C45	0	53,23	-5,64		[2]
Q287L							*GCK*-MODY									0	0,998	D	0,47	57	D	D	0,23	C65	0	112,44	-6,58		[2]
Q287P							*GCK*-MODY									0	0,998	D	1,78	63	D	D	-0,43	C65	0	75,14	-6,9		[80]
Q287V	35,1	1,22		35												0	0,998	D	1,2	38	D	D	0,05	C65	0	96,33	-6,80	[42]	
**Exon 8**																													
Y289C							*GCK*-MODY									0,01	0,342	D	3,19	42	D	D	-1,1	C65	0	193,72	-5,84		[2]
Y289H							*GCK*-MODY									0,04	0,977	D	3,03	10	D	D	-1,22	C65	0	83,33	-4,13		[2]
Y289N							*GCK*-MODY									0	0,959	D	4,44	76	D	D	-1,29	C65	0	142,23	-6,8		[2]
E290A	829	1,03		27,7												0	0,974	D	1,3	70	D	D	-0,79	C65	0	106,71	-7,52	[42]	
E290D							*GCK*-MODY									0,02	0,918	D	1,11	49	D	D	-0,69	C35	0	44,6	-3,83		[2]
K291E							*GCK*-MODY									0,01	0,126	D	1,31	89	D	D	-0,78	C55	0	56,87	-6,75		[2]
K291T							*GCK*-MODY									0	0,958	D	2	79	D	D	-0,67	C65	0	77,74	-6,59		[74]
L292P							*GCK*-MODY									0	0,999	D	3,43	71	D	D	-1,74	C45	14,3	86,59	-5,86		[20]
I293T							*GCK*-MODY									0,1	0,893	D	2,33	-46	N	D	-1,62	C65	0	89,28	-3,01		[81]
G294D							*GCK*-MODY									0	1	D	0,87	82	D	D	-1,32	C15	55,27	65,41	-7,39		[2]
G295C							*GCK*-MODY									0	0,999	D	0,08	82	D	D	-0,66	C65	0	158,23	-8,43		[45]
G295D					< 0.01	≥ 7.1	*GCK*-MODY									0,01	0,042	D	0,52	92	D	D	-0,86	C65	0	93,77	-7,22	This study	[38]
G295S							*GCK*-MODY									0,02	0,766	D	-0,19	78	D	D	-0,69	C55	0	55,27	-4,43		[2]
M298K	15,9	1,25	3,7	24,6	0,44	6,5	*GCK*-MODY									0	0,157	D	2,84	74	D	D	-1,9	C65	0	94,49	-8,22	[13]	[2]
M298T							*GCK*-MODY									0	0,605	D	2,39	48	D	D	-1,69	C65	0	81,04	-7,06		[2]
M298V							*GCK*-MODY									0	0,809	D	1,08	-19	D	D	-1,36	C15	0	21,52	-4,65		[2]
G299D							*GCK*-MODY									0	1	D	1,23	83	D	D	-1,65	C65	0	93,77	-8,47		[2]
G299R							*GCK*-MODY									0	1	D	0,34	88	D	D	-1,14	C65	0	125,13	-6,17		[2]
G299V							*GCK*-MODY									0	1	D	-0,04	78	D	D	-0,51	C65	0	108,79	-8,47		[2]
E300K	10,3	1,85	0,46	33,8	0,55		*GCK*-MODY									0	0,991	D	1,18	72	D	D	-1,67	C55	0	56,87	-5,90	[17]	[2]
	9,17	1,65	0,7	59,5	0,66	5,8																						[13]	
E300Q							*GCK*-MODY									0	0,986	D	0,65	45	D	D	-1,32	C25	0	29,27	-8,23		[2]
V302L							*GCK*-MODY									0	0,001	N	0,94	-14	N	D	-1,66	C0	64,43	31,78	-4,45		[20]
V302M							*GCK*-MODY									0	0,952	D	0,87	-15	D	D	-1,83	C0	64,43	21,52	-5,54		[2]
R303L							*GCK*-MODY									0	0,343	D	0,84	84	D	D	-0,9	C65	0	101,88	-6,54		[82]
R303W							*GCK*-MODY	1.5	7.2-8.3	8,6		6.6				0	1	D	0,74	90	D	D	-1,27	C65	0	101,29	-5,56		[63]
								17	7,1	12,8		6,1																	
L304P							*GCK*-MODY									0,11	0,999	D	2,48	-8	D	D	-2,42	C65	0	97,78	-7,91		[2]
L306P							*GCK*-MODY									0	1	D	3,84	78	D	D	-2,16	C65	0	97,78	-8,49		[45]
L306R							*GCK*-MODY									0	1	D	1,9	71	D	D	-2,03	C65	0	101,88	-8,49		[2]
L307F							*GCK*-MODY									0,01	0,718	N	1,23	-16	N	D	-1,34	C0	14,3	21,28	-4,84		[6]
R308K							*GCK*-MODY									1	0	N	0,67	-97	N	N	-1,27	C0	26	0	3,23		[83]
R308W	10,7	1,36	0,5	22,3	0,29	6.7 *	*GCK*-MODY	17	7.5	12.6	273	5.4				0,01	0,835	D	0,7	47	D	D	-0,85	C35	26	95,78	-4,78	[73]	[56]
L309H							*GCK*-MODY									0	1	D	1,97	31	D	D	-2,47	C45	14,3	86,34	-6,62		[2]
L309P	9,46	1,92	0,58	1,1	0,03		*GCK*-MODY									0,01	0,643	D	3,55	51	D	D	-2,47	C45	14,3	86,59	-7,25	[17]	[2]
	12,5	1,23	0,62	2,46	0,06	6,9																						[13]	
E312Q							Gestational prediabetes	32	3,3	7,8						0,57	0,957	N	-0,31	-92	N	N	-0,55	C25	0	29,27	-2,39		[46]
L314P	13,63	1,4	0,3	5,9	0,11	6,9	*GCK*-MODY									0	0,068	D	2,44	27	D	D	-2,12	C65	0	97,78	-6,58	This study	[2]
L315F							*GCK*-MODY	9	6,5			7,9				0	0,987	N	0,27	41	D	D	-1,75	C0	31,78	21,28	-5,79		[8]
L315H	8,09	1,67	0,5	69,21	0,89	7	*GCK*-MODY		11,8							0	1	D	0,98	76	D	D	-2,7	C25	31,78	83,01	-7,98	[9]	[9]
									7,5																				
									7,1																				
									9,8																				
									7,4																				
L315Q							*GCK*-MODY									0	1	D	1,67	64	D	D	-2,63	C35	31,78	96,32	-8,35		[14]
F316V	11,2	1,66	0,69	45,5	0,51	5,7	*GCK*-MODY									0	0,848	D	2,22	44	D	D	-1,59	C45	0	49,94	-7,29	This study	[2]
G318A							*GCK*-MODY									0	0,006	D	1,75	30	N	D	-0,7	C55	0	60	-4,18		[2]
G318R	8,53	1,63	0,53	34	0,67	5,3	*GCK*-MODY		5,9							0	0,998	D	1,23	64	N	D	-0,69	C65	0	125,13	-5,64	This study	[9]
									6,6																				
									6,8																				
									6,1																				
									6,5																				
									7,1																				
									5,9																				
								16	5,6	5,6																			[36]
E319Q							*GCK*-MODY									1	0,001	N	-0,32	-93	N	N	-0,35	C0	61,28	0	-2,32		[2]
L324P							*GCK*-MODY									0	1	D	3,23	66	D	D	-2,1	C65	0	97,78	-8,42		[2]
T326P							*GCK*-MODY									0	0,001	D	2,55	-32	D	D	-0,33	C35	0	37,56	-5,83		[2]
R327H							*GCK*-MODY									0	0,961	D	0,04	-48	N	D	-0,66	C0	26	23,36	-3,08		[2]
E331K							*GCK*-MODY									0,31	0,025	D	-0,01	29	N	N	-0,57	C55	0	56,87	-3,72		[2]
T332K							*GCK*-MODY	2	6.7							0	0,994	D	1,56	77	D	D	-1,26	C15	57,75	72,73	-8,57		[53]
S336L	5,42	1,13	21,7	1,03	0,07		*GCK*-MODY									0	0,999	D	0,68	35	D	D	-0,6	C65	0	144,08	-3,75	[17]	[2]
	7,81	0,85	9,5	0,98	0,06	6,9																						[13]	
	6	1,02	15,2	1,28	0,008																							[39]	
S336W							*GCK*-MODY									0	1	D	0,63	57	D	D	-0,79	C65	0	176,58	-5,5		[2]
Q337P							*GCK*-MODY									0,01	0,825	D	1,72	-33	N	D	-1,16	C65	0	75,14	-5,65		[2]
E339G	32,1	1,34	1,87	34,9	0,06		*GCK*-MODY									0	0,999	D	1,8	29	D	D	-2,02	C65	0	97,85	-5,07	[24]	[2]
	20,5	1,53	2,47	41,7																								[24]	
	26,3	1,44	2,17	38,3	0,19	6,8																						[13]	
E339K							*GCK*-MODY									0	0,996	D	0,77	41	D	D	-1,66	C55	0	56,87	-5,91		[84]
S340I							*GCK*-MODY									0	0,98	N	0,74	-26	N	N	-0,46	C15	103,39	92,79	-4,75		[2]
S340T							*GCK*-MODY									0,04	0,001	N	0,01	-90	N	N	-0,53	C0	103,39	29,38	-2,77		[2]
**Exon 9**																													
T342P							Benign	43	6.0	8.5						0,47	0	D	0,43	-82	N	N	-0,48	C0	79,02	0	0,00		[36.85]
D344Y							*GCK*-MODY									0,02	1	D	0,34	5	N	D	-0,54	C65	0	159,94	-5,26		[86]
I348F							*GCK*-MODY									0	0,998	D	0,88	-13	N	D	-1,9	C0	89,28	21,28	-4,93		[87]
I348N							*GCK*-MODY									0	1	D	2,14	-27	D	D	-2,59	C0	89,28	64,77	-6,02		[14]
L352R							*GCK*-MODY									0	1	D	2,43	65	D	D	-2,5	C65	0	101,88	-7,5		[61]

**G356V**							*GCK*-MODY															0	0,994	D	2,56	1	N	D	-0,06	C0	97,85	71,42	-5,06		[2]
**P359L**							*GCK*-MODY															0,41	0,927	N	0,39	-77	N	N	-0,81	C35	26,87	94,04	-4,29		[2]
**D363N**							*GCK*-MODY															0	0,987	D	0,89	67	D	D	-1,75	C15	0	23,01	-2,14		[2]
**D363V**							*GCK*-MODY															0	0,992	D	-0,54	56	D	D	-0,54	C65	0	152,01	-1,32		[2]
**D365N**							*GCK*-MODY															0,26	0,96	N	0,36	-95	N	N	-1,43	C0	81,24	0	-3,69		[4]
**V367M**	7,56	1,8	0,31	58,1	1,26		*GCK*-MODY															0	1	D	0,96	-53	N	D	-1,97	C15	0	21,52	-4,17	[17]	[2]
	8,39	1,68	0,45	85	1,4	4,75																												[13]	
	6,93	1,55	0,51	109,74	1,26																													[18]	
**R369C**							*GCK*-MODY	45,2	6,7	13,8		6										0,15	1	D	1,49	-24	D	D	-1,68	C25	102,84	152,85	-3,94		[48]
**R369P**							*GCK*-MODY															0,17	0,943	D	1,78	7	D	D	-1,71	C0	102,84	73,46	-4,83		[2]
**C371F**							*GCK*-MODY															0	1	D	0,1	65	D	D	-1,18	C65	0	204,39	-6,72		[2]
**C371R**							*GCK*-MODY															0	1	D	1,37	73	D	D	-1,58	C65	0	179,53	-7,83		[2]
**C371W**							*GCK*-MODY															0	1	D	0,67	76	D	D	-1,42	C65	0	214,36	-7,51		[2]
**C371Y**							*GCK*-MODY															0	1	D	0,18	66	D	D	-1,42	C65	0	193,72	-7,61		[2]
**S373N**							*GCK*-MODY															0,03	0,235	N	1,13	-26	N	N	-1,08	C0	135,7	17,8	-5,38		[2]
**S373T**																						0,24	0,235	N	0,55	-94	N	N	-1,07	C0	135,7	6,47	-1,08		
**V374E**							*GCK*-MODY															0	1	D	3,02	74	D	D	-2,43	C65	0	121,33	-7,65		[2]
**V374M**							*GCK*-MODY															0	1	D	1,35	39	D	D	-1,68	C15	0	21,52	-5,84		[2]
**S375P**							*GCK*-MODY															0	1	D	1,71	80	D	D	-1,47	C65	0	73,35	-8,69		[2]
**R377C**	52,3	1,64	0,28	5,87	0,01		*GCK*-MODY															0	1	D	1,65	71	D	D	-1,22	C65	0	179,53	-7,29	[24]	[2]
	33,2	1,85	0,45	9,11																														[24]	
	42,8	1,75	0,37	7,49	0,01	6,9																												[13]	
**R377H**							*GCK*-MODY															0,01	1	D	1,28	90	D	D	-1,34	C25	0	28,82	-7,07		[2]
**R377L**							*GCK*-MODY															0	1	D	0,04	90	D	D	-0,61	C65	0	101,88	-7,29		[2]
**R377S**							*GCK*-MODY															0	1	D	2,53	85	D	D	-1,38	C65	0	109,21	-6,54		[2]
**A378D**							*GCK*-MODY															0	1	D	2,38	88	D	D	-1,49	C65	0	125,75	-8,2		[2]
**A378G**							*GCK*-MODY															0	0,859	N	1,77	65	N	D	-2,08	C55	0	60	-3,95		[2]
**A378P**							*GCK*-MODY															0,01	1	D	2,44	90	D	D	-1,24	C25	0	26,87	-7,44		[20]
**A378T**							*GCK*-MODY	7	6,1			6,3										0	1	D	1,5	68	D	D	-1,27	C55	0	58,02	-4,71		[8]
								15	7,3			6,4																							
								22	6,1			6,5																							
								52	7,2			6,4																							
								3	11,4			6,2																							
**A378V**	576	0,94	9,92	55,4	0.018 *	6,9	PNDM						1	13.4 - 24.6								0	0,998	D	0,22	79	D	D	-0,54	C55	0	64,43	-5,63	[78]	[78]
	584	0,93	8,08	41	0,03	6,9	*GCK*-MODY		6.9			5.5																						[13]	
									8.6			7.6																							
**A379E**							*GCK*-MODY															0	1	D	1,41	90	D	D	-1,24	C65	0	106,71	-6,81		[2]
**A379V**	13,7	1,45	0,69	36.79 *	0.24 *	6.7 *	*GCK*-MODY	30	7,4	12,1												0	0,999	D	-0,14	75	D	D	-0,48	C55	0	64,43	-5,57	[23]	[23]
								13	5,7	12,5		5,7																							
								56	7,6																										
**H380D**							*GCK*-MODY															0,04	0,175	D	0,71	-6	N	D	-1,02	C0	108,82	60,77	-3,68		[2]
**H380P**							*GCK*-MODY															0,02	0,511	D	1,81	52	D	D	-0,55	C0	108,82	52,18	-4,6		[2]
**H380Q**							*GCK*-MODY	10	6,0	8,5												1	0,001	N	-0,01	-89	N	N	-0,85	C0	108,82	0	-0,56		[58]
								10	6,1	8,6																									
**M381R**							*GCK*-MODY															0	0,997	D	1,75	58	D	D	-1,46	C45	14,3	90,56	-6,61		[45]
**M381T**							*GCK*-MODY															0	0,8	D	2,23	51	N	D	-1,48	C45	14,3	81,04	-4,46		[2]
**C382G**							*GCK*-MODY															0	1	D	2,44	56	D	D	-1,84	C65	0	158,23	-5,07		[2]
**C382R**							*GCK*-MODY															0	1	D	1,67	64	D	D	-1,07	C65	0	179,53	-6,99		[61]
**C382Y**							*GCK*-MODY		5.7	7.9												0,01	1	D	0,38	53	D	D	-0,93	C65	0	193,72	-4,87		[51,52]
**S383L**							*GCK*-MODY	8	6,8	9,7	420	6,6										0	0,999	D	-0,19	11	D	D	0,07	C15	103,39	95,33	-6,14		[49]
**S383P**							*GCK*-MODY															0	0,988	D	2,43	68	D	D	-1,1	C0	103,39	1,7	-4,01		[6]
**S383T**							*GCK*-MODY															0,04	0,583	N	0,74	-66	N	D	-0,88	C0	103,39	29,38	-2,65		[2]
**A384G**							*GCK*-MODY															0	0,966	D	2,32	27	D	D	-2,28	C55	0	60	-5,43		[2]
**A384T**							*GCK*-MODY		7.1	7.2												0,01	1	D	1,65	-34	D	D	-1,57	C55	0	58,02	-3,51		[51,52]
**A384V**							*GCK*-MODY															0,04	1	D	1,02	-28	D	D	-0,88	C55	0	64,43	-3,67		[74]
**G385R**							*GCK*-MODY															0	1	D	0,61	76	D	D	-1,06	C65	0	125,13	-8,31		[2]
**G385V**							*GCK*-MODY	18	7,2			6,7										0,02	1	D	-1,14	61	D	D	-0,66	C65	0	108,79	-4,99		[44]
**G385W**					< 0.01	≥ 7.1	*GCK*-MODY															0	1	D	-1,11	82	D	D	-0,9	C65	0	183,79	-8,58	This study	
**L386V**							*GCK*-MODY															0,31	0,999	N	1,54	-96	N	N	-1,46	C25	0	31,78	-2,62		[2]
**A387E**							*GCK*-MODY															0	1	D	2,24	42	D	D	-1,46	C55	0	106,71	-7,06		[2]
**A387T**							*GCK*-MODY	28	5.5	7.3												0,01	1	D	1,62	-24	D	D	-1,38	C55	0	58,02	-4,87		[36]
**A387V**							*GCK*-MODY															0,01	1	D	0,44	-45	N	D	-0,92	C55	0	64,43	-3,64		[2]
**G388D**							*GCK*-MODY															0	1	D	1,07	49	D	D	-1,56	C65	0	93,77	-4,83		[15]
**V389A**							*GCK*-MODY															0	0,963	D	2,84	-42	D	D	-1,83	C25	29,61	64,43	-5,34		[2]
**V389C**	8,3	1,5	0,5	58,5	0,8	5																0	1	D	1,32	-57	D	D	-1,5	C45	29,61	191,12	-5,93	[43]	
**V389D**	78,6	1,5	1,5	10,3	< 0.1	7,1	*GCK*-MODY															0	1	D	3,8	22	D	D	-1,7	C45	29,61	152,01	-7,73	[43]	[2]
**V389L**	3,5	1,6	0,5	67,8	6	2,9	PHHI										1314	2,9				0,51	0,838	N	0,57	-95	N	N	-0,91	C0	29,61	4,86	-1,47	[43]	[43]
**V389P**	39,4	1,8	0,6	31,3	< 0.1	6,8																0	1	D	3,5	-6	D	D	-1,6	C25	29,61	67,79	-5,14	[43]	
**V389R**	68,8	1,6	0,5	42,3	< 0.1	6,8																0	1	D	1,85	0	D	D	-1,48	C35	29,61	87,8	-7,73	[43]	
**V389S**	23,9	1,3	0,7	39,9	< 0.1	6,5																0	1	D	3,22	-26	D	D	-1,7	C35	29,61	123,03	-5,22	[43]	
**R392C**							*GCK*-MODY															0	1	D	1,72	6	D	D	-1,1	C25	104,93	150,08	-5,14		[2]
**R392S**							*GCK*-MODY	8	5.6 - 6.9	< 7.8		6.7										0,01	0,312	D	2,09	-49	N	D	-1,22	C15	104,93	83,67	-5,05		[32]
**M393T**							PNDM						21	22,5	125,8							0	0,881	D	2,22	-38	N	N	-1,36	C65	0	81,04	-3,78		[12]
**R397C**							*GCK*-MODY															0	1	D	1,11	24	D	D	-1,27	C65	0	179,53			[2]
**R397L**	7,7	1,5	0,46	42,1	0,72	5.5 *	*GCK*-MODY	15	6.5	8.6	500	6.5										0	0,998	D	0,46	31	D	D	-0,81	C65	0	101,88		[73]	[56]
							PNDM						24	21		9,7																			[12]
**I404S**							*GCK*-MODY															0,06	1	D	4,2	8	N	D	-1,95	C65	0	141,8	-3,85		[2]
**T405I**							*GCK*-MODY															0	0,993	D	0,17	41	N	D	-0,97	C65	0	89,28	-3,66		[2]
**V406A**							*GCK*-MODY															0	0,934	D	3,41	53	D	D	-1,94	C55	0	64,43	-4,49		[6]
**V406M**							*GCK*-MODY															0	0,803	D	1,57	66	D	D	-1,09	C15	0	21,52	-5,59		[2]
**G407D**							*GCK*-MODY															0	1	D	2,03	87	D	D	-1,43	C65	0	93,77	-8,99		[2]
**G407S**							*GCK*-MODY	4	6,4	7,4												0	1	D	0,07	66	D	D	-1,32	C55	0	55,27	-5,45		[58]
**S411A**	6,13	1,62	0,33	20,13	0.26 *	6.2 *																0,02	0,179	N	0,25	20	N	D	-0,55	C65	0	99,13	-3,91	[39]	
**S411F**	3,61	1	9,58	0,09	0	6,9	*GCK*-MODY															0	0,997	D	0,94	84	D	D	0	C65	0	154,81	-5,57	[13]	[2]
**S411L**	4,14	1,73	0,49	2,67	0.05 *	6.9 *																0	0,973	D	0,76	84	D	D	-0,09	C65	0	144,08	-7,47	[39]	
**V412A**							*GCK*-MODY															0	0,999	D	1,9	20	D	D	-1,55	C55	0	64,43	-4,85		[2]
**V412M**							*GCK*-MODY															0	1	D	0,67	-21	D	D	-0,96	C15	0	21,52	-4,52		[2]
**Y413C**							*GCK*-MODY															0	1	D	2,82	67	D	D	-1,58	C65	0	193,72	-7,92		[2]
**K414E**	12,3	1,67	1,73	11	0,32		*GCK*-MODY															0	0,999	D	1,12	62	N	N	-1,04	C55	0	56,87	-4,49	[17]	[2]
	7,17	1,81	0,93	36,2	0,62	5,3																												[13]	
**L415V**							*GCK*-MODY															0,02	0,984	N	0,16	-50	N	D	-0,76	C25	0	31,78	-7,12		[2]
**H416D**							*GCK*-MODY															0	1	D	1,7	75	D	D	-1,28	C65	0	81,24	-8,49		[2]
**H416P**							*GCK*-MODY															0	1	D	2,21	83	D	D	-1,02	C65	0	76,28	-8,49		[2]
**H416R**	67,9	1	3,74	1,02	0,003	7.4 *	*GCK*-MODY	1,9	6,3	11,1		5,7										0	1	D	1,64	73	D	D	-1,1	C25	0	28,82	-6,71	[47]	[47]
**Exon 10**																																			
**F419C**							*GCK*-MODY															0	1	D	2,72	39	D	D	-1,66	C65	0	204,39	-6,24		[21]
**F419L**	15	1,67	0,43	25,4	0,19	6,6	*GCK*-MODY															0	0,999	D	1,63	58	D	D	-1,22	C15	0	21,82	-3,96	This study	[2]
**F419S**	161,5	1,12	6,14	82,1	0,01	7 *	*GCK*-MODY		7,1													0	1	D	3,08	80	D	D	-1,77	C65	0	154,81	-7,40	[9]	[9]
									7,8																										
									6,7																										
**F419V**							*GCK*-MODY															0	1	D	2,07	52	D	D	-1,49	C45	0	49,94	-5,06		[2]
**K420E**	11,6	1,47	0,41	52.95 *	0.46 *	6.1 *	*GCK*-MODY	5	6,4	10,2		6,4										0,13	0,52	D	1	-60	N	N	-0,89	C0	53,23	16,44	-3,14	[23]	[23]
								12	6,7	10,1		6																							
								68	6,8	10,5																									
								15	6,0	10,9																									
**R422G**							*GCK*-MODY															0,05	0,995	D	2,35	6	D	D	-1,58	C25	38,73	97,11	-4,78		[6]
**R422L**							*GCK*-MODY															1	0,987	D	0,15	-45	N	D	-0,75	C25	38,73	91,85	-2,38		[3]
**F423S**							*GCK*-MODY															0	1	N	3,17	32	N	D	-2,09	C65	0	154,81	-4,12		[45]
**F423Y**							*GCK*-MODY															0	1	N	0,55	-12	N	N	-1,46	C15	0	21,61	-3,77		[2]
**H424Y**							*GCK*-MODY	15	6.8													0,03	1	N	0,19	11	N	D	-0,51	C65	0	83,33	-4,58		[53]
**R428C**																						0	1	D	0,93	7	D	D	-1,04	C15	125,81	150,08	-5,75		
**R428P**							*GCK*-MODY															0	0,998	D	1,26	27	D	D	-1,04	C0	125,81	64,34	-5,43		[20]
**R429K**																						0,32	0	N	-0,02	-94	N	N	-0,77	C0	64,93	0	-0,23		
**T431A**																						1	0,002	N	1,12	-89	N	N	-2,01	C0	58,02	0	-1,35		
**C434F**							*GCK*-MODY	46	6.4			6.3										0,01	0,999	D	0,62	21	D	D	-0,52	C65	0	204,39	-5,03		[8]
**C434T**							*GCK*-MODY															0,01	0,944	N	1,7	-9	N	N	-0,86	C65	0	148,93	-3,98		[2]
**C434W**							*GCK*-MODY															0	1	N	0,9	32	D	D	-0,87	C65	0	214,36	-8,09		[2]
**C434Y**	7,36	1,66	0,45	29	0,71	5,1	*GCK*-MODY															0,05	1	D	0,84	2	D	D	-0,91	C65	0	193,72	-3,81	This study	[38]
**I436F**							*GCK*-MODY															0,41	1	D	0,96	-86	N	N	-1,74	C15	0	21,28	-2,54		[2]
**I436N**	7,84	1,66	0,5	73,77	1,07	7	*GCK*-MODY		6,7													0	1	D	3,44	52	D	D	-2,49	C65	0	148,91	-7,34	[9]	[9]
									6,2																										
									7,9																										
									7,2																										
									7,1																										
**F438S**							*GCK*-MODY															0	1	D	2,75	51	D	D	-2,37	C65	0	154,81	-6,27		[2]
**F438Y**							*GCK*-MODY															0,01	1	N	0,66	15	D	D	-1,75	C15	0	21,61	-4,57		[2]
**E440G**	10,85	1,52	0,74	51,55	0,41	5,82	*GCK*-MODY †	10	6,1	7.0												0,03	0,564	N	0,62	-40	N	N	-1,26	C35	29,27	85,24	-1,46	[88]	[88]
								8	5,8	5,1																									
**S441L**							PNDM						84	17		9.7						0	1	D	0,55	53	D	D	-0,43	C65	0	144,08	-6,85		[12]
**S441W**	15,89	1,38	1,12	20,72	0,11	6,63	*GCK*-MODY	10	6,7	7,9												0	1	D	0,07	67	D	D	-0,58	C65	0	176,58	-7,01	[88]	[88]
								9	7,6	8,2																									[16]
**E442K**	4,43	1,6	0,72	41,98	3,33	4,14	PHHI										1	1,5				0,01	0,996	N	-0,15	17	N	D	-0,69	C55	0	55,87	-4,06	[88]	[88]
**S445C**							*GCK*-MODY															0	1	D	-0,45	13	D	D	-1,2	C65	0	111,67	-5,71		[2]
**G446R**							*GCK*-MODY	14	7,6													0	1	D	0,15	91	D	D	-1,22	C65	0	125,13	-7,73		[36]
**R447G**							*GCK*-MODY															0	1	D	1,48	59	D	D	-1,84	C65	0	125,13	-6,69		[89]
**R447Q**							*GCK*-MODY	9	6.7	5.5												0	1	D	0,59	35	N	N	-1,37	C35	0	42,81	-4,45		[36]
								7	6,7			6.3																							[8]
**A449S**							*GCK*-MODY	21	6.0													0	1	D	1,35	8	D	D	-1,5	C65	0	99,13	-4,67		[53]
**A449T**	1,33	0,95	1,81	3,21	1,23	6.7 *	*GCK*-MODY	27	7,4													0,02	1	D	1,12	51	D	D	-1,44	C55	0	58,02	-5,12	[47]	[47]
							PNDM						98	17.4	99.3	7.6																			[12]
**A450D**							*GCK*-MODY															0	1	D	2,16	72	D	D	-1,23	C65	0	125,75	-8,52		[2]
**A450T**							*GCK*-MODY															0	1	D	1,7	1	D	D	-1,09	C55	0	58,02	-5,97		[2]
**A450V**							*GCK*-MODY															0	1	D	0,66	33	D	D	-0,65	C55	0	64,43	-5,35		[20]
**V452L**	2,57	1,34	0,58	41,7	5,75	3	PHHI										1	2,2				0	0,012	N	0,84	-57	N	N	-1,02	C0	29,61	4,86	-3,39	[27,31]	[31]
**S453L**	16	1,39	0,69	8,17	0,22	6.8 *	*GCK*-MODY	30	7.1													0	1	N	0,53	18	N	D	-0,53	C65	0	144,08	-3,92	[24]	[36]
**S453W**							*GCK*-MODY															0	1	D	-0,09	46	D	D	-0,88	C65	0	176,58	-6,45		[2]
**A454E**	24,49	1,39	1,36	6,7	0,04	7	*GCK*-MODY															0	1	D	1,89	62	D	D	-1,02	C65	0	106,71	-7,74	This study	[2]
**A454T**							*GCK*-MODY															0	1	D	1,21	12	D	D	-0,97	C55	0	58,02	-6,44		[20]
**A454V**							*GCK*-MODY	31	6.3	9.1												0	1	D	0,85	27	D	D	-0,33	C55	0	64,43	-5,74		[36]
**V455E**							*GCK*-MODY	47,5	5,7	7,5		5,8										0	0	D	0,33	23	D	D	-1,45	C65	0	121,33	-7,44		[48]
**V455M**	3,18	1,66	0,25	48,6	6,27		PHHI										11315	2,5				0	0,229	D	0,33	-81	D	D	-0,83	C15	0	21,52	-4,04	[17]	[90]
	3,24	1,62	0,38	61,8	4,82	2,8											1642	1,3																[27]	
	3,02	1,62	0,37	53	5,23	2,7											1004	1,6																[13]	
																	13140	2,1																	
**A456V**	2,53	1,21	0,15	84,9	17,44	1,5	PHHI										Infancy	2.1 - 2.7		1519	3,8	0	0,741	D	0,96	-32	N	D	-0,43	C55	0	64,43	-3,71	[91]	[91]
	1,81	1,25	0,28	41.54 *	22.66 *												1095	0,9																[66]	[92]
	1,91	1,37	0,35	65,6	14,3	1,65												1,6																[27]	
	2,03	1,4	0,25	80,3	17,06	1,45												1,8																[13]	
**C457V**	5,9	1,15	0,65	26.36 *	3.85 *	4 *																0,34	0,084	N	1,28	-80	N	N	-0,41	C0	193,72	13,73	-2,42	[66]	
**C457W**							*GCK*-MODY	22														0,03	0,96	D	0,85	-10	D	D	-0,67	C0	193,72	34,82	-4,42		[93]
								53																											
**K458R**	12,28	1,34	0,81	27.95 *	0.93 *	5.8 *																1	0,393	N	-0,08	-92	N	N	-0,26	C25	0	26	-0,94	[66]	
**K459L**	7,03	1,65	0,6	42.82 *	1.76 *	4.3 *																1	0	N	-0,22	-84	N	N	-0,22	C0	94,49	12,95		[66]	
**A460R**	1,94	1,1	0,55	29.87 *	15.04 *	1.6 *																0,02	0,943	D	0,71	-43	N	D	-0,75	C65	0	111,26		[66]	
**M462I**							*GCK*-MODY															0,03	0	N		-57	N	N		C0	14,3	0			[2]
**G464D**							*GCK*-MODY															0,04	0,012	N		50	N	N		C0	115,24	61,5			[2]
**Q465R**							*GCK*-MODY															0,06	0,281	N		-91	N	N		C0	83,4	28,37			[2]

* The data were calculated in this study according to previously published kinetic variables and the corresponding wild-type GCK characteristics
† Atypical MODY

Abbreviations:

D disease-related effect
FPG fasting plasma glucose
GD Grantham Deviation
GSIR glucose-stimulated insulin release
GV Grantham Variation
IGT impaired glucose tolerance
N neutral effect
NA no activity
NN non-neutral effect
N/D not clear, whether the effect is disease-related or neutral
PG plasma glucose
PHHI persistent hyperinsulinemic hypoglycemia of infancy
PNDM permanent neonatal diabetes mellitus

References for the Suppl. Table S2:

1. Ziemssen F, Bellanné-Chantelot C, Osterhoff M, Schatz H, Pfeiffer AFH (2002) To: Lindner T, Cockburn BN, Bell GI (1999). Molecular genetics of MODY in Germany. Diabetologia 42:121–123. Diabetologia 45:286–287
2. Osbak KK, Colclough K, Saint-Martin C, et al. (2009) Update on mutations in glucokinase (GCK), which cause maturity-onset diabetes of the young, permanent neonatal diabetes, and hyperinsulinemic hypoglycemia. Hum Mutat 30:1512–1526
3. Pihoker C, Gilliam LK, Ellard S, et al. (2013) Prevalence, characterictics and clinical diagnosis of maturity-onset diabetes of the young due to mutations in HNF1A, HNF4A, and glucokinase: results from the SEARCH for diabetes in youth. J Clin Endocrinol Metab 98:4055–4062
4. Weinert LS, Silveiro SP, Giuffrida FMA, et al. (2014) Three unreported glucokinase (GCK) missense mutations detected in the screening of thirty-two Brazilian kindreds for GCK and HNF1A-MODY. Diabetes Res Clin Pract 106:e44–e48
5. Lukášová P, Včelák J, Vaňková M, et al. (2008) Screening of mutations and polymorphisms in the glucokinase gene in Czech diabetic and healthy control populations. Physiol Res 57(Suppl 1):S99–S108
6. Kuraeva TL, Sechko EA, Zilberman LI, et al. (2015) [Molecular genetics and clinical variants MODY2 and MODY3 in children in Russia]. Probl Endocrinol 61:14–25 (in Russian)
7. Agladioglu SY, Aycan Z, Cetinkaya S, et al. (2016) Maturity onset diabetes of youth (MODY) in Turkish children: sequence analysis of 11 causative genes by next generation sequencing. J Pediatr Endocrinol Metab 29:487–496
8. Vits L, Beckers D, Craen M, et al. (2006) Identification of novel and recurrent glucokinase mutations in Belgian and Luxembourg maturity onset diabetes of the young patients. Clin Genet 70:355–359
9. Valentínová L, Beer NL, Staník J, et al. (2012) Identification and functional characterisation of novel glucokinase mutations causing maturity-onset diabetes of the young in Slovakia. PLoS ONE 7:e34541
10. Prisco F, Iafusco D, Franzese A, Sulli N, Barbetti F (2000) MODY 2 presenting as neonatal hyperglycaemia: a need to reshape the definition of "neonatal diabetes"? Diabetologia 43:1331–1332
11. Capuano M, Garcia-Herrero CM, Tinto N, et al. (2012) Glucokinase (GCK) mutations and their characterization in MODY2 children of Southern Italy. PLoS ONE 7:e38906
12. Raimondo A, Chakera AJ, Thomsen SK, et al. (2014) Phenotypic severity of homozygous GCK mutations causing neonatal or childhood-onset diabetes is primarily mediated through effects on protein stability. Hum Mol Genet 23:6432–6440
13. Gloyn AL, Odili S, Buettger C, Njolstad PR, Shiota C, Magnuson MA, Matschinsky FM (2004) Glucokinase and the regulation of blood sugar. In: Matschinsky FM, Magnuson MA (eds); Glucokinase and glycemic disease: from basics to novel therapeutics. Frontiers in Diabetes 16. Karger, Basel, pp 92–109
14. Borowiec M, Antosik K, Fendler W, et al. (2012) Novel glucokinase mutations in patients with monogenic diabetes – clinical outline of GCK-MD and potential for founder effect in Slavic population. Clin Genet 81:278–283
15. Carmody D, Naylor RN, Bell CD, et al. (2016) GCK-MODY in the US National Monogenic Diabetes Registry: frequently misdiagnosed and unnecessarily treated. Acta Diabetol 53:703–708
16. Massa O, Meschi F, Cuesta-Muñoz A, et al. (2001) High prevalence of glucokinase mutations in Italian children with MODY. Influence on glucose tolerance, first-phase insulin response, insulin sensitivity and BMI. Diabetologia 44:898–905
17. Davis EA, Cuesta-Muñoz A, Raoul, et al. (1999) Mutants of glucokinase cause hypoglycaemia- and hyperglycaemia syndromes and their analysis illuminates fundamental quantitative concepts of glucose homeostasis. Diabetologia 42:1175–1186
18. Miller SP, Anand GR, Karschnia EJ, Bell GI, LaPorte DC, Lange AJ (1999) Characterization of glucokinase mutations associated with maturity-onset diabetes of the young type 2 (MODY-2): different glucokinase defects lead to a common phenotype. Diabetes 48:1645–1651
19. Stanik J, Dusatkova P, Cinek O, et al. (2014) De novo mutations of GCK, HNF1A and HNF4A may be more frequent in MODY than previously assumed. Diabetologia 57:480–484
20. Borowiec M, Fendler W, Antosik K, et al. (2012) Doubling the referral rate of monogenic diabetes through a nationwide information campaign – update on glucokinase gene mutations in a Polish cohort. Clin Genet 82:587–590
21. Haliloglu B, Hysenaj G, Atay Z, et al. (2016) GCK gene mutations are a common cause of childhood-onset MODY (maturity-onset diabetes of the young) in Turkey. Clin Endocrinol 85:393–399
22. Yorifuji T, Fujimaru R, Hosokawa Y, et al. (2012) Comprehensive molecular analysis of Japanese patients with pediatric-onset MODY-type diabetes mellitus. Pediatr Diabetes 13:26–32
23. Estalella I, Garcia-Gimeno MA, Marina A, Castaño L, Sanz P (2008) Biochemical characterization of novel glucokinase mutations isolated from Spanish maturity-onset diabetes of the young (MODY2) patients. J Hum Genet 53:460–466

24. Sagen JV, Odili S, Bjorkhaug L, et al. (2006) From clinicogenetic studies of maturity-onset diabetes of the young to unraveling complex mechanisms of glucokinase regulation. Diabetes 55:1713–1722
25. Njolstad PR, Cockburn BN, Bell GI, Sovik O (1998) A missense mutation, Val62Ala, in the glucokinase gene in a Norwegian family with maturity-onset diabetes of the young. Acta Paediatr 87:853–856
26. Gloyn AL, Odili S, Zelent D, et al. (2005) Insights into the structure and regulation of glucokinase from a novel mutation (V62M), which causes maturity-onset diabetes of the young. J Biol Chem 14:14105–14113
27. Matschinsky FM (2009) Assessing the potential of glucokinase activators in diabetes therapy. Nat Rev 8:399–416
28. Christesen HBT, Tribble ND, Molven A, et al. (2008) Activating glucokinase (GCK) mutations as a cause of medically responsive congenital hyperinsulinism: prevalence in children and characterisation of a novel GCK mutation. Eur J Endocrinol 159:27–34
29. Gloyn AL, Noordam K, Willemsen MAAP, et al. (2003) Insights into the biochemical and genetic basis of glucokinase activation from naturally occuring hypoglycemia mutations. Diabetes 52:2433–2440
30. Wabitsch M, Lahr G, van de Bunt M, et al. (2007) Heterogeneity in disease severity in a family with a novel G68V GCK activating mutation causing persistent hyperinsulinaemic hypoglycaemia of infancy. Diabet Med 24:1393–1399
31. Meissner T, Marquard J, Cobo-Vuilleumier N, et al. (2009) Diagnostic difficulties in glucokinase hyperinsulinism. Horm Metab Res 41:320–326
32. Tinto N, Zagari A, Capuano M, et al. (2008) Glucokinase gene mutations: structural and genotype-phenotype analyses in MODY children from South Italy. PLoS ONE 3:e1870
33. Cao H, Shorey S, Robinson J, et al. (2002) GCK and HNF1A mutations in Canadian families with maturity onset diabetes of the young (MODY). Hum Mutat 20:478–479
34. Gozlan Y, Tenenbaum A, Shalitin S, et al. (2012) The glucokinase mutation p.T206P is common among MODY patients of Jewish Ashkenazi descent. Pediatr Diabetes 13:e14–e21
35. Li Q, Cao X, Qiu HY, Lu J, et al. (2016) A three-step programmed method for the identification of causative gene mutations of maturity onset diabetes of the young (MODY). Gene 588:141–148
36. Thomson KL, Gloyn AL, Colclough K, et al. (2003) Identification of 21 novel glucokinase (GCK) mutations in UK and European Caucasians with maturity-onset diabetes of the young (MODY). Hum Mutat 22:417
37. Yokota I, Moritani M, Nishisho K, Miyoshi T, Kotani Y, Kagami S (2011) Detection of glucokinase gene defects in non-obese Japanese children diagnosed with diabetes by school medical examinations. Endocr J 58:741–746
38. Pruhova S, Dusatkova P, Sumnik Z, et al. (2010) Glucokinase diabetes in 103 families from a country-based study in the Czech Republic: geographically restricted distribution of two prevalent GCK mutations. Pediatr Diabetes 11:529–535
39. Marotta DE, Anand GR, Anderson TA, et al. (2005) Identification and characterization of the ATP-binding site in human pancreatic glucokinase. Arch Biochem Biophys 436:23–31
40. Marks SD, Couch RM (2010) Identification of two new mutations in the glucokinase gene that result in maturity-onset diabetes of the young. Diabetes Care 33:e94
41. Martínez R, Gutierrez-Nogués A, Fernández-Ramos C, et al. (2017) Heterogeneity in phenotype of hyperinsulinism caused by activating glucokinase mutations: a novel mutation and its characterization. Clin Endocrinol, doi: 10.1111/cen.13318
42. Molnes J, Bjorkhaug L, Sovik O, Njolstad PR, Flatmark T (2008) Catalytic activation of human glucokinase by substrate binding - residue contacts involved in the binding of D-glucose to the super-open form and conformational transitions. FEBS J 275:2467–2481
43. Beer NL, van de Bunt M, Colclough K, et al. (2011) Discovery of a novel site regulating glucokinase activity following characterization of a new mutation causing hyperinsulinemic hypoglycemia in humans. J Biol Chem 286:19118–19126
44. Ng MCY, Cockburn BN, Lindner TH, et al. (1999) Molecular genetics of diabetes mellitus in Chinese subjects: identification of mutations in glucokinase and hepatocyte nuclear factor-1α genes in patients with early-onset Type 2 diabetes mellitus/MODY. Diabet Med 16:956–963
45. Bennett JT, Vasta V, Zhang M, NarayananJ, Gerrits P, Hahn SH (2015) Molecular genetic testing of patients with monogenic diabetes and hyperinsulinism. Mol Genet Metab 114:451–458
46. Żurawek M, Wender-Ożegowska E, Januszkiewicz-Lewandowska D, Zawiejska A, Nowak J (2007) GCK and HNF1α mutations and polymorphisms in Polish women with gestational diabetes. Diabetes Res Clin Pract 76:157–158
47. García-Herrero CM, Rubio-Cabezas O, Azriel S, et al. (2012) Functional characterization of MODY2 mutations highlights the importance of the fine-tuning of glucokinase and its role in glucose sensing. PLoS ONE 7:e30518
48. Gloyn AL, van de Bunt M, Stratton IM, et al. (2009) Prevalence of GCK mutations in individuals screened for fasting hyperglycaemia. Diabetologia 52:172–174
49. Toaima D, Näke A, Wendenburg J, et al. (2005) Identification of novel GCK and HNF1A/TCF1 mutations and polymorphisms in German families with maturity-onset diabetes of the young (MODY). Hum Mutat 25:503–504
50. Molnes J, Teigen K, Aukrust I, et al. (2011) Binding of ATP at the active site of human pancreatic glucokinase - nucleotide-induced conformational changes with possible implications for its kinetic cooperativity. FEBS J 278:2372–2386
51. Hattersley AT, Beards F, Ballantyne E, Appleton M, Harvey R, Ellard S (1998) Mutations in the glucokinase gene of the fetus result in reduced birth weight. Nat Genet 19:268–270
52. Ellard S, Beards F, Allen LIS, et al. (2000) A high prevalence of glucokinase mutations in gestational diabetic subjects selected by clinical criteria. Diabetologia 43:250–253
53. Solera J, Arias P, Amiñoso, et al. (2009) Identification of eight new mutations in the GCK gene by DHPLC screening in a Spanish population. Diabetes Res Clin Pract 85:20–23
54. Yilmaz AA, Elmaogullari S, Demirel F, et al. (2016) A novel glucokinase gen mutation: MODY type-2 case. Horm Res Paediatr 86(Suppl 1):247
55. Nam JH, Lee HC, Kim YH, et al. (2000) Identification of glucokinase mutation in subjects with post-renal transplantation diabetes mellitus. Diabetes Res Clin Pract 50:169–176
56. Galán M, Vincent O, Roncero I, et al. (2006) Effects of novel maturity-onset diabetes of the young (MODY)-associated mutations on glucokinase activity and protein stability. Biochem J 393:389–396
57. Cappelli A, Tumini S, Consoli A, et al. (2009) Novel mutations in GCK and HNF1A genes in Italian families with MODY phenotype. Diabetes Res Clin Pract 83:e72–e74
58. Guazzarotti L, Fumelli P, Testa I, et al. (2001) Diagnosis of MODY in the offspring of parents with insulin-dependent and non-insulin-dependent diabetes mellitus. J Pediatr Endocrinol Metab 14 (Suppl 1):611–617
59. Fahr K, Böckmann A, Wildhardt G, Gessler P (2012) MODY 2 diabetes in a mature newborn due to a new mutation in the GCK-gene. Klin Padiatr 224:316–317
60. Giuffrida FMA, Moises RS, Weinert LS, et al. (2017) Maturity-onset diabetes of the young (MODY) in Brazil: establishment of a national registry and appraisal of available genetic and clinical data. Diabetes Res Clin Pract 123:134–142
61. Kawakita R, Hosokawa Y, Fujimaru R, et al. (2014) Molecular and clinical characterization of glucokinase maturity-onset diabetes of the young (GCK-MODY) in Japanese patients. Diabet Med 31:1357–1362
62. Shimada F, Makino H, Hashimoto N, et al. (1993) Type 2 (non-insulin-dependent) diabetes mellitus associated with a mutation of the glucokinase gene in a Japanese family. Diabetologia 36:433–437
63. Codner E, Deng L, Pérez-Bravo F, et al. (2006) Glucokinase mutations in young children with hyperglycemia. Diabetes Metab Res Rev 22:348–355
64. Morishita K, Kyo C, Yonemoto T, Kosugi R, Ogawa T, Inoue T (2017) Asymptomatic congenital hyperinsulinism due to a glucokinase-activating mutation, treated as adrenal insufficiency for twelve years. Case Rep Endocrinol 2017:4709262
65. Kanthimathi S, Jahnavi S, Balamurugan K, et al. (2014) Glucokinase gene mutations (MODY 2) in Asian Indians. Diabetes Technol Ther 16:180–185
66. Pedelini L, Garcia-Gimeno MA, Marina A, et al. (2005) Structure-function analysis of the α5 and the α13 helices of human glucokinase: description of two novel activating mutations. Protein Sci 14:2080–2086
67. Njolstad PR, Sovik O, Cuesta-Muñoz, et al. (2001) Neonatal diabetes mellitus due to complete glucokinase deficiency. N Engl J Med 344:1588–1592
68. Henquin JC, Sempoux C, Marchandise J, et al. (2013) Congenital hyperinsulinism caused by hexokinase I expression or glucokinase-activating mutation in a subset of β-cells. Diabetes 62:1689–1696
69. Cuesta-Muñoz AL, Huopio H, Otonkoski T, et al. (2004) Severe persistent hyperinsulinemic hypoglycaemia due to a de novo glucokinase mutation. Diabetes 53:2164–2168
70. Beer NL, Osbak KK, van de Bunt, et al. (2012) Insights into the pathogenicity of rare missense GCK variants from the identification and functional characterization of compound heterozygous and double mutations inherited in cis. Diabetes Care 35:1482–1484
71. Shoemaker AH, Zienkiewicz J, Moore DJ (2012) Clinical assessment of HNF1A and GCK variants and identification of a novel mutation causing MODY2. Diabetes Res Clin Pract 96:e36–e39
72. Berger M, Mönks D, Schmidt H, et al. (2005) Are glucokinase mutations associated with low triglycerides? Clin Chem 51:791–793
73. García-Herrero CM, Galán M, Vincent O, et al. (2007) Functional analysis of human glucokinase gene mutations causing MODY2: exploring the regulatory mechanisms of glucokinase activity. Diabetologia 50:325–333
74. Constantini S, Malerba G, Contreas G, et al. (2015) Genetic and bioinformatics analysis of four novel GCK missense variants detected in Caucasian families with GCK-MODY phenotype. Clin Genet 87:440–447
75. Milenkovič T, Zdravković D, Mitrović K (2008) [Novel glucokinase mutation in a boy with maturity-onset diabetes of the young]. Srp Arh Celok Lek 136:542–544 (in Serbian)
76. Zouali H, Vaxillaire M, Lesage S, et al. (1993) Linkage analysis and molecular scanning of glucokinase gene in NIDDM families. Diabetes 42:1238–1245
77. Sakura H, Eto K, Kadowaki H, et al. (1992) Structure of the human glucokinase gene and identification of a missense mutation in a Japanese patient with early-onset non-insulin-dependent diabetes mellitus. J Clin Endocrinol Metab 75:1571–1573
78. Njolstad PR, Sagen JV, Bjorkhaug L, et al. (2003) Permanent neonatal diabetes caused by glucokinase deficiency: inborn error of the glucose-insulin signaling pathway. Diabetes 52:2854–2860
79. Wang Z, Ping F, Zhang Q, et al. (2017) Preliminary screening of mutations in the glucokinase gene of Chinese patients with gestational diabetes. J Diabetes Investig, doi: 10.1111/jdi.12664
80. Heggie AJ, Walker M (2016) A patient with a glucokinase mutation: a cautionary tale. Diabet Med 33(Suppl 1):102
81. Pulst K, Arbo T, Kahre T, Peet A, Tillmann V (2012) MODY2 caused by a novel mutation of GCK gene. J Pediatr Endocrinol Metab 25:801–803
82. Papadimitriou DT, Willems PJ, Bothou C, Karpathios T, Papadimitriou A (2015) A novel heterozygous mutation in the glucokinase gene is responsible for an early-onset mild form of maturity-onset diabetes of the young, type 2. Diabetes Metab 41:342–345
83. Yellapu NK, Valasani KR, Pasupuleti SK, Gopal S, Potukuchi Venkata Gurunadha Krishna S, Matcha B (2014) Identification and analysis of novel R308K mutation in glucokinase of type 2 diabetic patient and its kinetic correlation. Biotechnol Appl Biochem 61:572–581
84. Shen Y, Cai M, Liang H, Wang H, Weng J (2011) Insight into the biochemical characteristics of a novel glucokinase gene mutation. Hum Genet 129:231–238
85. Steele AM, Tribble ND, Caswell R, et al. (2011) The previously reported T342P GCK missense variant is not a pathogenic mutation causing MODY. Diabetologia 54:2202–2205
86. Jha S, Siddiqui S, Waghdhare S, et al. (2016) Identification of a novel glucokinase mutation in an Indian woman with GCK-MODY. Lancet Diabetes Endocrinol 4:302
87. Doddabelavangala Mruthyunjaya M, Chapla A, Hesarghatta Shyamasunder A, et al. (2017) Comprehensive maturity onset diabetes of the young (MODY) gene screening in pregnant women with diabetes in India. PLoS ONE 12:e0168656
88. Barbetti F, Cobo-Vuilleumier N, Dionisi-Vici C, et al. (2009) Opposite clinical phenotypes of glucokinase disease: description of a novel activating mutation and contiguous inactivating mutations in human glucokinase (GCK) gene. Mol Endocrinol 23:1983–1989
89. Esquiaveto-Aun AM, De Mello MP, Paulino MF, Minicucci WJ, Guerra-Júnior, De Lemos-Marini (2015) A new compound heterozygosis for inactivating mutations in the glucokinase gene as cause of permanent neonatal diabetes mellitus (PNDM) in double-first cousins. Diabetol Metab Syndr 7:101
90. Glaser B, Kesavan P, Heyman M, et al. (1998) Familial hyperinsulinism caused by an activating glucokinase mutation. N Engl J Med 338:226–230
91. Christesen HBT, Jacobsen BB, Odili S, et al. (2002) The second activating glucokinase mutation (A456V): implications for glucose homeostasis and diabetes therapy. Diabetes 51:1240–1246
92. Dullaart RP, Hoogenberg K, Rouweé CW, Stulp BK (2004) Family with autosomal dominant hyperinsulinism associated with A456V mutation in the glucokinase gene. J Intern Med 255:143–145
93. Furuzawa GK, Giuffrida FMA, Oliveira CSV, Chacra AR, Dib SA, Reis AR (2008) Low prevalence of MODY2 and MODY3 mutations in Brazilian individuals with clinical MODY phenotype. Diabetes Res Clin Pract 81:e12–e14

Suppl. Table S3. Nonsynonymous substitutions analyzed in vitro in this study and the corresponding primers used in site-directed mutagenesis.

Nonsynonymous substitution			Primers
V33A	c.98T>C	Fw	GGAGGACCTGAAGAAGGCGATGAGACGGATG
		Rv	CGTCTCATCGCCTTCTTCAGGTCCTCC
R63S	c.187C>T	Fw	CACCTACGTGTGCTCCACCCCAGAAGGCTCAGAAGTC
		Rv	AGCCTTCTGGGGTGGAGCACACGTAGGTGGGCAGC
G81D	c.242G>A	Fw	GACCTGGGTGACACTAACTTCAGGGTG
		Rv	GAAGTTAGTGTCACCCAGGTCCAGGGAGAGGAAGTCCCC<br>GACTTCTG
F150L	c.450C>A	Fw	GGGCTTCACCTTATCCTTTCCTGTGAGGC
		Rv	GCCTCACAGGAAAGGATAAGGTGAAGCCC
T209K	c.626C>A	Fw	CACGGTGGCCAAGATGATCTCCTGCTACTAC
		Rv	GCAGGAGATCATCTTGGCCACCGTGTCATTC
R250C	c.748C>T	Fw	GGACGAGGGCTGCATGTGCGTCAATACCGAG
		Rv	GACGCACATGCAGCCCTCGTCCCCCTCCAC
M251C	c.751_753<br>delinsTGT	Fw	GACGAGGGCCGCTGTTGCGTCAATACCGAGTGGGGC
		Rv	CGGTATTGACGCAACAGCGGCCCTCGTCCCCCTCC
M251I	c.753G>A	Fw	AGGGGGACGAGGGCCGCATATGCGTCAATACCGAGTGGG
		Rv	CCACTCGGTATTGACGCATATGCGGCCCTCGTCCCC
M251V	c.751A>G	Fw	CGAGGGCCGCGTGTGCGTCAATACCGAGTG
		Rv	CGGTATTGACGCACACGCGGCCCTCGTCCC
C252R	c.754T>C	Fw	GAGGGCCGCATGCGCGTCAATACCGAGTGGG
		Rv	CGGTATTGACGCGCATGCGGCCCTCGTCCCC
F260L	c.778T>C	Fw	GAGTGGGGCGCCTCCGGGGACTCCGGCGAG
		Rv	CTCGCCGGAGTCCCCGAGGGCGCCCCACTC
G295D	c.884G>A	Fw	GAAGCTCATAGGTGACAAGTACATGGGCGAGCTGG
		Rv	CCAGCTCGCCCATGTACTTGTCACCTATGAGCTTC
L314P	c.941T>C	Fw	GGACGAAAACCCGCTCTTCCACGGGGAG
		Rv	CTCCCCGTGGAAGAGCGGGTTTTCGTCC
F316V	c.946T>G	Fw	GAAAACCTGCTCGTCCACGGGGAGGCCTCCGAGCAGCTG<br>CGCACACG
		Rv	CTCCCCGTGGACGAGCAGGTTTTCGTCC
G318R	c.952G>A	Fw	CTCTTCCACAGGGAGGCCTCCGAGCAGCTGCGCACACG
		Rv	CGTGTGCGCAGCTGCTCGGAGGCCTCCCTGTGGAAGAG
G385W	c.1153G>T	Fw	GTGCTCGGCGTGGCTGGCGGGCGTCATC
		Rv	GATGACGCCCGCCAGCCACGCCGAGCAC
F419L	c.1255T>C	Fw	CAAGCTGCACCCCAGCCTCAAGGAGCGGTTCC
		Rv	GGAACCGCTCCTTGAGGCTGGGGTGCAGCTTG
C434Y	c.1301G>A	Fw	GGCTGACGCCCAGCTACGAGATCACCTTCATCG
		Rv	CGATGAAGGTGATCTCGTAGCTGGGCGTCAGCC
A454E	c.1361C>A	Fw	GGCCCTGGTCTCGGAGGTGGCCTGTAAGAAG
		Rv	CTTCTTACAGGCCACCTCCGAGACCAGGGCC
